# Mapping the Interaction Sites between AMPA Receptors and TARPs Reveals a Role for the Receptor N-Terminal Domain in Channel Gating

**DOI:** 10.1016/j.celrep.2014.09.029

**Published:** 2014-10-16

**Authors:** Ondrej Cais, Beatriz Herguedas, Karolina Krol, Stuart G. Cull-Candy, Mark Farrant, Ingo H. Greger

**Affiliations:** 1 Neurobiology Division, MRC Laboratory of Molecular Biology, Cambridge CB2 0QH, UK; 2 Department of Neuroscience, Physiology and Pharmacology, University College London, London WC1E 6BT, UK

## Abstract

AMPA-type glutamate receptors (AMPARs) mediate fast neurotransmission at excitatory synapses. The extent and fidelity of postsynaptic depolarization triggered by AMPAR activation are shaped by AMPAR auxiliary subunits, including the transmembrane AMPAR regulatory proteins (TARPs). TARPs profoundly influence gating, an effect thought to be mediated by an interaction with the AMPAR ion channel and ligand binding domain (LBD). Here, we show that the distal N-terminal domain (NTD) contributes to TARP modulation. Alterations in the NTD-LBD linker result in TARP-dependent and TARP-selective changes in AMPAR gating. Using peptide arrays, we identify a TARP interaction region on the NTD and define the path of TARP contacts along the LBD surface. Moreover, we map key binding sites on the TARP itself and show that mutation of these residues mediates gating modulation. Our data reveal a TARP-dependent allosteric role for the AMPAR NTD and suggest that TARP binding triggers a drastic reorganization of the AMPAR complex.

## Highlights

• The NTD linker has a TARP-dependent and TARP-specific impact on AMPAR gating
• Peptide arrays reveal binding of TARPs to both extracellular domains of AMPARs
• A structural reorganization of AMPARs is triggered by TARP binding

## Graphical Abstract

**Figure:**
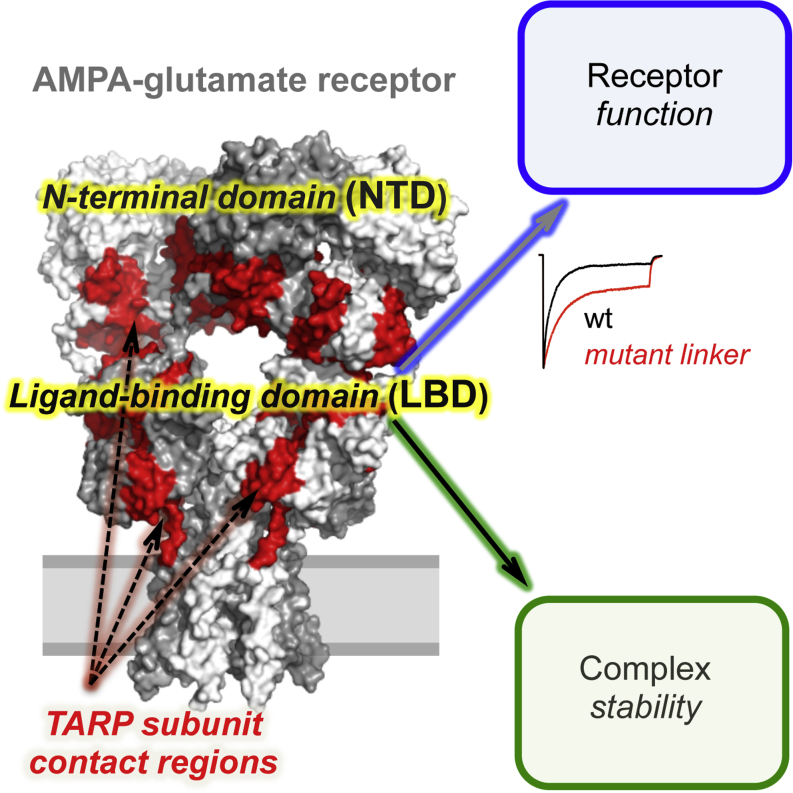

## Teaser

Gating properties of synaptic AMPA-type glutamate receptors (AMPARs) are modulated by the transmembrane AMPAR regulatory proteins (TARPs), yet knowledge about their binding on a molecular level is limited. Here, Cais et al. map this interaction on both partner molecules and reveal a functional role for the receptor N-terminal domain.

## Introduction

AMPA-type glutamate receptors (AMPARs) mediate fast excitatory transmission and are crucial for various forms of synaptic plasticity (Bredt and Nicoll, 2003; Cull-Candy et al., 2006). Their varied kinetic behavior (Mosbacher et al., 1994), as well as their calcium permeability and voltage-dependent block by polyamines (Cull-Candy et al., 2006; Geiger et al., 1995), varies between brain regions and appear to be adapted to the specific function of a given circuit (Jonas, 2000; Trussell, 1998). These properties depend on the nature and mRNA processing status of the four pore-forming subunits (GluA1–GluA4) (Traynelis et al., 2010; Jonas, 2000) and on the type and stoichiometry of AMPAR auxiliary subunits (Jackson and Nicoll, 2011).

Four families of auxiliary subunits have been identified: transmembrane AMPAR regulatory proteins (TARPs) (Tomita et al., 2005; Turetsky et al., 2005), cornichons (Schwenk et al., 2009), CKAMP44 (von Engelhardt et al., 2010), and GSG1L (Schwenk et al., 2012; Shanks et al., 2012). Most of these alter AMPAR gating and confer effects that can be specific for a given synapse or cell. TARPs were the first identified bona fide AMPAR auxiliary proteins, modifying both AMPAR function and trafficking. Based on their modulatory actions, TARPs have been classified as type 1a (γ-2 and γ-3), type 1b (γ-4 and γ-8), and type 2 (γ-5 and γ-7) (Kato et al., 2010). TARP-like modulation of AMPARs has also been seen in invertebrates (Walker et al., 2006; Wang et al., 2008) and thus appears highly conserved.

The precise nature of the AMPAR/TARP interaction and thus the mechanism underlying gating modulation are poorly understood. Both the AMPAR transmembrane region and the ligand binding domain (LBD) have been implicated in TARP interactions responsible for the modulation of ligand efficacy, pharmacology, gating, and pore properties (Jackson and Nicoll, 2011). Experiments using domain swapping between subtypes have identified TARP regions that are involved in regulating AMPARs. These include the extracellular loop (Ex1), the transmembrane sector, and the C terminus. Specifically, the TARP C tail appears critical for receptor trafficking and mediation of kinetic effects, while Ex1 influences both the efficacy of the partial agonist kainate and AMPAR kinetics (Tomita et al., 2005; Turetsky et al., 2005).

The most distal AMPAR domain, the N-terminal domain (NTD), is expected to be beyond the “reach” of the associated TARP. Apart from a role in subunit assembly, no clear function has been ascribed to this large and most sequence-diverse domain (Hansen et al., 2010; Kumar and Mayer, 2013), although deletion of the NTD slows desensitization kinetics (Bedoukian et al., 2006; Möykkynen et al., 2014; Pasternack et al., 2002). In stark contrast, the NTD of the N-methyl-D-aspartate (NMDA)-type glutamate receptor (NMDAR) mediates allosteric regulation of channel open probability (Paoletti, 2011) in a subunit-specific manner, rendering the NTD an important target for selective NMDAR drugs (Mony et al., 2009). NTD-mediated allostery in NMDARs has been shown to involve the ∼16-residue peptide linkers that connect the NTD to the LBD (Gielen et al., 2009; Mony et al., 2011; Yuan et al., 2009).

Here we show that the AMPAR NTD plays a previously unrecognized role in signaling. Shortening of the NTD-LBD linkers altered desensitization rates and recovery from the desensitized state and increased the steady-state response. These gating effects were TARP dependent and TARP specific. Using peptide arrays, we mapped the GluA2/TARP contact region and identified TARP binding sites on the NTD. On the LBD, TARP contact points mapped to functionally critical sites, including the ligand binding cleft, the flip/flop region, and the linkers that connect the LBD to the ion channel. We also determined the sites on the TARP that are contacted by the AMPAR and assessed their functional role using corresponding TARP mutants. Our results provide detailed insights into the molecular interactions of TARPs with AMPARs and show that these include the distal NTD. This subunit-specific TARP regulatory site may permit fine tuning of AMPAR signaling and provide a target for subunit-selective drugs.

## Results

### The NTD-LBD Linker Mediates TARP-Dependent Changes of AMPAR Gating

**Figure 1:**
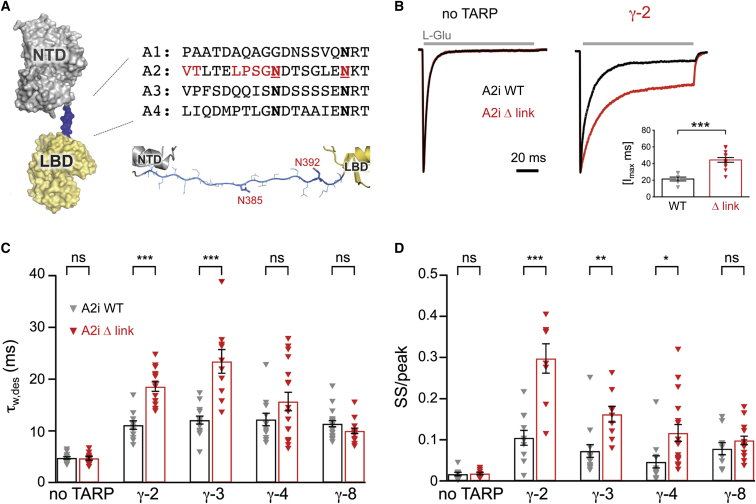
The NTD-LBD Linker Influences TARP-Dependent Changes in AMPAR Gating (A) Structure of the extracellular region of a GluA2 subunit, showing the NTD (gray), linker (blue), and LBD (yellow) (adapted from PDB: 3KG2). Sequence alignment of the rat GluA1-4 NTD linkers, red residues have been mutated in Δ link with VTxxxLPSG deleted and the two asparagines (N385 and N392 bold, underlined) mutated to Asp and Gln, respectively (analogous to PDB 3KG2). Model of a complete, stretched NTD linker is shown beneath the alignment. (B) Representative normalized current responses evoked by 100 ms glutamate application (gray bars). Outside-out patches were pulled from cells transfected with GluA2iQ WT or Δ link in the absence or presence of γ-2 and the decay of the current (−60 mV) analyzed to determine the time constant of desensitization and the magnitude of the steady state component. (Inset) Pooled data (mean ± SEM) showing the difference in charge transfer (normalized to the peak) during the 100 ms glutamate application (^∗∗∗^p < 0.001, Welch t test). (C) Pooled data showing the desensitization time constants for GluA2iQ WT or Δ link expressed alone (n = 14 and 10), or with TARPs γ-2 (n = 11 and 15), γ-3 (n = 14 and 10), γ-4 (n = 12 and 17), or γ-8 (n = 20 and 15). Currents were fitted with a two-exponential function. The weighted time constant (τ_w,des_) is shown ± SEM. Two-way ANOVA indicated a significant main effect of TARP (*F*_4, 111_ = 82.28, p = 2.66 × 10^−32^), no significant main effect of linker mutation (*F*_1, 111_ = 1.73, p = 0.19) and a significant interaction between linker and TARP effects (*F*_4, 111_ = 10.05, p = 5.61 × 10^−7^). Asterisks denote significance of difference between WT and Δ link for each TARP condition (^∗^p < 0.05, ^∗∗^p < 0.01, ^∗∗∗^p < 0.001; Welch t test). (D) Pooled data showing the ratio of current at the end of the 100 ms glutamate application (steady-state [SS]) to the peak response. The data are plotted and analyzed as in (C), for GluA2iQ WT or Δ link expressed alone (n = 10 each), or with γ-2 (n = 11 and 8), γ-3 (n = 14 and 9), γ-4 (n = 12 and 17), or γ-8 (n = 10 and 15). There were significant main effects of TARP subtype (*F*_4, 106_ = 42.74, p = 2.64 × 10^−21^) and linker mutation (*F*_1, 106_ = 5.04, p = 0.027) and a significant interaction between linker and TARP effects (*F*_4, 106_ = 8.89, p = 3.16 × 10^−6^). Asterisks are as in (C).

The iGluR extracellular region comprises two layers (Sobolevsky et al., 2009; Suzuki et al., 2014), a unique architecture not observed in other ligand-gated channels. In AMPARs, the function of the distal NTD layer is unknown. This layer is loosely connected to the LBD via ∼17 residue N-glycosylated linkers (Figure 1A). As these linkers may function as potential “output” regions for NTD-mediated allostery, we created linker mutations and assayed their effect on AMPAR function. Initially, we recreated the modifications that had been used in the GluA2 crystal structure, GluA2_cryst_ (Sobolevsky et al., 2009), as this provided direct structural information on the packing between NTD and LBD. Thus, we deleted six residues plus two N-glycosylation sites in GluA2(Q_607_) flip, resulting in the GluA2i linker mutation, Δ link (sequence in red; Figure 1A).

While the linker mutation produced no change in channel kinetics in the absence of TARP γ-2 (Figure 1B, left), in the presence of γ-2, the mutant exhibited a pronounced slowing of entry into the desensitized state (τ_w des_ 11.10 ± 0.82 ms for GluA2i wild-type [WT] versus 18.56 ± 0.92 ms for Δ link; n = 11 and 15, respectively) and an ∼3-fold increase in the steady-state current (Figures 1B–1D). These changes resulted in a more than 2-fold increase in normalized charge transfer of TARPed Δ link (inset in Figure 1B). Deactivation of the TARPed receptor was unaltered by the linker mutation (τ_w, deac_ 1.07 ± 0.10 ms for GluA2i WT versus 1.09 ± 0.16 ms for Δ link; n = 15 and 10, respectively).

To determine if this behavior was specific to γ-2, we measured desensitization kinetics of Δ link when associated with other TARPs. With γ-3, the effects of the linker mutation were similar to those seen with γ-2. However, in the presence of γ-8, the linker mutation neither slowed desensitization nor increased the steady-state component (Figures 1C and 1D). These data reveal that an alteration in the AMPAR NTD-LBD linker affects channel gating in a TARP-dependent and TARP-selective manner.

### The NTD-LBD Linker Modulates Recovery from Desensitization

**Figure 2:**
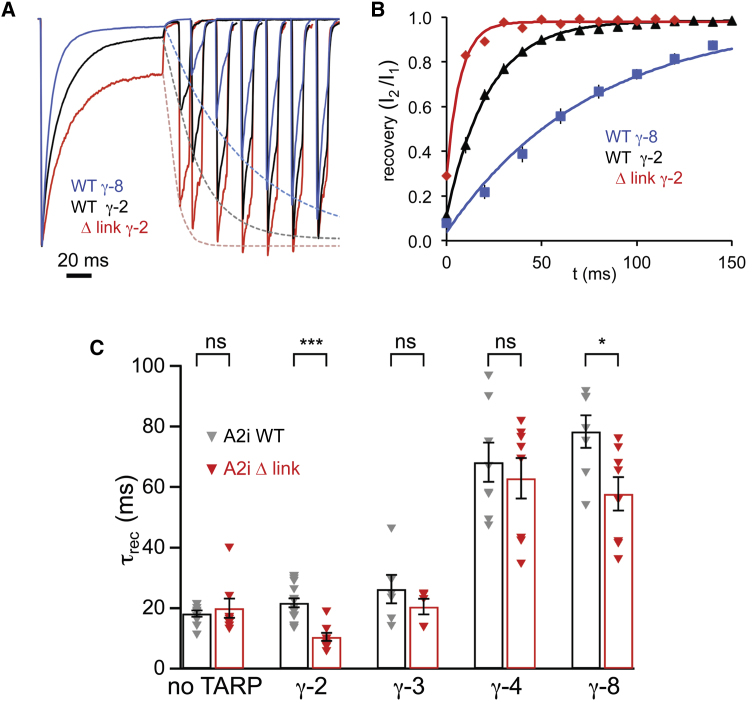
The Δ Link Mutation Accelerates Recovery from Desensitization in the Presence of TARPs (A) Representative traces illustrating recovery from desensitization (averages of three trials in each case). A 100 ms pulse of 10 mM L-glutamate was followed, at increasing intervals, by a 10 ms test pulse, and the recovery in the amplitude of the test response was fitted by a monoexponential function (dashed lines). Currents were normalized to the first peak, and for clarity, only selected traces are shown. (B) Summary of the data presented in (A). Relative currents at individual time points are shown ± SEM (error bars masked by the symbols). The solid lines are monoexponential fits of the average values (giving time constants of 7.3, 21.8, and 75.6 ms for GluA2 Δ link + γ-2, GluA2 WT + γ-2, and GluA2 WT + γ-8, respectively). (C) Pooled data for the time constant of recovery from desensitization (τ_rec_) for GluA2iQ WT and Δ link expressed alone (n = 11 and 8, respectively) or with TARPs γ-2 (n = 17 and 9), γ-3 (n = 6 and 5), γ-4 (n = 8 each), or γ-8 (n = 7 and 8) (shown ± SEM). Two-way ANOVA showed significant main effects of TARP subtype (*F*_4, 77_ = 62.61, p = 1.91 × 10^−23^) and linker mutation (*F*_1, 77_ = 25.58, p = 4.14 × 10^−6^) and a significant interaction between linker and TARP effects (*F*_4, 77_ = 3.14, p = 0.019). Asterisks denote significance of difference between WT and Δ link for each TARP condition (^∗^p < 0.05, ^∗∗∗^p < 0.001; Welch t test). See also Figure S1.

We next investigated whether the NTD linker has a wider role in AMPAR function and could affect other aspects of gating that are regulated by TARPs. TARPs are also known to accelerate recovery from desensitization for GluA1 receptors (Gill et al., 2012; Priel et al., 2005); our experiments showed that the influence of TARPs on recovery from desensitization depended both on the AMPAR subtype and the TARP isoform. Thus, in contrast with GluA1, recovery from desensitization of GluA2i was unaffected by the presence of γ-2 or γ-3 (type 1a TARPs) and was in fact markedly *slowed* by γ-4 and γ-8 (type 1b) (Figure 2 and Figure S1A available online). A similar pattern was also observed with GluA3i (Figure S1B), suggesting that accelerated recovery is specific to GluA1.

The Δ link mutation resulted in an acceleration of GluA2i recovery from desensitization. This effect was again TARP subtype specific and was observed with γ-2 and γ-8, but not with γ-3 or γ-4 (Figure 2). Accelerated recovery together with reduced desensitization (Figures 1B and 1C) is expected to boost charge transfer through TARPed Δ link. Thus, changes in the NTD-LBD linker have a wider role in AMPAR gating that is TARP dependent.

### Specific Features of the NTD-LBD Linker Mediate Gating Effects

**Figure 3:**
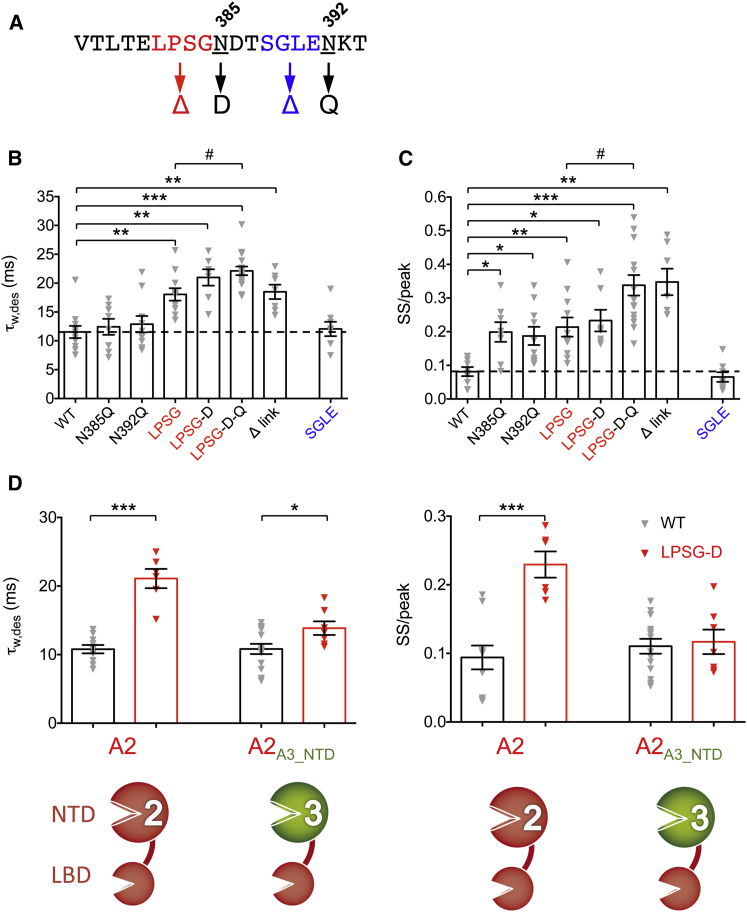
Gating Changes Are Mediated by Specific Structural Features of the NTD Linker (A) Sequence of the GluA2 NTD-LBD linker with the glycosylation sites and the amino acid quadruplets deleted in these experiments highlighted in red and blue. (B) Pooled data (mean ± SEM) showing the effects on desensitization (τ_w,des_) of NTD-LBD linker mutations in GluA2i in presence of γ-2. LPSG denotes a construct with these four amino acids deleted; LPSG-D combines this with the N385D mutation, and LPSG-D-Q additionally includes N392Q. Following one-way ANOVA (*F*_7, 25.7_ = 14.69, p = 1.52 × 10^−7^), pairwise comparisons showed that τ_w,des_ was slower for LPSG (n = 11), LPSG-D (n = 6), LPSG-D-Q (n = 16), and Δ link (n = 7) compared with WT (n = 11) (^∗∗^p < 0.01, ^∗∗∗^p < 0.001) and was significantly slower for LPSG-D-Q compared with LPSG (#p < 0.05) (Welch t tests). There was no significant effect of the glycosylation mutations N385D (n = 8) or N392Q (n = 10) and no effect of the alternative deletion mutant, SGLE (n = 8). (C) Pooled data for the steady state-to-peak ratio (SS/peak; presented and analyzed as in B). Following one-way ANOVA (*F*_7, 26.5_ = 17.18, p = 2.34 × 10^−8^), pairwise comparisons showed that the SS/peak ratio was greater for N385D (n = 8), N392Q (n = 10), LPSG (n = 11), LPSG-D (n = 7), LPSG-D-Q (n = 16), and Δ link (n = 7) compared with WT (n = 9) (^∗∗^p < 0.01, ^∗∗∗^p < 0.001) and significantly greater for LPSG-D-Q compared to LPSG (#p < 0.05) (Welch t tests). Again, no effect of the alternative deletion mutant, SGLE (n = 8). (D) Pooled data (± SEM) comparing the effects of the LPSG-D mutation in GluA2 and in a chimeric construct where the NTD of GluA2 was replaced by that of GluA3. (Left) τ_w,des_ was increased by LPSG-D in both GluA2 (n = 10 and 6) and GluA2_A3-NTD_ (n = 14 and 7) (^∗∗∗^p < 0.001 and ^∗^p < 0.05; Welch t tests). Two-way ANOVA showed a significant interaction between NTD and linker (*F*_1, 33_ = 11.62, p = 0.0017). Similar results were seen for the SS/peak ratio (right); in this case, the ratio was increased by LPSG-D in GluA2 (^∗∗∗^p < 0.001) but not in GluA2_A3-NTD_. Again, two-way ANOVA showed a significant interaction between NTD and linker (*F*_1, 33_ = 13.10, p = 9.75 × 10^−4^), confirming that the effect of the LPSG-D linker mutation was NTD-type specific. See also Figure S2.

We next pinpointed the minimal regions of the linker able to mediate gating effects. We focused on the core deletion, LPSG, and on two mutations (N385D and N392Q) that abolish N-glycosylation (Figure 3A) and thus may alter linker flexibility. Deletion of LPSG slowed GluA2i/γ-2 desensitization to a similar extent to that seen with the complete modification (Δ link) (Figure 3B). This phenotype was enhanced when combined with the glyco double null mutant (LPSG-N385D/N392Q), whereas mutation of the two glycosylation sites alone had no significant impact (Figure 3B). A similar trend was observed for the steady-state response except that N385D and N392Q alone also produced significant effects (Figure 3C). Hence, the four-residue linker deletion “LPSG” is necessary and sufficient to confer the alterations in gating seen with the Δ link mutant. As linkers have been suggested to encode structural states (Ma et al., 2011), our observation raised the question of whether these effects on gating were merely due to linker shortening or if they resulted from specific structural effects. To address this, we introduced an alternative four-residue deletion SGLE (S_388_-E_391_) further downstream (Figure 3A). Unlike ΔLPSG, the SGLE deletion did *not* slow GluA2i/γ-2 desensitization and did not increase the steady state current (Figures 3B and 3C), indicating that structural changes, rather than linker shortening alone, are important in mediating TARP-dependent alterations in GluA2i gating.

To extend this finding, we introduced a deletion into the linker of GluA3i, at a position analogous to ΔLPSG in GluA2i (Figure S2A). This deletion (ΔQISS) also slowed desensitization in the presence of γ-2, and its effect was magnified when combined with the glyco null mutation N387D (QISS-N387D; Figure S2B). However, as observed with GluA2i, a four-residue deletion introduced further downstream, ΔSSSE (analogous to GluA2i ΔSGLE), had no significant effect (Figure S2B). Hence, NTD-LBD linkers have a general role in the control of AMPAR gating.

### TARP-Dependent Reorientation of the NTD via the Linkers

How do NTD linker deletions affect gating of the AMPAR-TARP complex? While deletion of the NTD is known to alter desensitization kinetics (Bedoukian et al., 2006; Möykkynen et al., 2014; Pasternack et al., 2002), this domain has not been implicated in TARP modulation to date. In fact, previous work has questioned a role for the NTD in TARP function (Bedoukian et al., 2006; Morimoto-Tomita et al., 2009; Tomita et al., 2007). Similarly, in our hands, the gating properties of AMPARs lacking the NTD (GluA2i-ΔNTD), including desensitization kinetics and kainate efficacy, retained modulation by γ-2 (Figures S2C–S2E). However, these observations do not rule out a functional role for the NTD, which may trigger a TARP-dependent reorganization of the receptor (Figure S6B).

The position dependence of the deletions described in Figures 3A–3C suggests that the linker might facilitate a “preferred” orientation of the NTD relative to the LBD, perhaps to optimize TARP binding and thereby enable the TARP-dependent slowing of desensitization (Figure 1). As the NTD is highly sequence diverse, with a sequence identity of ∼55% between AMPAR subunits, we replaced the NTD core (lacking the linker) from GluA2i WT and GluA2i ΔLPSG-D with that of GluA3 (Figure 3D, bottom). We reasoned that if a selective positioning of NTD to LBD created an optimal TARP binding site, then this replacement would markedly alter this interaction surface. When GluA2i receptors contained the GluA3 NTD, the effects of the ΔLPSG-D mutation on desensitization and steady-state response were attenuated drastically (Figure 3D). This is consistent with the view that a specific orientation of the NTD-LBD may allow an optimal TARP interaction site and hence greater TARP efficacy.

### The NTD Stabilizes the AMPAR-TARP γ-2 Complex

**Figure 4:**
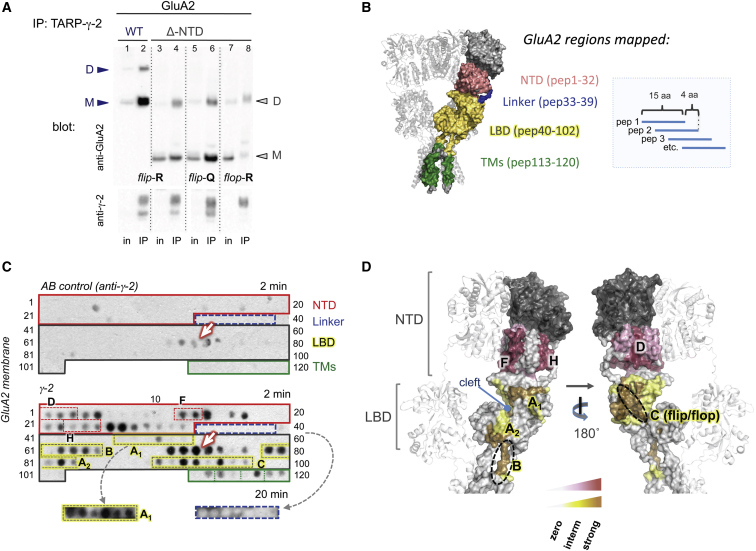
Mapping the TARP γ-2 Contact Region on GluA2 (A) coIP of GluA2 variants with TARP γ-2. The blot was probed with polyclonal GluA2 antibody (top panel) and anti γ-2 (bottom panel). Both WT and ΔNTD protein migrated as monomer (M) and dimer (D), denoted by arrowheads. Note that while inputs were comparable, amounts of IPed GluA2 varied between conditions. (B) Schematic of the peptide array layout (right). Each peptide is spotted onto a nitrocellulose membrane (C). Peptide coverage of the rat GluA2 sequence is outlined in the left panel in color code as indicated. The four GluA2 regions—the NTD lower lobe, the NTD-LBD linkers, the LBD, and the TM segments of the channel—are highlighted. GluA2 peptide numbers covering each domain are indicated in brackets. See Table S1 for peptide sequences. (C) Regions of GluA2 binding to TARP γ-2. (Upper panel) Nonspecific signal, resulting from anti-γ-2 antibody binding to GluA2 in the absence of the γ-2 probe (“AB control”). AMPAR domains are highlighted in boxes and match the color scheme in (B). Peptide numbers are indicated on the side. The membrane was exposed to an X-ray film for 2 min; the arrow denotes nonspecific signals. (Lower panel) The same membrane was probed with full-length TARP γ-2 and detected with anti-γ-2 AB followed by a HRP-labeled secondary AB (2 min exposure). Individual AMPAR secondary structure elements, corresponding to NTD and LBD helices, are highlighted in stippled boxes on the blot (compared with D). The bottom panel shows a longer exposure for the LBD-A1 region (yellow) and the NTD-LBD linker (blue). See Figure S4A for a longer exposure of the blot. (D) TARP binding sites deduced from the peptide array in (C) are mapped onto the extracellular region of GluA2 (PDB: 3KG2). NTD interaction sites are denoted in deep red (strong interaction) and light pink (weaker interaction; see graded bar below), with alpha helices contacted by γ-2 denoted by (D), (F), and (H). LBD interaction sites are highlighted in brown (strong interaction) and yellow (weaker interaction). The three core contact regions, A–C, are denoted. Region A spans the glutamate binding cleft (interaction sites A_1_ and A_2_); region B encompasses the LBD-TM linker 1, and region C corresponds to the flip-flop cassette (denoted with a stippled ellipsoid). See also Figures S3, S4, and S6 and Table S1.

To establish whether the NTD directly mediates interaction with TARPs, we first used immunoprecipitation (IP) to test if the NTD contributes to stabilizing the AMPAR-TARP complex. We transfected either GluA2i WT or GluA2i-ΔNTD into HEK293T cells stably expressing TARP γ-2, extracted proteins under mild detergent conditions (Nakagawa et al., 2006) and IPed GluA2i/γ-2 complexes with an anti-γ-2 antibody. As shown in Figure 4A, the fraction of GluA2i-ΔNTD associating with γ-2 was markedly reduced (lanes 3 + 4) when compared with GluA2i WT (lanes 1 + 2). The ratio of IPed GluA2i WT to GluA2i-ΔNTD was ∼3-fold (2.9 ± 0.5; n = 5), when normalized to the input. TARP expression between conditions was comparable (Figure 4A, lower panel), and a similar association pattern was evident in the reverse experiment, where γ-2 was IPed with GluA2 (Figure S3A). Conversely, IP of GluA2i Δ link with γ-2 was similar to GluA2i WT (Figure S3B) and was not enhanced as one may have expected from the functional data (Figure 1).

Reduced association with γ-2 in absence of the AMPAR NTD was also observed for GluA1 (data not shown) and for other GluA2 isoforms, namely unedited GluA2i-Q_607_ and for the alternatively spliced GluA2-flop variety (GluA2o-R_607_). While there was no obvious difference in TARP association between the WT isoforms, we noted an isoform-specific difference between the ΔNTD mutants (Figure 4A, lanes 3–8). Specifically, the Q to R switch at the channel pore reduced coIP by ∼2-fold (2.2 ± 0.6, n = 5; lanes 4 versus 6) and the flop Δ-NTD mutant precipitated ∼3-fold (2.9 ± 0.3, n = 5) less efficiently than its flip counterpart (lanes 4 versus 8). These results imply that the NTD contributes to complex stability and that there are multiple regions on the AMPAR that mediate association with TARP auxiliary subunits.

### Delineating the TARP γ-2 Interaction Regions on GluA2

Thus far, our data suggested a reorganization of AMPARs when associating with TARPs. This prompted us to identify TARP binding sites on the receptor, which are currently unknown. We utilized peptide arrays, which provide semiquantitative maps of protein interaction regions (Katz et al., 2011; Shanks et al., 2014). We first probed an array of overlapping 15 amino acid peptides representing GluA2 with TARP γ-2 (Table S1). The array covered the NTD lower lobe, the NTD-LBD linker, the LBD and the transmembrane sector (schematic in Figure 4B). TARP γ-2 binding was revealed with an anti-γ-2 antibody (Supplemental Experimental Procedures).

As shown in Figures 4C, 4D, and S4, TARP interaction sites mapped to the LBD, the transmembrane region and indeed included the NTD. Interestingly, the NTD linker region was devoid of γ-2 binding. Even GluA2 peptides mimicking glycosylation (with GlcNAc-β[1-4]-GlcNAc) at the two N-glycosylation sites, N385 and N392, were negative (data not shown), suggesting that the linker is not directly contacted by the TARP but facilitates a specific orientation of the NTD (which is altered in Δ link). A similar pattern was observed with GluA3 where the NTD core that precedes the linker interacted with γ-2, whereas the linker itself was mostly devoid of signal (Figure S4A). Below we give a more detailed description of the γ-2 contact points on GluA2.

#### Regions of the NTD that Interact with γ-2

TARP contact regions mapped to various points on the NTD (Figures 4C, 4D, and S4). These included the front helices F and H, which have previously been implicated in NTD dynamics; they exhibit structural heterogeneity (Sukumaran et al., 2011) and undergo fluctuations when measured at a single-molecule level (Jensen et al., 2011) and in molecular dynamics simulations (Dutta et al., 2012). Of note, these helices also form an interface between NTD dimers (Jin et al., 2009; Sobolevsky et al., 2009), which may be disrupted by TARP association in the AMPAR tetramer (orange region in Figure S4B). Interaction sites also mapped to the side (close to helix H), the back of the NTD (along helix D), and across the NTD “floor” (Figures 4D, S4B, and S4C).

#### Regions of Interaction on the GluA2 LBD and the Transmembrane Sector

The LBD has been suggested as a key TARP modulatory target (Kato et al., 2010; Tomita et al., 2006, 2007). Our identification of TARP contact points on strategic regulatory sites on the LBD offers an explanation for these observations. These included the upper and lower “lip” of the LBD clamshell (regions A_1_, A_2_ in Figures 4C and 4D), the LBD-TMD linker region (region B), and the alternatively spliced flip/flop cassette (region C; Figures 4D and S4D) (Sommer et al., 1990).

TARP interaction with regions A and B suggests how TARP binding could modulate AMPAR gating kinetics and agonist efficacy. Region A stretches across both lobes of the LBD clamshell, extending from beta strand 2 in the upper lobe down to helix H in the lower lobe (Figures 4D and S4D) and is thus ideally positioned to affect LBD clamshell motions associated with gating. Region B encompasses LBD-TMD linkers, which translate LBD motions into channel opening. Strong signals were apparent in the LBD-TM1 linker but not in LBD-TM4. The TM3 linker, which connects the LBD to the channel gate, is positioned in the “interior” of GluA2 (Sobolevsky et al., 2009) and may be less accessible. The transmembrane sector, which exhibits prominent swelling in TARP-associated AMPARs (Nakagawa et al., 2005), also showed signs of interaction. However, this region also exhibited nonspecific antibody binding, so we cannot make any specific assignment at present (Figure 4C). Similarly, strong background signals were observed along LBD helices F and G.

Region C encompasses the flip/flop cassette (helices J and K), which also contributed to complex stability in our coIP experiments (Figure 4A). These contacts likely account for the flip/flop differences in TARP modulation (Kott et al., 2007; Turetsky et al., 2005) and the altered specificity of AMPAR modulators in the presence of TARPs (Tomita et al., 2006). As is apparent in Figure 4D, this region follows a continuous path toward the interaction patch on the back of the NTD (NTD helix D; Figure 4D, right), which might tether the LBD to the NTD via the TARP. Taken together, these results provide a glimpse into the γ-2 contact points and reveal the functionally critical regions of the AMPAR interacting with γ-2.

### Delineating GluA2 Binding Sites on TARPs γ-2 and γ-8

**Figure 5:**
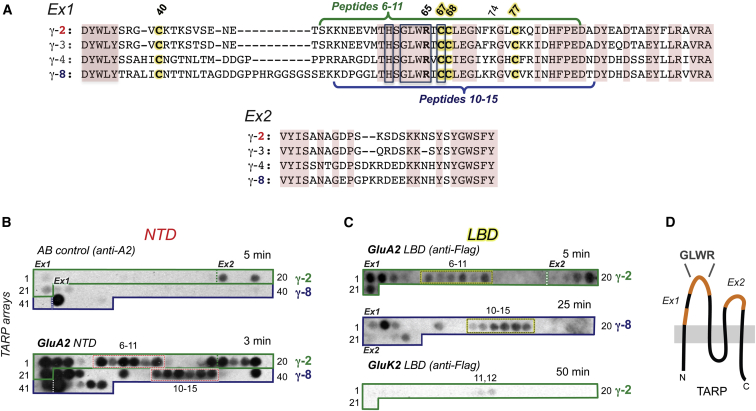
Mapping TARP γ-2 and TARP γ-8 Residues Involved in AMPAR Interaction (A) Alignment of rat type 1a (γ-2, γ-3) and type 1b (γ-4, γ-8) TARP extracellular loops, Ex1 and Ex2. Conserved residues are shaded brown, and residues highly conserved throughout the Cacng family (γ-1 to γ-8) are boxed in gray. The four cysteines are highlighted in yellow. Curly brackets above the γ-2 (green) and below γ-8 alignment (blue) indicate regions in the center of Ex1 interacting with the AMPAR extracellular domains (NTD and LBD). (B) TARP array encompassing the Ex1 and Ex2 segments of γ-2 (green box) and γ-8 (blue box) probed with the NTD. (Upper panel) Nonspecific signal, resulting from direct anti-GluA2 antibody binding to the membrane (AB control). The Ex1 and Ex2 regions for both TARPs are denoted (dashed line). (Lower panel) the same membrane was exposed to the rat GluA2 NTD followed by probing with anti-GluA2 AB. The membrane exposure time is as indicated. See Table S2 for peptide sequences. (C) γ-2 and γ-8 arrays probed with GluA2 LBD and GluK2 LBD. The negative controls with anti-FLAG AB did not show any signal and thus are not shown. Membranes were then incubated with FLAG-tagged GluA2 LBD or GluK2 LBD and probed with anti-FLAG AB. (Upper panel) GluA2 LBD interaction with γ-2 (same peptides as in Figure 5B, green box). (Central panel) GluA2 LBD interaction with γ-8 (same peptides as in B, blue box). (Lower panel) the γ-2 membrane previously probed with GluA2 LBD (top panel) was regenerated and probed with the FLAG-tagged GluK2 LBD, which produced no clear binding. Regeneration of this membrane resulted in a clear binding pattern when reprobed with the GluA2 LBD that matched the one shown in (C, top). (D) Schematic representation of TARP structure with the GluA2 NTD and LBD interacting parts of the Ex1 and Ex2 loops highlighted in orange and the highly conserved GLWR motif indicated.

Next, we used an array of TARP peptides to identify TARP residues that contact the AMPAR (Table S2). We examined loops Ex1 and Ex2 (Figure 5A) in both type 1a (γ-2) and type 1b (γ-8) TARPs and mapped sites contacted by the NTD and LBD. Extensive contacts were indeed apparent on both γ-2 and γ-8 when we probed the array with the NTD (Figure 5B, lower panel). Only background signals were obtained when we omitted the NTD and tested the membrane with the antibody alone (upper panel). In addition to identifying NTD binding sites on Ex1, we also detected signals on the smaller Ex2 loop, which is only ∼30 residues in length and thus not expected to protrude far above the plane of the plasma membrane. Moreover, in Ex1, the membrane-proximal N and C termini exhibited regions of NTD interaction. In vivo, these interactions would require substantial reconfigurations of the receptor, with the NTD reaching down toward the membrane (Figure S6B); NTD reconfigurations have been observed in low-resolution structures of native AMPARs (Nakagawa et al., 2005). A prominent interaction region was also present in the center of Ex1, surrounding the highly conserved GLWRxC_67_ motif present throughout the vertebrate Cacng family (γ-1 to γ-8) (Figures 5A, 5B, and 5D).

The same peptides were also probed with a Flag-tagged GluA2 LBD. Surprisingly, the LBD interaction sites on the TARPs γ-2 and γ-8 largely overlapped with those for the NTD. There were two noticeable differences: (1) the relative weight of signals across the Ex1 tip region, surrounding the double cysteine motif, varied between the LBD and NTD and (2) within Ex1, LBD binding to the N-terminal end of the loop was greater, whereas interaction with the tip region of the loop was reduced (Figure 5C). This indicates that the LBD interacts strongly with the membrane proximal region of Ex1, whereas the NTD binds more intimately to the Ex1 segment that surrounds the double cysteine motif (CC67, CC68).

The similarity between the NTD and LBD binding pattern prompted us to probe the specificity of this interaction further. As the related kainate receptors do not interact with TARPs (Chen et al., 2003), we probed the γ-2 array with a Flag-tagged GluK2 LBD (the GluA2 and GluK2 LBDs share only ∼50% sequence identity) and did not observe clear binding (Figure 5C, bottom panel), suggesting that the observed AMPAR/γ-2 interaction profile is genuine. Together, these results corroborate an interaction of the NTD with type 1a and type 1b TARPs and reveal the sites on the TARPs involved in modulating AMPARs.

### Interaction Regions in the γ-2 Ex1 Loop Are Critical for TARP Function

**Figure 6:**
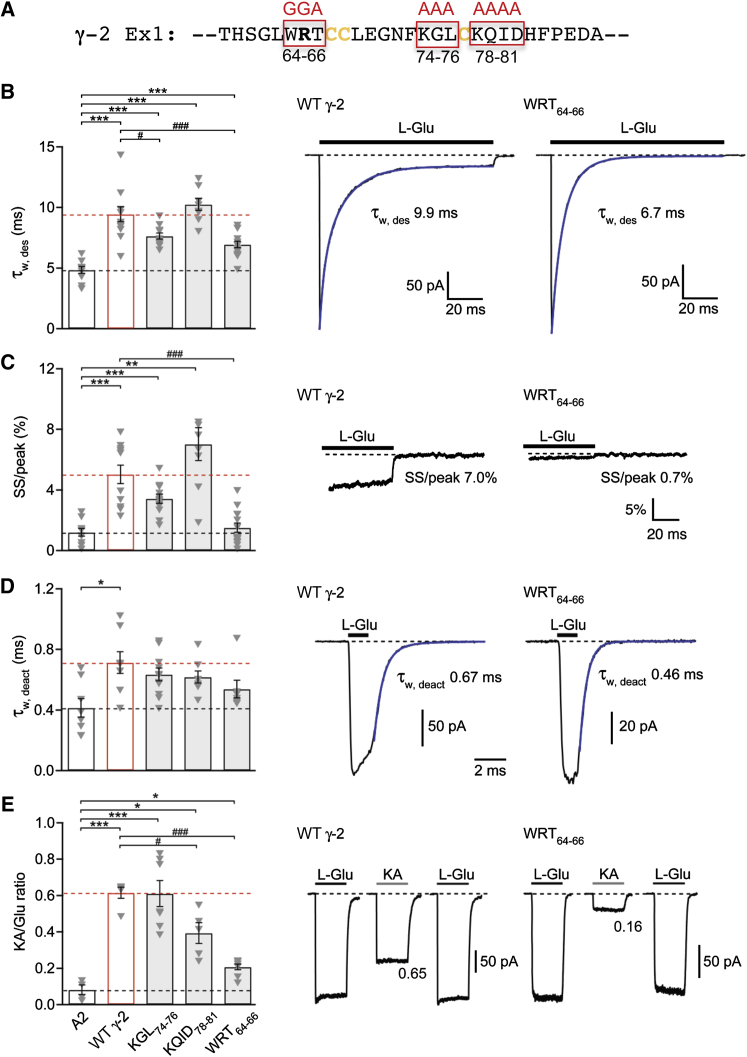
Effects of Ex1 Mutations in TARP γ-2 (A) Sequence of the γ-2 Ex1 region surrounding the highly conserved GLWRxC_67_ motif. Boxed regions in red identify the amino acid triplets and quadruplet mutated in these experiments. (B) Pooled data (mean ± SEM) showing the effects of Ex1 mutations in γ-2 on desensitization (τ_w,des_) of GluA2. Following one-way ANOVA (*F*_4, 23.27_ = 28.89, p = 1.01 × 10^−8^), pairwise comparisons showed that γ-2 WT (n = 12) and all three γ-2 mutants (KGL_74–76_, KQID_78–81_, and WRT_64–66_; n = 12, 8, and 14, respectively) slowed τ_w,des_ compared with GluA2 alone (n = 10) (^∗∗∗^p < 0.001) and that the effects of KGL_74–76_ and WRT_64–66_ were less than those of γ-2 WT (^#^p < 0.05, ^###^p < 0.001) (Welch t tests). To the right are representative currents evoked by 10 mM L-glutamate (−60 mV) in patches from cells expressing GluA2/γ-2 WT and GluA2/γ-2 WRT_64–66_. Also shown are the individual τ_w,des_ values determined from double exponential fits (blue). (C) Pooled data showing the effects of mutations in γ-2 on the steady-state/peak ratio (SS/peak). Presentation, analysis, and n numbers as in B (*F*_4, 22.91_ = 18.26, p = 7.27 × 10^−7^; ^∗∗^p < 0.01 and ^∗∗∗^p < 0.001 compared with GluA2 alone and ^###^p < 0.001 compared with γ-2 WT). To the right are representative records from GluA2/γ-2 WT and GluA2/γ-2 WRT_64–66_ illustrating the current remaining at the end of the 100 ms applications of L-glutamate (10 mM) and the calculated SS/peak ratio. (D) Pooled data (±SEM) showing the effects of mutations in γ-2 on GluA2 deactivation (τ_w,deact_; 1 ms, 10 mM L-glutamate, −60 mV). Following one-way ANOVA (*F*_4, 17.47_ = 3.06, p = 0.044), pairwise comparisons showed that only for γ-2 WT (n = 8) was τ _w,deact_ slowed compared with GluA2 alone (n = 8) (^∗^p < 0.05). None of the mutants (KGL_74–76_, KQID_78–81_, and WRT_64–66_; n = 11, 8, and 7, respectively) differed from GluA2 alone or GluA2/γ-2 WT. To the right are representative currents in patches taken from cells expressing GluA2/γ-2 WT and GluA2/γ-2 WRT_64–66_. Also shown are the individual τ_w,deact_ values determined from double exponential fits (blue). (E) Pooled data (± SEM) showing the effects of mutations in γ-2 on the KA/Glu ratio. Following one-way ANOVA (*F*_4, 9.85_ = 44.8, p = 2.72 × 10^−6^), pairwise comparisons showed that γ-2 WT (n = 5) and all three γ-2 mutants (KGL_74–76_, KQID_78–81_, and WRT_64–66_; n = 7, 5, and 8, respectively) increased kainate efficacy (KA/Glu ratio) compared with that seen with GluA2 alone (n = 4) (^∗^p < 0.05, ^∗∗∗^p < 0.001) and that KQID_78–81_ and WRT_64–66_ had less effect than γ-2 WT (^#^p < 0.05, ^###^p < 0.001) (Welch t tests). To the right are representative currents evoked by L-glutamate and kainate (both 500 μM, in the presence of 100 μM cyclothiazide) in patches from cells expressing GluA2/γ-2 WT and GluA2/γ-2 WRT_64–66_. Also shown are the KA/Glu ratios for these representative records. See also Figure S5.

To test the functional relevance of the identified binding region (Figure 5D), we mutated the γ-2 Ex1 segment contacting the AMPAR and examined the effects on GluA2i currents. As shown in Figure 6A (top), triple and quadruple mutations were introduced into the tip region of Ex1. We tested the ability of TARP mutants to modulate channel kinetics, kainate efficacy (Figure 6), inward rectification, and channel conductance (Figure S5) (Jackson et al., 2011; Soto et al., 2007). We found that although all three γ-2 mutants retained some TARP-like functions, various channel parameters were affected differently and to varying degrees. For example, with the KGL_74–76_ mutation, desensitization was faster than with γ-2 WT (Figure 6A), whereas kainate efficacy was unchanged relative to γ-2 WT (Figure 6D). On the other hand, GluA2i coexpressed with KQID_78–81_ exhibited significantly lower kainate efficacy than did receptors expressed with γ-2 WT, while desensitization kinetics were comparable. The reduced kainate efficacy seen with KQID_78–81_ and WRT_64–66_ when compared with γ-2 WT suggests that these mutants disrupt TARP-AMPAR interactions in a way that might affect the degree of the LBD cleft closure, as this is known to determine the efficacy of partial agonists (Jin et al., 2003).

All γ-2 Ex1 mutants increased the weighted-mean channel conductance of GluA2i to the same extent as γ-2 WT (Figure S5). By contrast, the WRT_64–66_ mutant produced less relief of polyamine block than did γ-2 WT (Figure S5), where the effect of the mutation was evident only at positive potentials (data not shown). These observations suggest that regions of the TARP distinct from Ex1, such as TM2 may play a role in modulating AMPAR properties, particularly those related to ion permeation, which likely result from interactions close to the channel pore.

Among the Ex1 mutants examined here, the WRT_64–66_ mutation had the most profound impact on a variety of functional properties (Figures 6 and S5). WRT_64–66_ was expressed at lower levels (∼50%) than γ-2 WT; however, this mutant was targeted to the cell surface and biotinylation experiments revealed that the proportion of surface-expressed versus internal WRT_64–66_ was comparable to the other γ-2 mutants (data not shown). In summary, our results identify functional hotspots in the γ-2 Ex1 loop and imply the existence of regions on the TARP that selectively influence different aspects of the AMPAR gating spectrum.

## Discussion

In this study, we report a role for the NTD (and the NTD-LBD linker) in AMPAR modulation by TARPs. We show that the NTD has the capacity to interact with TARPs and that selective shortening of the NTD linker can potentiate the modulatory function of TARPs in a TARP-selective fashion. Using peptide arrays, we identify NTD and LBD segments contacting γ-2 (Figure S6A), shedding light on the mechanisms underlying TARP modulation. In addition, we characterize contact points of the NTD and LBD on TARPs γ-2 and γ-8 and identify functional hotspots in the γ-2 interaction region. Our results imply that AMPARs are highly dynamic and may substantially reconfigure when interacting with auxiliary subunits (Figure S6B). We hypothesize that the flexible, modular organization of the AMPAR extracellular region permits selective interaction with other synaptic components, which may impact allosteric regulation of AMPARs.

### The AMPAR Extracellular Region Is Flexible

The extracellular region of AMPA (and kainate) receptors constitutes ∼80% of the mass of the receptor. The LBD layer is wedged between the ion channel and the NTD and is connected to both domains via peptide linkers. This flexible attachment, together with weak contacts within the LBD layer, is intimately linked to receptor gating, which requires substantial reconfigurations. In addition to the well-studied intradimer rearrangements associated with AMPAR desensitization (Armstrong et al., 2006; Sun et al., 2002), recent data from GluK2 kainate receptors reveal complete separation of the four LBDs upon desensitization (Schauder et al., 2013). This loose architecture permits large rearrangements that are required for AMPAR gating on the millisecond time scale (Plested and Mayer, 2009).

Subunit interactions within the distal NTD layer are substantially tighter as NTD dimers exhibit low nanomolar to low micromolar affinities (Herguedas et al., 2013; Rossmann et al., 2011; Zhao et al., 2012). These dimers associate as tetramers through a relatively small interface (Clayton et al., 2009; Jin et al., 2009; Kumar et al., 2009; Sobolevsky et al., 2009), which appears to be contacted by TARPs (Figure S4B). TARP association could therefore impact the organization of the distal layer, perhaps in a state-dependent fashion. Our peptide array data also imply that interdomain interactions between the NTD and LBD are altered by TARPs, which may underlie changes in desensitization rates observed in response to linker truncation.

As TARPs are not expected to protrude far beyond the plane of the membrane (Suzuki et al., 2014), a direct contact between TARPs and the NTD would require substantial rearrangements of the receptor. Structural data lend some support to this hypothesis (Nakagawa et al., 2005). Since interactions within a membrane-embedded receptor complex likely differ from those in a peptide array probed with isolated (and therefore unconstrained) receptor domains, not all interactions described here may occur at the same time and may also depend on the functional state of the receptor. Related to this, TARP contacts may differ between the two nonequvivalent AMPAR subunits pairs (*AC* vs. *BD*; Sobolevsky et al., 2009).

### TARPs Interact with Functionally Critical AMPAR Regions

Within the LBD, TARP contacts include a number of sites of functional importance. Interactions across the LBD cleft are well suited to affect kainate efficacy, the pharmacology of competitive antagonists, and the open-to-closed equilibrium of the clamshell (Cho et al., 2007; Milstein et al., 2007). Similarly, the LBD-TM1 linkers, which are contacted by γ-2, are involved in transmitting gating motions from the LBD to the ion channel and are thus well suited to shape gating. Curiously, TARPs contact the alternatively spliced flip/flop segment, which, according to our IP data, impacts the stability of the AMPAR/TARP complex. Our coIP results imply multiple regulatory sites on the receptor, the Q/R site in the pore, and the flip/flop cassette in the LBD, which in combination would determine AMPAR affinity for the TARP. This result highlights the strategic role of flip/flop splicing in AMPAR regulation (Coleman et al., 2006; Penn et al., 2012; Sommer et al., 1990). Somewhat unexpectedly, the GluA2i-ΔNTD mutant retained modulation by γ-2 to comparable levels as GluA2i WT (Figures S2C–S2E). Interestingly, earlier work showed that while GluA2i lacking the NTD was potentiated by γ-2, the GluA2o-ΔNTD mutant was not (Bedoukian et al., 2006). This may well be explained by the reduced TARP affinity seen with the flop variety in our coIPs and further indicates that multiple binding sites on the receptor (Figure S6A) contribute to elicit optimal TARP modulation.

Surprisingly, on the TARP itself, the GluA2 LBD and NTD contacted comparable regions in the extracellular loops; however, the relative weights of signal intensities were distinct. This pattern of interaction is unexpected and prevented us from selectively mutating residues within LBD versus NTD interaction regions. Structural data are required to resolve the precise organization of AMPAR TARP complex. Another curious feature of our results is the close similarity between γ-2 and γ-8 in their interactions with AMPAR. These two TARPs are of strikingly different length and share only ∼48% sequence similarity in their extracellular regions. As signals across Ex1 centered around the four cysteines, tertiary structural features of the loop that result from disulphide bonding (Suzuki et al., 2014) are likely to underlie this interaction. The functional consequence of the NTD-γ8 interaction remains to be established.

### Functional Implications of TARP-Induced AMPAR Rearrangements

A continuous path of TARP interaction, extending beyond the flip/flop region toward the back of the NTD, may permit “bridging” between the NTD and LBD. The resulting compact arrangement between these two domains may “incorporate” the otherwise loosely connected NTD layer into an allosteric unit with the LBD (Figure S6B). The AMPAR rearrangement, triggered by TARPs, reflects a capacity of the AMPAR for dynamic reorganization that might permit interactions with other synaptic components, such as cadherins and pentraxins (Saglietti et al., 2007; Sia et al., 2007), to impact postsynaptic response properties via the NTD.

The sequence diversity of the NTD, combined with the existence of multiple TARPs heterogeneously expressed in diverse neuronal populations, may provide further capacity for differential regulation of AMPAR subtypes. First, the NTD-TARP contact region described in this study may offer a target for the development of novel AMPAR-subtype selective drugs (Gill and Bredt, 2011). Second, AMPARs have been suggested to dissociate from TARPs upon activation by L-glutamate, prior to endocytosis (Tomita et al., 2004). Thus, AMPAR subunit combinations with different affinities for TARPs, mediated via the sequence-diverse NTD, could exhibit distinct endocytosis rates and lateral diffusion, influencing the dwell-time of AMPARs at postsynaptic sites (Bredt and Nicoll, 2003; Opazo et al., 2012). Therefore, the extracellular region of AMPARs might turn out to be a key element for regulating functional and structural plasticity at excitatory synapses.

## Experimental Procedures

Additional details are provided in the Supplemental Experimental Procedures.

### Protein Production

His-tagged γ-2 was produced in insect cells using a P1 baculovirus stock following a purification protocol provided by T. Nakagawa. High-titer viral stocks were obtained following the Bac-N-Blue protocol (Invitrogen). Protein was solubilized with decyl-maltoside and purified by Cobalt-affinity chromatography. The GluA2 NTD was purified from stably transfected GntI^−^ HEK293S cells (Rossmann et al., 2011). A FLAG-tag was introduced at the GluA2i LBD C terminus (R/Flip; (Armstrong and Gouaux, 2000; Greger et al., 2006) and the GluK2 LBD (provided by M. Mayer) and cloned into a pET22b(+) plasmid containing an N-terminal His_8_ tag and a thrombin cleavage site. Proteins were produced in Origami B (DE3) cells and purified on a HisTrap HP column followed by thrombin cleavage and gel filtration.

### Peptide Arrays

The interaction between GluA2 and TARPs γ-2 and γ-8 was mapped with peptide arrays synthesized by SPOT synthesis (PepSpots from JPT Peptide Tech.). AMPAR and TARP arrays contained 15-mer overlapping peptides shifted by four residues (Tables S1 and S2). The GluA2 array was probed with full-length γ-2, whereas the TARP γ-2 and γ-8 arrays were probed with GluA2 NTD, GluA2 LBD, or GluK2 LBD, following the manufacturer’s protocol. Prior to exposing the arrays to specific protein probes, the membranes were incubated with antibodies only to determine nonspecific binding. Membranes were blocked with 5% BSA and incubated with primary antibodies: anti-FLAG (monoclonal; Sigma-Aldrich), anti-Stargazin (polyclonal; Millipore), or anti-GluA2 (polyclonal; Alomone). After incubation with HRP-coupled secondary antibodies (Pierce), membranes were developed with enhanced chemiluminescence and images were captured electronically with a ChemiDoc MP Imaging System (Biorad) or on an X-ray film.

### Electrophysiology

Voltage-clamp recordings of rat GluA2i (flip, R/G-edited, Q/R-unedited) or GluA3i (flip, R/G-edited and containing the R463G point mutation for increased surface expression; Coleman et al., 2010) were performed as described previously (Rossmann et al., 2011; Soto et al., 2007). Briefly, outside-out patches were pulled from HEK293(T) cells transfected with rat GluA2i or GluA3i, and current responses to rapid application of 10 mM L-glutamate via a θ tube were recorded. Where indicated, TARPs were coexpressed, either transiently cotransfected or using a cell line stably expressing γ-2 or γ-8. The kinetics of the receptor desensitization, deactivation, and recovery from desensitization were analyzed by fitting currents with single- or double-exponential functions. In addition, steady state-to-peak ratio, relative kainate efficacy, rectification, and single channel properties (by nonstationary fluctuation analysis) were assessed.

### Statistics

Summary data are presented as the mean ± SEM from *n* patches. Comparisons involving two data sets only were performed using a two-sided Welch two-sample t test. All analyses involving data from three or more groups were performed using one- or two-way analysis of variance (Welch heteroscedastic F test) followed by pairwise comparisons using two-sided Welch two-sample t tests (with Holm’s sequential Bonferroni correction for multiple comparisons). Differences were considered significant at p < 0.05. Statistical tests were performed using Prism 4.0/6.0 (GraphPad Software) or R (v.3.0.2, The R Foundation for Statistical Computing, http://www.r-project.org/) and RStudio (v.0.98.313, RStudio).

## Author Contributions

I.H.G., M.F., and S.G.C.-C. designed and interpreted experiments and wrote the manuscript. I.H.G., O.C., B.H., and K.K. performed experiments and contributed to design of experiments and writing. O.C., I.H.G., B.H., K.K., and M.F. analyzed data.

## Supplemental Information

Document S1. Supplemental Experimental Procedures, Figures S1–S6, and Tables S1 and S2

Document S2. Article plus Supplemental Information

## Note Added in Proof

While this paper was under review, the two following structural studies provided additional evidence for the structural dynamics of the AMPAR NTD layer:

Dürr, K.L., Chen, L., Stein, R.A., De Zorzi, R., Folea, I.M., Walz, T., Mchaourab, H.S., and Gouaux, E. (2014). Structure and dynamics of AMPA receptor GluA2 in resting, pre-open, and desensitized states. Cell *158*, 778–792.

Meyerson, J.R., Kumar, J., Chittori, S., Rao, P., Pierson, J., Bartesaghi, A., Mayer, M.L., and Subramaniam, S. (2014). Structural mechanism of glutamate receptor activation and desensitization. Nature. http://dx.doi.org/10.1038/nature13603.
