# Supplementary material for: Mapping the Interaction Sites between AMPA Receptors and TARPs Reveals a Role for the Receptor N-Terminal Domain in Channel Gating
Source: Cell Rep. 2014 Oct 16;9(2):728–40. doi: 10.1016/j.celrep.2014.09.029 (PMC4405707; doi:10.1016/j.celrep.2014.09.029)
Supplement: Document S1. Supplemental Experimental Procedures, Figures S1–S6, and Tables S1 and S2 [file mmc1.pdf]

Cell Reports, Volume *9*

Supplemental Information

Mapping the Interaction Sites between AMPA Receptors and TARPs Reveals a Role for the Receptor N-Terminal Domain in Channel Gating

Ondrej Cais, Beatriz Herguedas, Karolina Krol, Stuart G. Cull-Candy, Mark Farrant, and Ingo H. Greger

Supplemental Information

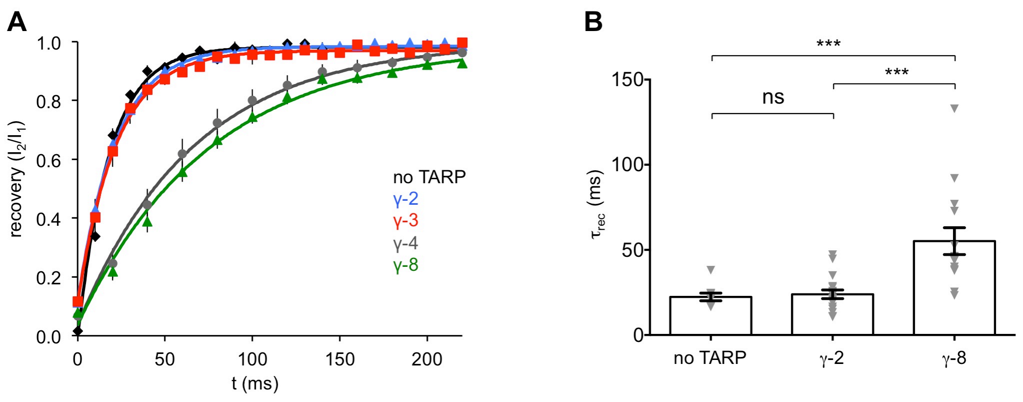

Figure S1. Differential modulation of GluA2 and GluA3 recovery from desensitization by Type-1a and -1b TARPs, related to Figure 2.

A. Graphical summary of recovery from desensitization of GluA2 wt expressed with different TARPs. Relative currents at individual time points are shown ± SEM (error bars masked by the symbols in most cases). The solid lines are monoexponential fits of the averages, giving similar time constants of 18.1 ms, 21.8 ms and 22.2 ms for GluA2 alone (n = 11), GluA2 with γ-2 (n = 17) and GluA2 with γ-3 (n = 6), respectively. Much slower recovery was seen for GluA2 with γ-4 (66.5 ms; n = 8) and GluA2 with γ-8 (75.6 ms; n = 7).

B. Pooled data (shown ± SEM) for the time constant of recovery from desensitization for GluA3i (R463G) expressed alone or with TARPs γ-2 or γ-8 (n = 9, 17 and 14, respectively) (*** $P < 0.001$; two-sided Welch two-sample t-tests with Holm's sequential Bonferroni correction for multiple comparisons).

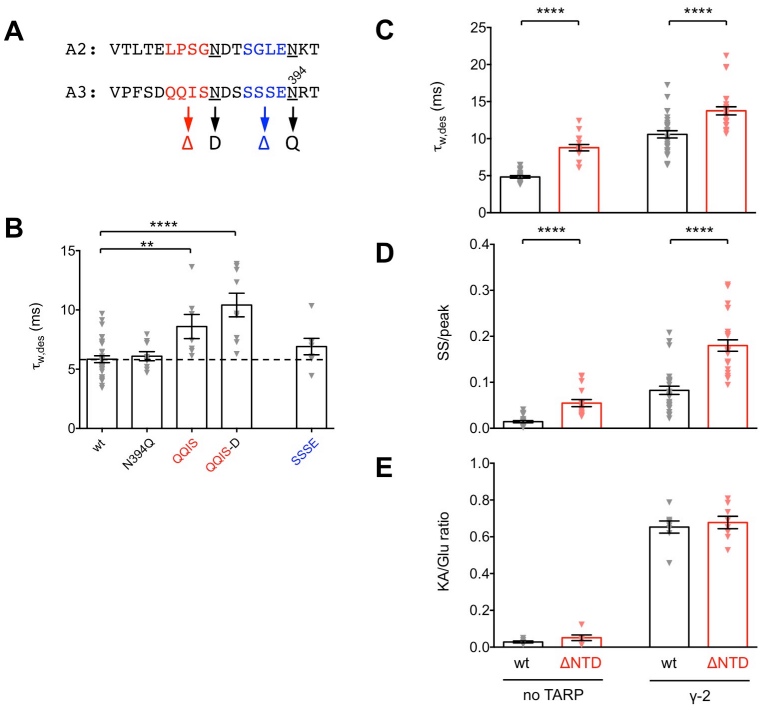

Figure S2. NTD-LBD linker mutations affect desensitization of GluA3, the effect of γ-2 TARP on GluA2 gating is not eliminated in the absence of NTD, related to Figure 3.

A. Sequence alignment of the NTD-LBD linkers of GluA2 and GluA3, with the deleted/mutated regions highlighted (GluA2 mutants described in Figure 3).

B. Pooled data (± SEM) showing the effect of NTD-LBD linker mutations on desensitization kinetics of GluA3 in the presence of TARP γ-2. QQIS denotes deletion of these four amino acids, QQIS-D combines this with the N387D point mutation, while SSSE is an alternative four-amino acid deletion. Values were compared using one-way ANOVA. Pairwise comparisons showed that $\tau_{w,des}$ was significantly slower for QQIS (n = 7) and QQIS-D (n = 9) compared to wt (n =31) (** $P < 0.01$, *** $P < 0.001$) (two-sided Welch two-sample t tests with Holm's sequential

Bonferroni correction for multiple comparisons). There was no significant effect of the glycosylation mutation N394Q (n = 9) or the alternative deletion mutant, SSSE (n = 7).

C. Pooled data (± SEM) showing the effect of NTD deletion on desensitization kinetics of GluA2i. $\tau_{w,des}$ was significantly increased both in the absence (n = 23 and 16 for GluA2 wt and ΔNTD, respectively) and presence (n = 29 and 25) of TARP γ-2 (**** $P < 0.0001$; two-sided Welch two-sample t test). Two-way ANOVA showed no significant interaction between NTD and γ-2 presence ($F_{1,\ 89} = 0.69$, $P = 0.4084$).

D. Pooled data for the steady state-to-peak ratio, presented and analysed as in C. SS/Peak was significantly higher for the ΔNTD both in the absence (n = 21 and 16 for GluA2 wt and ΔNTD, respectively) and presence (n = 28 and 25) of γ-2 (**** $P < 0.0001$; Welch t test). Here, two-way ANOVA showed significant interaction between NTD and γ-2 presence ($F_{1,\ 86} = 10.41$, $P = 0.0018$).

E. Pooled data for the kainate efficacy assay, comparing amplitude of responses to kainate and L-glutamate (KA/Glu ratio; both 500 μM, in the presence of 100 μM cyclothiazide), presented and analysed as in C. No difference between GluA2 wt and ΔNTD either in absence (P = 0.2064; n = 6 for both) or presence (P = 0.6105; n = 8 for both) of γ-2. Two-way ANOVA showed no significant interaction between NTD and γ-2 presence ($F_{1,\ 24} = 9.733 \times 10^{-4}$, $P = 0.9754$).

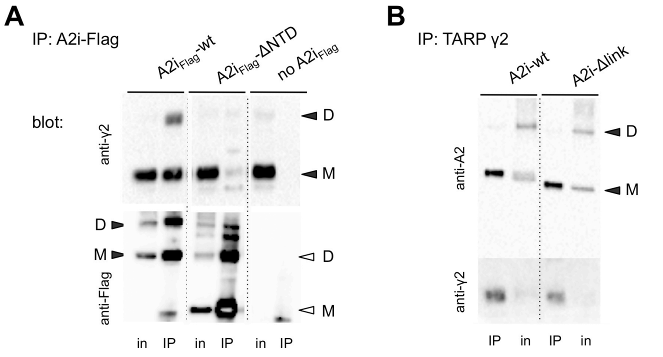

Figure S3. Immunoprecipitation (IP) of TARP γ-2 with GluA2i mutants, related to Figure 4.

A. Representative IP of γ-2 with GluA2i-wt (left) and GluA2i-ΔNTD (right). The blot was probed with polyclonal anti γ-2 (top panel) and anti-Flag antibody to detect GluA2i. Substantially less γ-2 precipitated with the ΔNTD mutant. Both wt and ΔNTD GluA2i migrated as monomer (M) and dimer (D), denoted by arrowheads. Note that inputs were comparable, while amounts of IPed TARP γ-2 varied between conditions.

B. Representative IP of GluA2i-wt (left) and GluA2i-Δlink (right) with γ-2. There was no visible difference between GluA2i-wt and the Δlink mutant with regard to TARP γ-2 association. The lower panel show that comparable amounts of γ-2 were present in the two reactions.

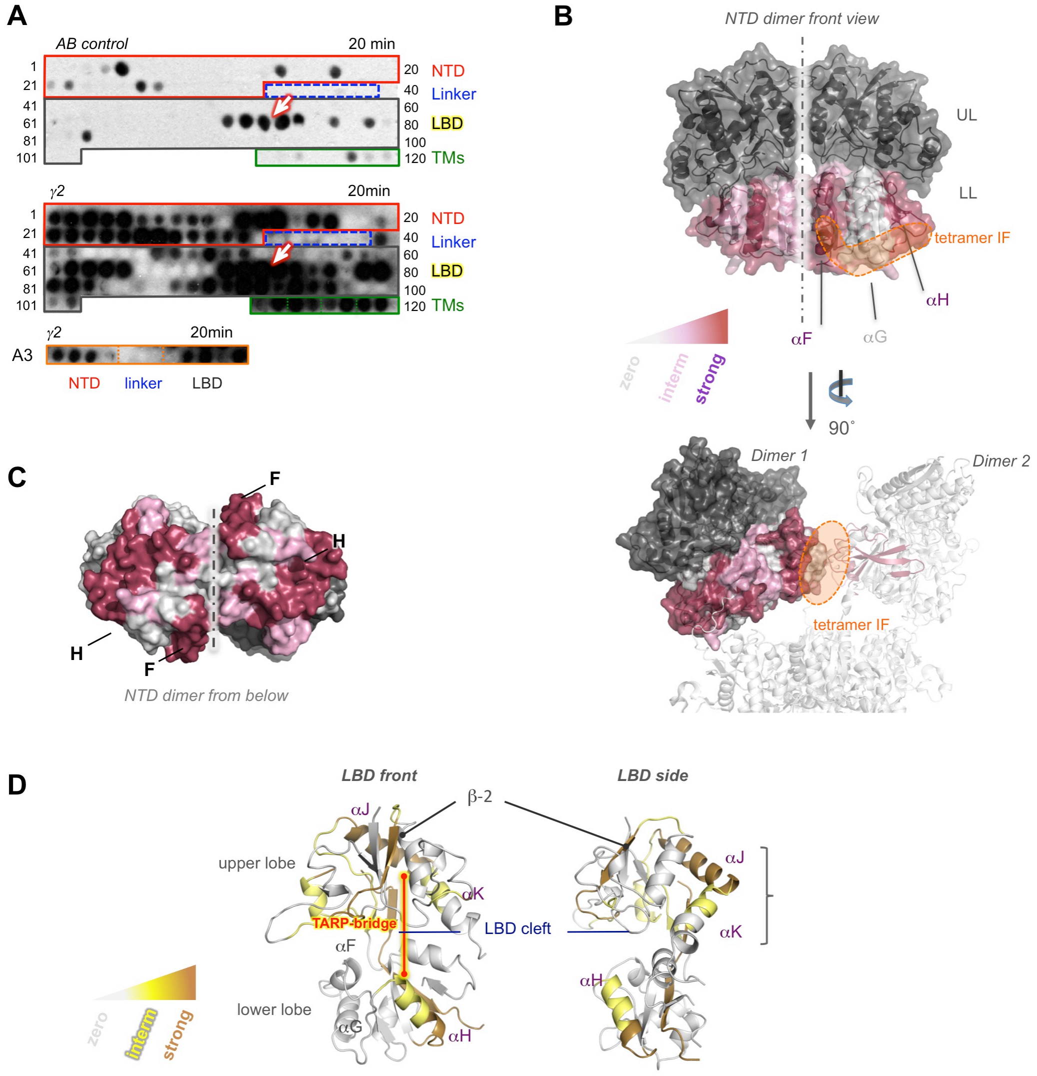

Figure S4. Mapping the TARP γ-2 contact region on GluA2, related to Figure 4.

A. Regions of GluA2 and GluA3 AMPARs binding to TARP γ-2. Array membrane containing GluA2 and -A3 peptides was probed with anti-γ-2 antibody only (AB control, <u>top panel</u>), or incubated with purified γ-2 protein before AB-probing (<u>lower panel</u>) and exposed for 20 minutes. Note the absence of γ-2 signal across the GluA2 linker region (blue box). The NTD-binding region is boxed in red, the LBD region in grey and the TM segments in green. Peptide numbers are indicated on the sides. Lower panel shows the binding of TARP γ-2 to the GluA3 region

flanking the NTD-LBD linker. Binding is observed for the GluA3 NTD and LBD but not for the linker peptides. Non-specific signals are indicated by the tilted white arrow.

B. TARP γ-2 interaction sites on the GluA2 NTD. Contact sites are shown in deep-red (strong interaction) or light pink (weaker interaction). The orange footprint outlines the tetrameric interface that is formed between two NTD dimers in crystal structures. The bottom panel shows the GluA2 NTD dimer superposed on the full-length structure (PDB: 3KG2), with the tetrameric interface (between the NTD dimers) coloured in orange. The stippled line denotes the NTD dimer interface. Upper lobe (UL), lower lobe (LL).

C. TARP γ-2 interactions with the NTD "floor" (a potential NTD-LBD interface). The relative strength of binding is indicated by the same colour gradient as in panel B. The positions of helices H and F are indicated. The stippled line denotes the dimer interface.

D. TARP γ-2 interactions with the LBD. Structural elements involved in the TARP-interaction are shown in brown (strong interaction) and yellow (weaker interaction) in front view (left) and side view (right). Contacts spanning the ligand-binding cleft region are indicated by the red line ('TARP bridge') and involve β-2 in the LBD upper lobe and helix H in the lower lobe. LBD helices J and K are the target of alternative splicing (flip-flop).

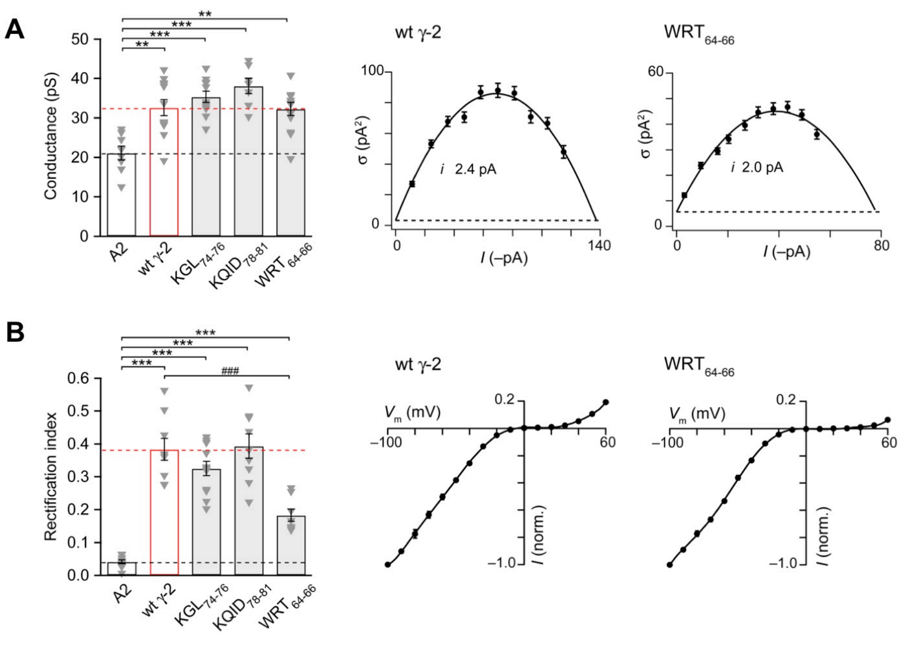

Figure S5. Effects of Ex1 mutations in TARP γ-2 on GluA2 channel conductance and rectification, related to Figure 6.

A. Pooled data (mean ± SEM) showing the effects of Ex1 mutations in γ-2 on weighted mean single-channel conductance from NSFA (10 mM L-glutamate, 100 ms, –60 mV). Values were compared using one-way ANOVA (Welch heteroscedastic *F* test: $F_{4, 21.02} = 12.26$, $P = 2.67 \times 10^{-5}$). Pairwise comparisons showed that γ-2 wt (n = 12) and all three γ-2 mutants (KGL_{74-76}, $KQID_{78-81}$ and WRT_{64-66}; n = 11, 7 and 13, respectively) increased channel conductance compared to that of GluA2 alone (n = 8) (** $P < 0.01$, *** $P < 0.001$) (two-sided Welch two-sample *t*-tests with Holm's sequential Bonferroni correction for multiple comparisons). For each of the mutants, the conductance was not statistically different from that seen with γ-2 wt. Shown to the right are representative individual current-variance plots for GluA2/ γ-2 wt and

GluA2/ γ-2 WRT_{64-66}. The single channel current (*i*) is derived from the fitted curve (solid line); the dashed line indicates the background variance.

B. Pooled data showing the effects of mutations in γ-2 on the rectification index (RI) of GluA2. Presentation and analysis as in A ($F_{4, 18.16}$ = 79.31, $P = 3.02 \times 10^{-11}$; *** $P < 0.001$ compared to GluA2 alone and $^{###}P < 0.001$ compared to γ-2 wt). Shown to the right are representative normalized *I-V* plots from patches expressing GluA2/ γ-2 wt and GluA2/ γ-2 WRT_{64-66}. Fitted curves are 8th order polynomials. Note that WRT_{64-66} produced less change in RI than did γ-2 wt. This change in RI appeared to reflect changes in spermine permeation, as the effects of WRT_{64-66} were evident in the outward limb of the *I-V* but not apparent at negative voltages, where the conductance-voltage plots were essentially indistinguishable (data not shown).

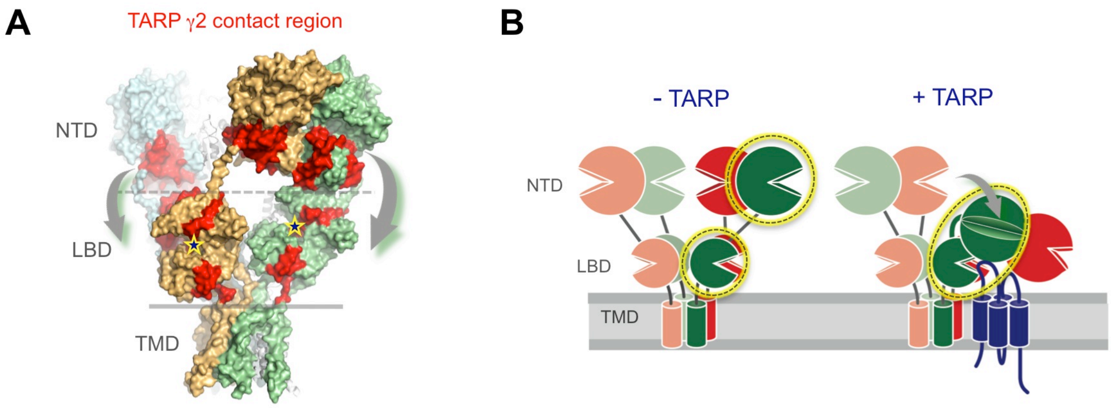

Figure S6. TARP contacts with the NTD may require AMPAR reorganization, related to Figure 4.

A. TARP γ-2 contact region mapped onto the GluA2 tetramer (PDB: 3KG2). Binding sites on the NTD, LBD and LBD-TMD linkers are indicated in red. The position where L-glutamate binds is indicated with stars. The potential movement NTDs need to undergo to contact the TARPs is denoted with grey arrows.

B. Model outlining the reorganization in the AMPAR extracellular region that may accompany TARP interaction. Left panel: Without TARP, the NTD and LBD are loosely connected. Any potential allosteric signal emanating from the NTD is not transmitted to the LBD (and the receptor), i.e. the NTD is functionally isolated (yellow circles). Right panel: The TARP 'bridges' NTD and LBD. This requires substantial receptor reconfiguration mediated by the NTD-LBD linkers. As a result the NTD and LBD are functionally connected (yellow ellipsoid).

GluA2 NTD

1	DLKGALLSLIEYYQW
2	ALLSLIEYYQWDKFA
3	LIEYYQWDKFAYLYD
4	YQWDKFAYLYDSDRG
5	KFAYLYDSDRGLSTL
6	LYDSDRGLSTLQAVL
7	RGLSTLQAVLDSAAE
8	AVLDSAAEKKWQVTA
9	AEKKWQVTAINVGNI
10	WQVTAINVGNINNDK
11	AINVGNINNDKKDET
12	GNINNDKKDETYRSL
13	KDETYRSLFQDLELK
14	YRSLFQDLELKKERR
15	DLELKKERRVILDCE
16	KKERRVILDCERDKV
17	RVILDCERDKVNDIV
18	DCERDKVNDIVDQVI
19	VNDIVDQVITIGKHV
20	DQVITIGKHVKGYHY
21	IGKHVKGYHYIIANL
22	VKGYHYIIANLGFTD
23	HYIIANLGFTDGDLL
24	ANLGFTDGDLLKIQF
25	FTDGDLLKIQFGGAN
26	LLKIQFGGANVSGFQ
27	LKIQFGGANVSGFQI
28	TINIMELKTNGPRKI
29	INIMELKTNGPRKIG
30	IMELKTNGPRKIGYW
31	KTNGPRKIGYWSEVD
32	RKIGYWSEVDKMVVT

GluA2 Linker

33	YWSEVDKMVVTLTEL
34	VDKMVVTLTELPSGN
35	VVTLTELPSGNDTSG
36	TELPSGNDTSGLENK
37	NDTSGLENKTVVVTT
38	GLENKTVVVTTILES
39	KTVVVTTILESPYVM

GluA2 LBD

40	VTTILESPYVMMKKN
41	LESPYVMMKKNHEML
42	MMKKNHEMLEGNERY
43	NHEMLEGNERYEGYC
44	LEGNERYEGYCVDLA
45	ERYEGYCVDLAAEIA
46	GYCVDLAAEIAKHCG
47	DLAAEIAKHCGFKYK
48	EIAKHCGFKYKLTIV
49	CGFKYKLTIVGDGKY
50	YKLTIVGDGKYGARD
51	IVGDGKYGARDADTK
52	GKYGARDADTKIWNG
53	ARDADTKIWNGMVGE
54	DTKIWNGMVGELVYG
55	WNGMVGELVYGKADI
56	VGELVYGKADIAIAP
57	VYGKADIAIAPLTIT
58	ADIAIAPLTITLVRE
59	IAPLTITLVREEVID
60	TITLVREEVIDFSKP
61	REEVIDFSKPFMSLG
62	IDFSKPFMSLGISIM
63	KPFMSLGISIMIKKP
64	LGISIMIKKPQKSKP
65	GISIMIKKPQKSKPG
66	VERMVSPIESAEDLS
67	MVSPIESAEDLSKQT
68	IESAEDLSKQTEIAY
69	EDLSKQTEIAYGTLD
70	KQTEIAYGTLDSGST
71	YGTLDSGSTKEFFRR
72	SGSTKEFFRRSKIAV
73	KEFFRRSKIAVFDKM
74	RRSKIAVFDKMWTYM
75	IAVFDKMWTYMRSAE
76	DKMWTYMRSAEPSVF
77	YMRSAEPSVFVRTTA
78	AEPSVFVRTTAEGVA
79	VFVRTTAEGVARVRK
80	TTAEGVARVRKSKGK
81	GVARVRKSKGKYAYL
82	VRKSKGKYAYLLEST
83	KYAYLLESTMNEYIE
84	LLESTMNEYIEQRKP
85	NEYIEQRKPCDTMKV
86	EQRKPCDTMKVGGNL
87	CDTMKVGGNLDSKGY
88	KVGGNLDSKGYGIAT
89	NLDSKGYGIATPKGS
90	KGYGIATPKGSSLGN
91	IATPKGSSLGNAVNL
92	KGSSLGNAVNLAVLK
93	LGNAVNLAVLKLNEQ
94	VNLAVLKLNEQGLLD
95	VLKLNEQGLLDKLKN
96	NEQGLLDKLKNKWWY
97	LLDKLKNKWWYDKGE
98	LKNKWWYDKGECGSG
99	WYDKGECGSGGGDSK
100	GECGSGGGDSKEKTS
101	GGGDSKEKTSALSLS
102	GGDSKEKTSALSLSN

GluA3 Linker

103	MKVSGSRKAGYWNEY
104	GSRKAGYWNEYERFV
105	AGYWNEYERFVPFSD
106	NEYERFVPFSDQQIS
107	VPFSDQQISNDSSSS
108	DQQISNDSSSSENRT
109	NDSSSSENRTIVVTT
110	SSENRTIVVTTILES
111	RTIVVTTILESPYVM
112	VTTILESPYVMYKKN

GluA2 TMs

113	YEIWMCIVFAYIGVS
114	VFAYIGVSVVLFLVS
115	FGIFNSLWFSLGAFM
116	SLWFSLGAFMQQGCD
117	VWWFFTLIIISSYTA
118	IIISSYTANLAAFLT
119	VAGVFYILVGGLGLA
120	VGGLGLAMLVALIEF

Table S1. Sequences of peptides immobilized in the GluA2 array, related to Figure 4.

Peptides include residues 107-238, 348-510, 520-541, 568-587 and 601-811 of mature GluA2o. Peptide numbering and colour code match Figure 4 and S4. Array also includes residues 360-413 of the GluA3 NTD-LBD linker (shown in Figure S4A).

	γ-2		γ-8
	**Ex1**		**Ex1**
1	DYWLYSRGVCKTKSV	22 (1)	STDYWLYTRALICNT
2	YSRGVCKTKSVSENE	23 (2)	WLYTRALICNTTNLT
3	VCKTKSVSENETSKK	24 (3)	RALICNTTNLTAGDD
4	KSVSENETSKKNEEV	25 (4)	CNTTNLTAGDDGPPH
5	ENETSKKNEEVMTHS	26 (5)	NLTAGDDGPPHRGGS
6	SKKNEEVMTHSGLWR	27 (6)	GDDGPPHRGGSGSSE
7	EEVMTHSGLWRTCCL	28 (7)	PPHRGGSGSSEKKDP
8	THSGLWRTCCLEGNF	29 (8)	GGSGSSEKKDPGGLT
9	LWRTCCLEGNFKGLC	30 (9)	SSEKKDPGGLTHSGL
10	CCLEGNFKGLCKQID	31 (10)	KDPGGLTHSGLWRIC
11	GNFKGLCKQIDHFPE	32 (11)	GLTHSGLWRICCLEG
12	GLCKQIDHFPEDADY	33 (12)	SGLWRICCLEGLKRG
13	QIDHFPEDADYEADT	34 (13)	RICCLEGLKRGVCVK
14	FPEDADYEADTAEYF	35 (14)	LEGLKRGVCVKINHF
15	ADYEADTAEYFLRAV	36 (15)	KRGVCVKINHFPEDT
16	YEADTAEYFLRAVRA	37 (16)	CVKINHFPEDTDYDH
		38 (17)	NHFPEDTDYDHDSAE
		39 (18)	EDTDYDHDSAEYLLR
		40 (19)	YDHDSAEYLLRVVRA
		41 (20)	DHDSAEYLLRVVRAS
	**Ex2**		**Ex2**
17	VYISANAGDPSKSDS	42 (21)	VYISANAGEPGPKRD
18	ANAGDPSKSDSKKNS	43 (22)	ANAGEPGPKRDEEKK
19	DPSKSDSKKNSYSYG	44 (23)	EPGPKRDEEKKNHYS
20	SDSKKNSYSYGWSFY	45 (24)	KRDEEKKNHYSYGWS
21	DSKKNSYSYGWSFYF	46 (25)	DEEKKNHYSYGWSFY

Table S2. Sequences of peptides immobilized in the TARP arrays, related to Figure 5. Ex1 and Ex2 denote the two extracellular loops of TARPs. Peptide numbering matches Figure 5, which contains both TARPs in the same membrane. Numbers in parentheses correspond to peptides in 5C (central panel), which only contains γ-8. Colour code as in Figure 5.

Supplemental Experimental Procedures

Protein preparation

His-tagged γ-2 was produced in insect cells using a P1 baculovirus stock and a purification protocol provided by T. Nakagawa. The high-titer viral stocks were obtained following the Bac-N-Blue™ protocol from Invitrogen. Briefly, 1 x 10^6 SF9 cells were plated into a 25 cm^2 flask containing 5 ml of complete TNM-FH medium (Sigma Aldrich) and infected with 20 µl of the P1 viral stock. The flask was incubated at 27 °C until all the cells were lysed; 4 ml of this supernatant (the P2 viral stock) were used to infect 500 ml of SF9 cells at a density of 1 x 10^6 SF9 cells/ml. The culture was incubated for 1 week and the high-titer P3 viral stock was recovered by centrifugation and kept at 4 °C until infection. For protein production, 500-2000 ml of SF9 cells grown in SF900™ II serum-free medium (Gibco®) were infected with P3 high-titer viral stock and incubated for 48-72 hours. Cells were collected by centrifugation and re-suspended in 20 mM HEPES, pH 7.35, 320 mM sucrose, 5 mM EDTA, 5 mM EGTA, protease inhibitors (Roche) using a Dounce homogenizer. Cells were sonicated and membranes were washed in consecutive steps of re-suspension and ultracentrifugation using 1 M KI, 4 M urea and 20 mM imidazole in 20 mM HEPES, pH 7.35. Membrane protein solubilization was performed in 0.6 % decyl-maltoside (DM). After ultracentrifugation, the supernatant containing the His-tagged γ-2 was incubated with chelating sepharose beads (GE Healthcare) charged with Co^{2+} and the protein was eluted with 250 mM imidazole, 0.3 % DM, 20 mM HEPES, pH 7.35, 150 mM NaCl. Protein was concentrated, flash-frozen and conserved at -20 °C.

γ-2 concentration was estimated spectrophotometrically using the theoretical extinction coefficient in water, 39.2 mM^{-1} cm^{-1}.

The GluA2 NTD was produced and purified from stably transfected $GntI^{-}$ HEK293S cells as described previously (Rossmann et al., 2011). The purification consisted of cross-flow concentration and dialysis against 50 mM Tris pH 8, 150 mM NaCl; affinity purification with a HisTrap HP column (GE Healthcare) and gel filtration using a HiLoad™ Superdex™ 200 column (GE Healthcare). The pure glycosylated GluA2 NTD was concentrated in 20 mM HEPES, 150 mM NaCl, pH 7.4 and kept at 4 °C.

The GluA2i LBD (S1S2J R/flip construct) was sub-cloned with different tags in order to allow its detection in the peptide arrays. GluA2i LBD was first sub-cloned into the pGEX4T-2 vector (GE Healthcare), which contains an N-terminal GST-tag followed by a thrombin cleavage site. The plasmid was transformed into *Escherichia coli* Origami B (DE3) and grown at 37 °C to A_{600}=0.9-1. Cultures were cooled to 18 °C and expression was induced by the addition of 0.4 mM IPTG. Cultures were grown at 18 °C for 20 hours. Cells were sonicated and the lysate was incubated with Glutathione Sepharose 4b beads (GE Healthcare). The protein was eluted with 10 mM reduced-Glutathione and further purified using a Superdex 200 10/300 column (GE Healthcare). Protein was flash-frozen and kept at -20 °C until used.

A Flag-tag (DYKDDDDK) was also introduced by PCR at the C-terminus of GluA2i LBD and the gene was subcloned into a modified pET22b(+) plasmid containing an N-terminal His_8 affinity tag and a thrombin cleavage site. The plasmid was

transformed into *E. coli* Origami B (DE3) cells and cultures were grown at 37 °C until A_{600} reached 0.6–0.9. Cells were cooled to 18 °C and over-expression was induced with 0.4 mM IPTG for 20 hours. After cell lysis, protein was purified by affinity chromatography using a HisTrap HP column (GE Healthcare) followed by thrombin cleavage and gel filtration chromatography (HiLoad™ Superdex™ 200 column, GE Healthcare).

The same procedure was used to add a Flag-tag to the GluK2 LBD S1S2 construct (provided by Mark L. Mayer). The resulting protein includes a 19 peptide (MH_8 SSGLVPRGSAM) containing a thrombin cleavage site, the residues S398–K513 and P636–E775 of GluK2 connected by a GT linker and the Flag-tag at the C-terminus (DYKDDDDK). The protein production and purification protocol was the same as described for the GluA2i LBD Flag. Both Flag-tagged proteins were concentrated in 20 mM HEPES, 150 mM NaCl, pH 7.4, 1 mM L-glutamate and conserved at -20 °C.

Immunoprecipitations

HEK293 cells were transfected with vectors expression GluA2-IRES-EGFP and TARP γ-2 (IRES-RFP) at a ratio of 4:1, alternatively GluA2 was transfected into HEK cells stably expressing γ-2. After 2 days of expression, cells were washed with cold PBS, scraped into PBS containing protease inhibitors and centrifuged for 10 mins at 10K rpm for in an Eppendorf 5424R. Pellets were extracted in 1% CHAPS buffer containing in (mM): HEPES (20; pH 7.4), NaCl (150), EDTA (2), 1% CHAPS and protease inhibitors for 45 mins at 4°C. Extracts were centrifuged for 30 min at 4°C in an Eppendorf 5424R centrifuge at full speed. Supernatants were IPed with 5 µg of anti- γ-2 AB (Millipore) for 2 hrs at 4°C and precipitated with 10 µl of equilibrated

(in CHAPS buffer) protein A beads (Santa Cruz). Lysates were run on 4-12% Bolt gels (Invitrogen) and gels analyzed by Western blotting.

Peptide arrays

To analyze the interaction between AMPAR and TARPs we obtained peptide arrays synthesized by SPOT synthesis on Whatman 50 cellulose membranes (PepSpots™; JPT Peptide Technologies GmbH). AMPAR and TARP arrays contained 15-mer overlapping peptides shifted by 4 residues. The AMPAR array contained 128 peptides covering the lower lobe of GluA2 NTD, the NTD-LBD linkers with and without glycosylated residues (GluA2 and -A3 subunits), the GluA2 LBD and the four trans-membrane helices (**Figure 4B, Table S1**). The TARP array contained 46 peptides covering the two extracellular loops (Ex1 and Ex2) of γ-2 and γ-8 (**Figure 5, Table S2**).

Membranes were probed according to manufacturer instructions. As these membranes cannot be regenerated reliably, control experiments with the antibodies (ABs) were performed prior to incubation with the test proteins. Controls were essential and extensive to select the most adequate blocking agents, antibodies and protein tags, as some ABs showed false positives or high background in the cellulose membranes. Anti-His and anti-GST ABs showed signals in the control experiments whereas Flag M2 AB (Sigma), anti-AMPAR 2 (extracellular) AB (Alomone) or anti-Stargazin AB (Millipore) controls showed few false positives (**Figures 4C and 5B**).

For the protein binding assay, membranes were rinsed with methanol for 5 minutes, washed 3 times with TBS and incubated with blocking solution (5 % BSA in TBS)

for 2 hours at RT or overnight at 4 °C. After blocking, arrays were incubated with the test proteins overnight at 4 °C. The AMPAR array was incubated with ~2.8 μM γ-2 in blocking solution with 0.15 % DM. TARP arrays were incubated with ~1.6 μM GluA2 NTD, ~1.6 μM GluA2i LBD-Flag or ~1.6 μM GluK2 LBD-Flag in blocking solution. Membranes were washed three times in TBS and incubated for 1-2 hours at RT with the following ABs: anti-AMPAR 2 (extracellular) AB (Alomone) diluted 1:250 in blocking solution; anti-Stargazin AB (Millipore) diluted 1:1000 in blocking solution; and monoclonal anti-Flag M2 antibody (Sigma) diluted 1:1000 in blocking solution. After 3 washes with TBS membranes were incubated for 1 hour at RT with HRP-conjugated anti-rabbit AB (Pierce) (1:1500 in blocking buffer) or 2 hours at RT with HRP-conjugated anti-mouse AB (Pierce) (1:1000 in blocking buffer). Membranes were washed 3 times, developed using chemiluminescence (Amersham ECL solution) and images were captured electronically with a ChemiDoc™ MP Imaging System (Biorad) or on X-ray film. Films were scanned and edited with Photoshop CS4 using auto-tone editing followed by a change to gray-scale mode.

Heterologous expression

HEK293 or HEK293T cells (ATCC), cultured at 37°C and 5% CO_2 in DMEM (Gibco) supplemented with 10% fetal bovine serum and penicillin/streptomycin, were transiently transfected with plasmid DNA using Effectene (Qiagen) or Lipofectamine 2000 (Invitrogen). After 24-32 hours (Effectene) or 8-14 hours (Lipofectamine 2000), the cells were split onto poly-L-lysine-coated glass coverslips and electrophysiological recordings were performed 12-48 hours later. When co-transfected with TARPs, cells were grown in the presence of 30-50 μM 2,3-dioxo-6-

nitro-1,2,3,4-tetrahydrobenzo[f]quinoxaline-7-sulfonamide (NBQX; Tocris-ABCam) to avoid AMPAR-mediated toxicity.

Electrophysiology

Cells were visualized with an inverted microscope (Diaphot 200; Nikon) or fixed stage upright microscope (Axioskop FS1; Zeiss) and perfused with an 'external' solution containing (in mM): NaCl (145), KCl (3), $CaCl_2$ (2), $MgCl_2$ (1), glucose (10) and HEPES (10), adjusted to pH 7.4 with NaOH. Electrodes were fabricated from borosilicate glass (1.5mm o.d., 0.86mm i.d., Science Products GmbH or Harvard Apparatus) pulled with a PC-10 vertical puller (Narishige). When filled with an 'internal' solution, containing (mM): CsF (120), CsCl (10), EGTA (10), ATP-sodium salt (2), and HEPES (10), adjusted to pH 7.3 with CsOH, they had a final resistance of 2-5 MΩ. Macroscopic currents were recorded at room temperature (RT; 22-25 ˚C) from outside-out patches excised from GFP-positive cells and voltage-clamped at –60 mV. Currents were recorded with Axopatch 1D, 200A or 2D amplifiers, low-pass filtered at 10 kHz and digitized at 50 kHz using a Digidata 1440A interface with pClamp 9 or 10 software (Molecular Devices).

For the experiments described in **Figures 6 and S5** there were minor modifications of some experimental details; these reflected the established protocols of the two participating labs and, where compared directly, did not affect the outcome. For the experiments on TARP Ex1 mutants (**Figures 6 and S5**) the external solution contained (in mM) NaCl (145), KCl (2.5), $CaCl_2$ (1), $MgCl_2$ (1), glucose (10) and HEPES (10), adjusted to pH 7.3 with NaOH. The internal solution contained (in mM) CsCl (145), EGTA (1), ATP-magnesium salt (4), NaCl (2.5) and HEPES (10), adjusted to pH 7.3 with CsOH. Electrodes were coated with Sylgard (Dow Corning

184) and had a final resistance of 6-14 MΩ. Aside from experiments in which KA/Glu ratios were measured, spermine tetrahydrochloride (100 μM; Tocris Bioscience or Sigma Aldrich) was added to the internal solution.

Agonist application to excised patches

Rapid agonist application was achieved by switching between a continuously flowing control solution and an agonist-containing solution, applied via a theta-barrel application tool made from borosilicate glass (2mm o.d.; Hilgenberg GmbH) pulled to a tip diameter of ~200 μm and mounted on a piezoelectric translator (Burleigh PZS 200 or LSS-3000/PZ-150M; EXFO Life Sciences & Industrial Division). At the end of each experiment, the adequacy of the solution exchange was assessed by destroying the patch and measuring liquid-junction current at the open pipette (typical 10% - 90% solution exchange time of ~200 μs).

Kinetics of AMPAR-mediated responses

Desensitization of AMPARs was examined in response to 100 ms applications of 10 mM L-glutamate. The averaged currents were fitted using a double-exponential function to calculate the weighted time constant of desensitization ($\tau_{w,des}$) according to:

$$\tau_{w,des} = \tau_f \left(\frac{A_f}{A_f + A_s}\right) + \tau_s \left(\frac{A_s}{A_f + A_s}\right)$$

where A_f and τ_f are the amplitude and time constant of the fast component of desensitization and A_s and τ_s are the amplitude and time constant of the slow

component of desensitization. The weighted time constant of deactivation ($\tau_{w,deact}$) was determined in a similar manner, by fitting the current decay following 1 ms applications of 10 mM L-glutamate. In some cases the desensitization or deactivation time course was best described by a single exponential. The steady state-to-peak ratio (SS/peak) was determined as the current at the end of the 100 ms pulse divided by the peak current.

Recovery from desensitization was assessed by a paired-pulse protocol where a 100 ms desensitizing pulse was followed by a 10 ms pulse in increasing intervals. The relative response to the second pulse (usually the average of three consecutive runs) was then plotted against time elapsed from the first pulse and the time course fitted with a single-exponential function to obtain the time constant of recovery (τ_{rec}).

Relative kainate efficacy

The effects of TARPs on the efficacy of the partial agonist kainate (KA) were determined by measuring changes in KA/Glu ratios. Each patch was exposed to L-glutamate (500 μM; 15 applications of 100 ms duration, –60 mV), then KA (500 μM), and then L-glutamate again. Both control and agonist solutions contained 100 μM cyclothiazide to block AMPAR desensitization. The amplitude of the steady state current response to KA was compared to average amplitude of the steady state current responses to L-glutamate obtained before and after KA application.

Non-stationary fluctuation analysis (NSFA)

To deduce channel properties from macroscopic responses, L-glutamate (10 mM) was applied to outside-out patches (100 ms duration, 1 Hz, V_{hold} –60mV) and the ensemble

variance of all successive pairs of current responses were calculated (Conti et al., 1980). The single channel current (*i*) and the total number of channels in the patch (*N*) were determined by plotting this ensemble variance against mean current (I) and fitting with a parabolic function (Sigworth, 1980):

$$\sigma^2 = \sigma_B^2 + \left(i\bar{I} - \left(\frac{\bar{I}^2}{N}\right)\right)$$

where σ^2_B is the background variance. The weighted-mean single-channel conductance was determined from the single-channel current and the holding potential. No correction for liquid-junction potential was used.

Current-voltage (*I-V*) plots and the quantification of rectification

I-V plots were generated from the peak current response to 1 ms applications of 10 mM glutamate. The voltage was stepped from –100 mV to +60 mV in 10 mV increments. Mean current amplitudes at each voltage were normalized to the peak current at –100 mV and plotted against membrane potential. The relationships were fitted with 8th or 9th order polynomials. The rectification index (RI) was determined from the *I-V* relationship of each patch as the ratio of slope conductance at positive (+40 mV to +60 mV) and negative voltages (–40 mV to –60 mV). Thus, for a completely linear *I-V* relationship RI would equal 1.

Analysis and statistics

Recordings were analyzed using IGOR Pro (Wavemetrics Inc.) with NeuroMatic (J. Rothman, UCL; http://www.neuromatic.thinkrandom.com). Summary data are presented in the text as the mean ± SEM from *n* patches and in the figures as bar plots of the group mean, with error bars denoting SEM. Comparisons involving two data

sets only were performed using a two-sided Welch two-sample *t* test. All analyses involving data from three or more groups were performed using one- or two-way analysis of variance (Welch heteroscedastic *F* test) followed by pairwise comparisons using two-sided Welch two-sample *t* tests (with Holm's sequential Bonferroni correction for multiple comparisons). Differences were considered significant at $P < 0.05$. Statistical tests were performed using Prism 4.0/6.0 (GraphPad Software Inc.) or R (version 3.0.2, The R Foundation for Statistical Computing, http://www.r-project.org/) and RStudio (version 0.98.313, RStudio, Inc.).

Supplemental References

Conti, F., Neumcke, B., Nonner, W., and Stampfli, R. (1980). Conductance fluctuations from the inactivation process of sodium channels in myelinated nerve fibres. J Physiol *308*, 217-239.

Sigworth, F.J. (1980). The variance of sodium current fluctuations at the node of Ranvier. J Physiol *307*, 97-129.
