# Supplementary material for: Mapping the Interaction Sites between AMPA Receptors and TARPs Reveals a Role for the Receptor N-Terminal Domain in Channel Gating
Source: Cell Rep. 2014 Oct 16;9(2):728–40. doi: 10.1016/j.celrep.2014.09.029 (PMC4405707; doi:10.1016/j.celrep.2014.09.029)
Supplement: Document S2. Article plus Supplemental Information [file mmc2.pdf]

Cell Reports

Article

Mapping the Interaction Sites between AMPA Receptors and TARPs Reveals a Role for the Receptor N-Terminal Domain in Channel Gating

Graphical Abstract

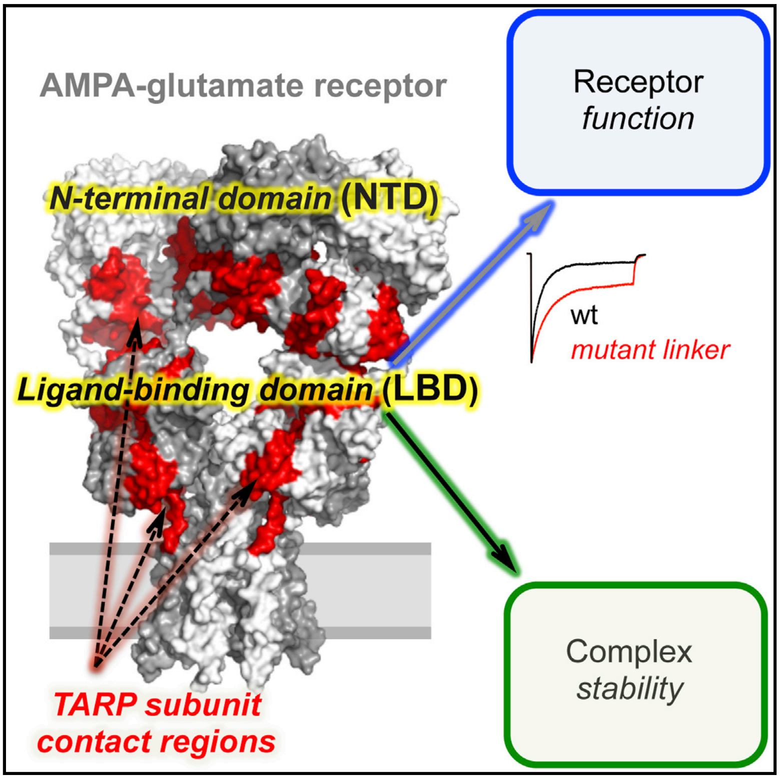

Highlights

The NTD linker has a TARP-dependent and TARP-specific impact on AMPAR gating

Peptide arrays reveal binding of TARPs to both extracellular domains of AMPARs

A structural reorganization of AMPARs is triggered by TARP binding

Authors

Ondrej Cais, Beatriz Herguedas, ..., Mark Farrant, Ingo H. Greger

Correspondence

ig@mrc-lmb.cam.ac.uk

In Brief

Gating properties of synaptic AMPA-type glutamate receptors (AMPARs) are modulated by the transmembrane AMPAR regulatory proteins (TARPs), yet knowledge about their binding on a molecular level is limited. Here, Cais et al. map this interaction on both partner molecules and reveal a functional role for the receptor N-terminal domain.

Cais et al., 2014, Cell Reports 9, 728–740
October 23, 2014 ©2014 The Authors
http://dx.doi.org/10.1016/j.celrep.2014.09.029

OPEN ACCESS
CellPress

Cell Reports
Article

Mapping the Interaction Sites between AMPA Receptors and TARPs Reveals a Role for the Receptor N-Terminal Domain in Channel Gating

Ondrej Cais,[1] Beatriz Herguedas,[1] Karolina Krol,[2] Stuart G. Cull-Candy,[2] Mark Farrant,[2] and Ingo H. Greger[1,*]
[1]Neurobiology Division, MRC Laboratory of Molecular Biology, Cambridge CB2 0QH, UK
[2]Department of Neuroscience, Physiology and Pharmacology, University College London, London WC1E 6BT, UK
*Correspondence: ig@mrc-lmb.cam.ac.uk
http://dx.doi.org/10.1016/j.celrep.2014.09.029
This is an open access article under the CC BY license (http://creativecommons.org/licenses/by/3.0/).

SUMMARY

AMPA-type glutamate receptors (AMPARs) mediate fast neurotransmission at excitatory synapses. The extent and fidelity of postsynaptic depolarization triggered by AMPAR activation are shaped by AMPAR auxiliary subunits, including the transmembrane AMPAR regulatory proteins (TARPs). TARPs profoundly influence gating, an effect thought to be mediated by an interaction with the AMPAR ion channel and ligand binding domain (LBD). Here, we show that the distal N-terminal domain (NTD) contributes to TARP modulation. Alterations in the NTD-LBD linker result in TARP-dependent and TARP-selective changes in AMPAR gating. Using peptide arrays, we identify a TARP interaction region on the NTD and define the path of TARP contacts along the LBD surface. Moreover, we map key binding sites on the TARP itself and show that mutation of these residues mediates gating modulation. Our data reveal a TARP-dependent allosteric role for the AMPAR NTD and suggest that TARP binding triggers a drastic reorganization of the AMPAR complex.

INTRODUCTION

AMPA-type glutamate receptors (AMPARs) mediate fast excitatory transmission and are crucial for various forms of synaptic plasticity (Bredt and Nicoll, 2003; Cull-Candy et al., 2006). Their varied kinetic behavior (Mosbacher et al., 1994), as well as their calcium permeability and voltage-dependent block by polyamines (Cull-Candy et al., 2006; Geiger et al., 1995), varies between brain regions and appear to be adapted to the specific function of a given circuit (Jonas, 2000; Trussell, 1998). These properties depend on the nature and mRNA processing status of the four pore-forming subunits (GluA1–GluA4) (Traynelis et al., 2010; Jonas, 2000) and on the type and stoichiometry of AMPAR auxiliary subunits (Jackson and Nicoll, 2011).

Four families of auxiliary subunits have been identified: transmembrane AMPAR regulatory proteins (TARPs) (Tomita et al., 2005; Turetsky et al., 2005), cornichons (Schwenk et al., 2009), CKAMP44 (von Engelhardt et al., 2010), and GSG1L (Schwenk et al., 2012; Shanks et al., 2012). Most of these alter AMPAR gating and confer effects that can be specific for a given synapse or cell. TARPs were the first identified bona fide AMPAR auxiliary proteins, modifying both AMPAR function and trafficking. Based on their modulatory actions, TARPs have been classified as type 1a (γ-2 and γ-3), type 1b (γ-4 and γ-8), and type 2 (γ-5 and γ-7) (Kato et al., 2010). TARP-like modulation of AMPARs has also been seen in invertebrates (Walker et al., 2006; Wang et al., 2008) and thus appears highly conserved.

The precise nature of the AMPAR/TARP interaction and thus the mechanism underlying gating modulation are poorly understood. Both the AMPAR transmembrane region and the ligand binding domain (LBD) have been implicated in TARP interactions responsible for the modulation of ligand efficacy, pharmacology, gating, and pore properties (Jackson and Nicoll, 2011). Experiments using domain swapping between subtypes have identified TARP regions that are involved in regulating AMPARs. These include the extracellular loop (Ex1), the transmembrane sector, and the C terminus. Specifically, the TARP C tail appears critical for receptor trafficking and mediation of kinetic effects, while Ex1 influences both the efficacy of the partial agonist kainate and AMPAR kinetics (Tomita et al., 2005; Turetsky et al., 2005).

The most distal AMPAR domain, the N-terminal domain (NTD), is expected to be beyond the "reach" of the associated TARP. Apart from a role in subunit assembly, no clear function has been ascribed to this large and most sequence-diverse domain (Hansen et al., 2010; Kumar and Mayer, 2013), although deletion of the NTD slows desensitization kinetics (Bedoukian et al., 2006; Möykkynen et al., 2014; Pasternack et al., 2002). In stark contrast, the NTD of the N-methyl-D-aspartate (NMDA)-type glutamate receptor (NMDAR) mediates allosteric regulation of channel open probability (Paoletti, 2011) in a subunit-specific manner, rendering the NTD an important target for selective NMDAR drugs (Mony et al., 2009). NTD-mediated allostery in NMDARs has been shown to involve the ~16-residue peptide linkers that connect the NTD to the LBD (Gielen et al., 2009; Mony et al., 2011; Yuan et al., 2009).

Here we show that the AMPAR NTD plays a previously unrecognized role in signaling. Shortening of the NTD-LBD linkers altered desensitization rates and recovery from the desensitized state and increased the steady-state response. These gating effects were TARP dependent and TARP specific. Using peptide

OPEN ACCESS
CellPress

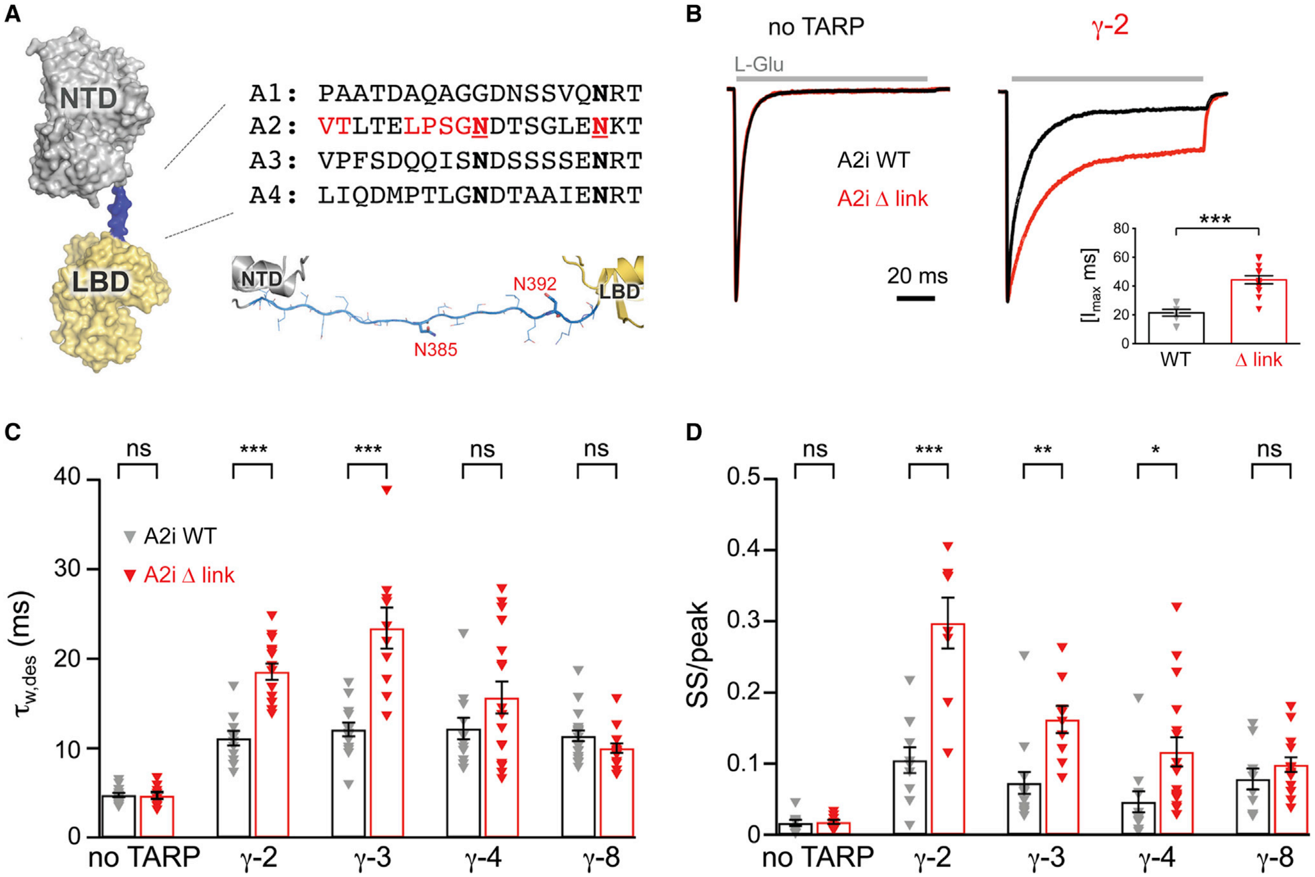

Figure 1. The NTD-LBD Linker Influences TARP-Dependent Changes in AMPAR Gating

(A) Structure of the extracellular region of a GluA2 subunit, showing the NTD (gray), linker (blue), and LBD (yellow) (adapted from PDB: 3KG2). Sequence alignment of the rat GluA1-4 NTD linkers, red residues have been mutated in Δ link with VTxxxLPSG deleted and the two asparagines (N385 and N392 bold, underlined) mutated to Asp and Gln, respectively (analogous to PDB 3KG2). Model of a complete, stretched NTD linker is shown beneath the alignment.

(B) Representative normalized current responses evoked by 100 ms glutamate application (gray bars). Outside-out patches were pulled from cells transfected with GluA2iQ WT or Δ link in the absence or presence of γ-2 and the decay of the current (−60 mV) analyzed to determine the time constant of desensitization and the magnitude of the steady state component. (Inset) Pooled data (mean ± SEM) showing the difference in charge transfer (normalized to the peak) during the 100 ms glutamate application (***$p < 0.001$, Welch t test).

(C) Pooled data showing the desensitization time constants for GluA2iQ WT or Δ link expressed alone (n = 14 and 10), or with TARPs γ-2 (n = 11 and 15), γ-3 (n = 14 and 10), γ-4 (n = 12 and 17), or γ-8 (n = 20 and 15). Currents were fitted with a two-exponential function. The weighted time constant ($\tau_{w,des}$) is shown ± SEM. Two-way ANOVA indicated a significant main effect of TARP ($F_{4,111} = 82.28$, $p = 2.66 \times 10^{-32}$), no significant main effect of linker mutation ($F_{1,111} = 1.73$, $p = 0.19$) and a significant interaction between linker and TARP effects ($F_{4,111} = 10.05$, $p = 5.61 \times 10^{-7}$). Asterisks denote significance of difference between WT and Δ link for each TARP condition (*$p < 0.05$, **$p < 0.01$, ***$p < 0.001$; Welch t test).

(D) Pooled data showing the ratio of current at the end of the 100 ms glutamate application (steady-state [SS]) to the peak response. The data are plotted and analyzed as in (C), for GluA2iQ WT or Δ link expressed alone (n = 10 each), or with γ-2 (n = 11 and 8), γ-3 (n = 14 and 9), γ-4 (n = 12 and 17), or γ-8 (n = 10 and 15). There were significant main effects of TARP subtype ($F_{4,106} = 42.74$, $p = 2.64 \times 10^{-21}$) and linker mutation ($F_{1,106} = 5.04$, $p = 0.027$) and a significant interaction between linker and TARP effects ($F_{4,106} = 8.89$, $p = 3.16 \times 10^{-6}$). Asterisks are as in (C).

arrays, we mapped the GluA2/TARP contact region and identified TARP binding sites on the NTD. On the LBD, TARP contact points mapped to functionally critical sites, including the ligand binding cleft, the flip/flop region, and the linkers that connect the LBD to the ion channel. We also determined the sites on the TARP that are contacted by the AMPAR and assessed their functional role using corresponding TARP mutants. Our results provide detailed insights into the molecular interactions of TARPs with AMPARs and show that these include the distal NTD. This subunit-specific TARP regulatory site may permit fine tuning of AMPAR signaling and provide a target for subunit-selective drugs.

RESULTS

The NTD-LBD Linker Mediates TARP-Dependent Changes of AMPAR Gating

The iGluR extracellular region comprises two layers (Sobolevsky et al., 2009; Suzuki et al., 2014), a unique architecture not observed in other ligand-gated channels. In AMPARs, the function of the distal NTD layer is unknown. This layer is loosely connected to the LBD via ~17 residue N-glycosylated linkers (Figure 1A). As these linkers may function as potential "output" regions for NTD-mediated allostery, we created linker mutations

OPEN ACCESS
CellPress

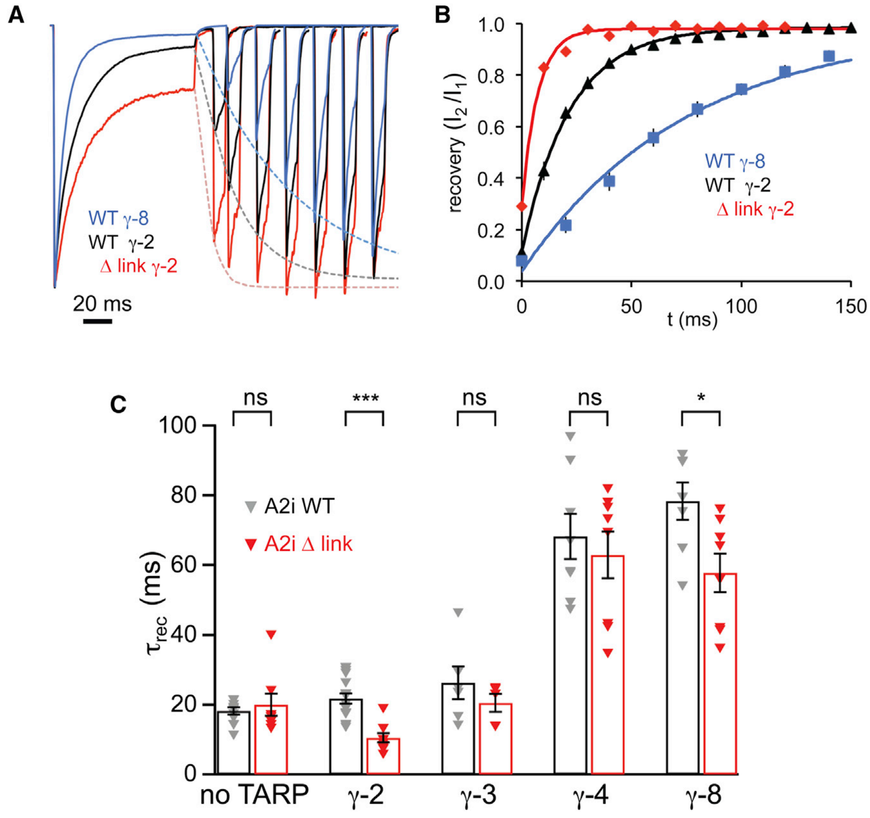

Figure 2. The Δ Link Mutation Accelerates Recovery from Desensitization in the Presence of TARPs

(A) Representative traces illustrating recovery from desensitization (averages of three trials in each case). A 100 ms pulse of 10 mM L-glutamate was followed, at increasing intervals, by a 10 ms test pulse, and the recovery in the amplitude of the test response was fitted by a monoexponential function (dashed lines). Currents were normalized to the first peak, and for clarity, only selected traces are shown.

(B) Summary of the data presented in (A). Relative currents at individual time points are shown ± SEM (error bars masked by the symbols). The solid lines are monoexponential fits of the average values (giving time constants of 7.3, 21.8, and 75.6 ms for GluA2 Δ link + γ-2, GluA2 WT + γ-2, and GluA2 WT + γ-8, respectively).

(C) Pooled data for the time constant of recovery from desensitization (τ_{rec}) for GluA2iQ WT and Δ link expressed alone (n = 11 and 8, respectively) or with TARPs γ-2 (n = 17 and 9), γ-3 (n = 6 and 5), γ-4 (n = 8 each), or γ-8 (n = 7 and 8) (shown ± SEM). Two-way ANOVA showed significant main effects of TARP subtype ($F_{4,\ 77}$ = 62.61, p = 1.91 × 10^{-23}) and linker mutation ($F_{1,\ 77}$ = 25.58, p = 4.14 × 10^{-6}) and a significant interaction between linker and TARP effects ($F_{4,\ 77}$ = 3.14, p = 0.019). Asterisks denote significance of difference between WT and Δ link for each TARP condition (*p < 0.05, ***p < 0.001; Welch t test).

See also Figure S1.

and assayed their effect on AMPAR function. Initially, we recreated the modifications that had been used in the GluA2 crystal structure, $GluA2_{cryst}$ (Sobolevsky et al., 2009), as this provided direct structural information on the packing between NTD and LBD. Thus, we deleted six residues plus two N-glycosylation sites in GluA2(Q_{607}) flip, resulting in the GluA2i linker mutation, Δ link (sequence in red; Figure 1A).

While the linker mutation produced no change in channel kinetics in the absence of TARP γ-2 (Figure 1B, left), in the presence of γ-2, the mutant exhibited a pronounced slowing of entry into the desensitized state ($\tau_{w\ des}$ 11.10 ± 0.82 ms for GluA2i wild-type [WT] versus 18.56 ± 0.92 ms for Δ link; n = 11 and 15, respectively) and an ~3-fold increase in the steady-state current (Figures 1B–1D). These changes resulted in a more than 2-fold increase in normalized charge transfer of TARPed Δ link (inset in Figure 1B). Deactivation of the TARPed receptor was unaltered by the linker mutation ($\tau_{w,\ deac}$ 1.07 ± 0.10 ms for GluA2i WT versus 1.09 ± 0.16 ms for Δ link; n = 15 and 10, respectively).

To determine if this behavior was specific to γ-2, we measured desensitization kinetics of Δ link when associated with other TARPs. With γ-3, the effects of the linker mutation were similar to those seen with γ-2. However, in the presence of γ-8, the linker mutation neither slowed desensitization nor increased the steady-state component (Figures 1C and 1D). These data reveal that an alteration in the AMPAR NTD-LBD linker affects channel gating in a TARP-dependent and TARP-selective manner.

The NTD-LBD Linker Modulates Recovery from Desensitization

We next investigated whether the NTD linker has a wider role in AMPAR function and could affect other aspects of gating that are regulated by TARPs. TARPs are also known to accelerate recovery from desensitization for GluA1 receptors (Gill et al., 2012; Priel et al., 2005); our experiments showed that the influence of TARPs on recovery from desensitization depended both on the AMPAR subtype and the TARP isoform. Thus, in contrast with GluA1, recovery from desensitization of GluA2i was unaffected by the presence of γ-2 or γ-3 (type 1a TARPs) and was in fact markedly *slowed* by γ-4 and γ-8 (type 1b) (Figure 2 and Figure S1A available online). A similar pattern was also observed with GluA3i (Figure S1B), suggesting that accelerated recovery is specific to GluA1.

The Δ link mutation resulted in an acceleration of GluA2i recovery from desensitization. This effect was again TARP subtype specific and was observed with γ-2 and γ-8, but not with γ-3 or γ-4 (Figure 2). Accelerated recovery together with reduced desensitization (Figures 1B and 1C) is expected to boost charge transfer through TARPed Δ link. Thus, changes in the NTD-LBD linker have a wider role in AMPAR gating that is TARP dependent.

Specific Features of the NTD-LBD Linker Mediate Gating Effects

We next pinpointed the minimal regions of the linker able to mediate gating effects. We focused on the core deletion, LPSG,

OPEN ACCESS
CellPress

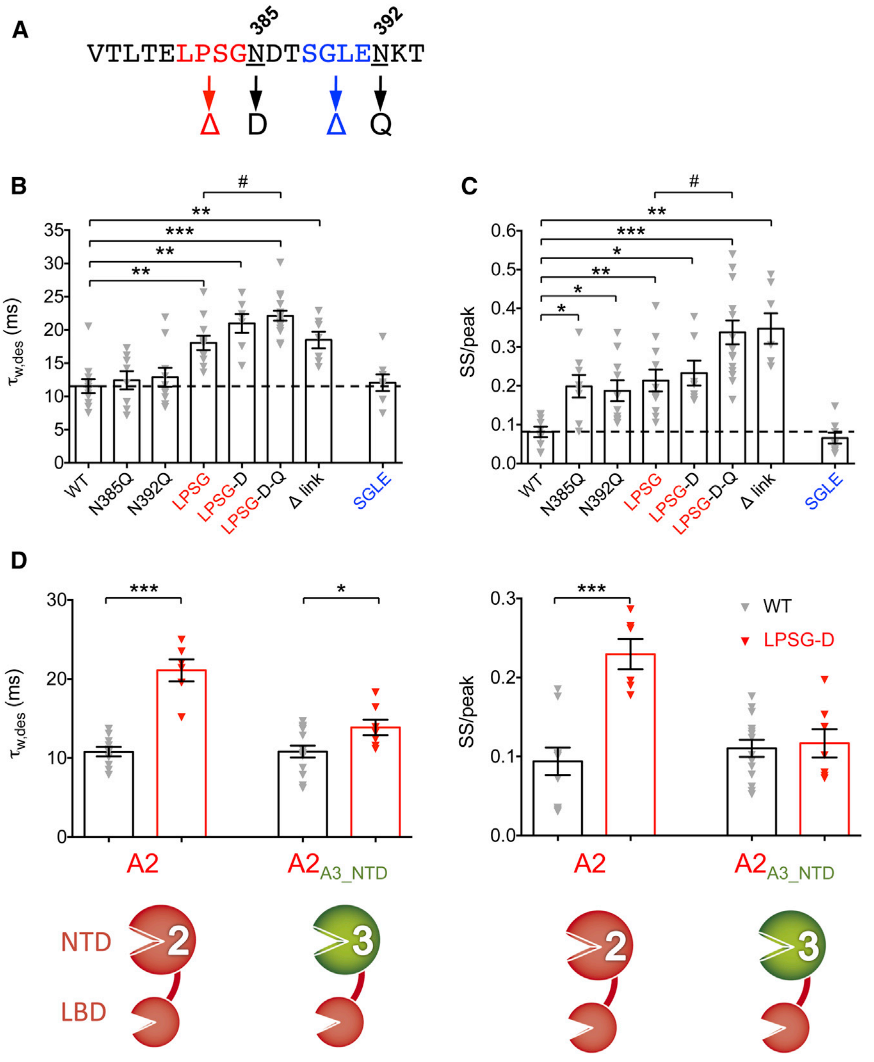

Figure 3. Gating Changes Are Mediated by Specific Structural Features of the NTD Linker

(A) Sequence of the GluA2 NTD-LBD linker with the glycosylation sites and the amino acid quadruplets deleted in these experiments highlighted in red and blue.

(B) Pooled data (mean ± SEM) showing the effects on desensitization ($\tau_{w,des}$) of NTD-LBD linker mutations in GluA2i in presence of γ-2. LPSG denotes a construct with these four amino acids deleted; LPSG-D combines this with the N385D mutation, and LPSG-D-Q additionally includes N392Q. Following one-way ANOVA ($F_{7,\ 25.7} = 14.69$, $p = 1.52 \times 10^{-7}$), pairwise comparisons showed that $\tau_{w,des}$ was slower for LPSG (n = 11), LPSG-D (n = 6), LPSG-D-Q (n = 16), and Δ link (n = 7) compared with WT (n = 11) (**$p < 0.01$, ***$p < 0.001$) and was significantly slower for LPSG-D-Q compared with LPSG (#$p < 0.05$) (Welch t tests). There was no significant effect of the glycosylation mutations N385D (n = 8) or N392Q (n = 10) and no effect of the alternative deletion mutant, SGLE (n = 8).

(C) Pooled data for the steady state-to-peak ratio (SS/peak; presented and analyzed as in B). Following one-way ANOVA ($F_{7,\ 26.5} = 17.18$, $p = 2.34 \times 10^{-8}$), pairwise comparisons showed that the SS/peak ratio was greater for N385D (n = 8), N392Q (n = 10), LPSG (n = 11), LPSG-D (n = 7), LPSG-D-Q (n = 16), and Δ link (n = 7) compared with WT (n = 9) (**$p < 0.01$, ***$p < 0.001$) and significantly greater for LPSG-D-Q compared to LPSG (#$p < 0.05$) (Welch t tests). Again, no effect of the alternative deletion mutant, SGLE (n = 8).

(D) Pooled data (± SEM) comparing the effects of the LPSG-D mutation in GluA2 and in a chimeric construct where the NTD of GluA2 was replaced by that of GluA3. (Left) $\tau_{w,des}$ was increased by LPSG-D in both GluA2 (n = 10 and 6) and $GluA2_{A3\text{-}NTD}$ (n = 14 and 7) (***$p < 0.001$ and *$p < 0.05$; Welch t tests). Two-way ANOVA showed a significant interaction between NTD and linker ($F_{1,\ 33} = 11.62$, $p = 0.0017$). Similar results were seen for the SS/peak ratio (right); in this case, the ratio was increased by LPSG-D in GluA2 (***$p < 0.001$) but not in $GluA2_{A3\text{-}NTD}$. Again, two-way ANOVA showed a significant interaction between NTD and linker ($F_{1,\ 33} = 13.10$, $p = 9.75 \times 10^{-4}$), confirming that the effect of the LPSG-D linker mutation was NTD-type specific.

See also Figure S2.

and on two mutations (N385D and N392Q) that abolish N-glycosylation (Figure 3A) and thus may alter linker flexibility. Deletion of LPSG slowed GluA2i/γ-2 desensitization to a similar extent to that seen with the complete modification (Δ link) (Figure 3B). This phenotype was enhanced when combined with the glyco double null mutant (LPSG-N385D/N392Q), whereas mutation of the two glycosylation sites alone had no significant impact (Figure 3B). A similar trend was observed for the steady-state response except that N385D and N392Q alone also produced significant effects (Figure 3C). Hence, the four-residue linker deletion "LPSG" is necessary and sufficient to confer the alterations in gating seen with the Δ link mutant. As linkers have been suggested to encode structural states (Ma et al., 2011), our observation raised the question of whether these effects on gating were merely due to linker shortening or if they resulted from specific structural effects. To address this, we introduced an alternative four-residue deletion SGLE (S_{388}-E_{391}) further downstream (Figure 3A). Unlike ΔLPSG, the SGLE deletion did *not* slow GluA2i/γ-2 desensitization and did not increase the steady state current (Figures 3B and 3C), indicating that structural changes, rather than linker shortening alone, are important in mediating TARP-dependent alterations in GluA2i gating.

To extend this finding, we introduced a deletion into the linker of GluA3i, at a position analogous to ΔLPSG in GluA2i (Figure S2A). This deletion (ΔQISS) also slowed desensitization in the presence of γ-2, and its effect was magnified when combined with the glyco null mutation N387D (QISS-N387D; Figure S2B). However, as observed with GluA2i, a four-residue deletion introduced further downstream, ΔSSSE (analogous to GluA2i ΔSGLE), had no significant effect (Figure S2B). Hence, NTD-LBD linkers have a general role in the control of AMPAR gating.

TARP-Dependent Reorientation of the NTD via the Linkers

How do NTD linker deletions affect gating of the AMPAR-TARP complex? While deletion of the NTD is known to alter desensitization kinetics (Bedoukian et al., 2006; Möykkynen et al., 2014; Pasternack et al., 2002), this domain has not been implicated in TARP modulation to date. In fact, previous work has questioned a role for the NTD in TARP function (Bedoukian et al., 2006; Morimoto-Tomita et al., 2009; Tomita et al., 2007). Similarly, in our hands, the gating properties of AMPARs lacking the NTD (GluA2i-ΔNTD), including desensitization kinetics and kainate efficacy, retained modulation by γ-2 (Figures S2C–S2E). However, these observations do not rule out a functional role for the NTD, which may trigger a TARP-dependent reorganization of the receptor (Figure S6B).

The position dependence of the deletions described in Figures 3A–3C suggests that the linker might facilitate a "preferred" orientation of the NTD relative to the LBD, perhaps to optimize TARP binding and thereby enable the TARP-dependent slowing of desensitization (Figure 1). As the NTD is highly sequence diverse, with a sequence identity of ~55% between AMPAR subunits, we replaced the NTD core (lacking the linker) from GluA2i WT and GluA2i ΔLPSG-D with that of GluA3 (Figure 3D, bottom). We reasoned that if a selective positioning of NTD to LBD created an optimal TARP binding site, then this replacement would markedly alter this interaction surface. When GluA2i receptors contained the GluA3 NTD, the effects of the ΔLPSG-D mutation on desensitization and steady-state response were attenuated drastically (Figure 3D). This is consistent with the view that a specific orientation of the NTD-LBD may allow an optimal TARP interaction site and hence greater TARP efficacy.

The NTD Stabilizes the AMPAR-TARP γ-2 Complex

To establish whether the NTD directly mediates interaction with TARPs, we first used immunoprecipitation (IP) to test if the NTD contributes to stabilizing the AMPAR-TARP complex. We transfected either GluA2i WT or GluA2i-ΔNTD into HEK293T cells stably expressing TARP γ-2, extracted proteins under mild detergent conditions (Nakagawa et al., 2006) and IPed GluA2i/γ-2 complexes with an anti-γ-2 antibody. As shown in Figure 4A, the fraction of GluA2i-ΔNTD associating with γ-2 was markedly reduced (lanes 3 + 4) when compared with GluA2i WT (lanes 1 + 2). The ratio of IPed GluA2i WT to GluA2i-ΔNTD was ~3-fold (2.9 ± 0.5; n = 5), when normalized to the input. TARP expression between conditions was comparable (Figure 4A, lower panel), and a similar association pattern was evident in the reverse experiment, where γ-2 was IPed with GluA2 (Figure S3A). Conversely, IP of GluA2i Δ link with γ-2 was similar to GluA2i WT (Figure S3B) and was not enhanced as one may have expected from the functional data (Figure 1).

Reduced association with γ-2 in absence of the AMPAR NTD was also observed for GluA1 (data not shown) and for other GluA2 isoforms, namely unedited GluA2i-Q_{607} and for the alternatively spliced GluA2-flop variety (GluA2o-R_{607}). While there was no obvious difference in TARP association between the WT isoforms, we noted an isoform-specific difference between the ΔNTD mutants (Figure 4A, lanes 3–8). Specifically, the Q to R switch at the channel pore reduced coIP by ~2-fold (2.2 ± 0.6, n = 5; lanes 4 versus 6) and the flop Δ-NTD mutant precipitated ~3-fold (2.9 ± 0.3, n = 5) less efficiently than its flip counterpart (lanes 4 versus 8). These results imply that the NTD contributes to complex stability and that there are multiple regions on the AMPAR that mediate association with TARP auxiliary subunits.

Delineating the TARP γ-2 Interaction Regions on GluA2

Thus far, our data suggested a reorganization of AMPARs when associating with TARPs. This prompted us to identify TARP binding sites on the receptor, which are currently unknown. We utilized peptide arrays, which provide semiquantitative maps of protein interaction regions (Katz et al., 2011; Shanks et al., 2014). We first probed an array of overlapping 15 amino acid peptides representing GluA2 with TARP γ-2 (Table S1). The array covered the NTD lower lobe, the NTD-LBD linker, the LBD and the transmembrane sector (schematic in Figure 4B). TARP γ-2 binding was revealed with an anti-γ-2 antibody (Supplemental Experimental Procedures).

As shown in Figures 4C, 4D, and S4, TARP interaction sites mapped to the LBD, the transmembrane region and indeed included the NTD. Interestingly, the NTD linker region was devoid of γ-2 binding. Even GluA2 peptides mimicking glycosylation (with GlcNAc-β[1-4]-GlcNAc) at the two N-glycosylation sites, N385 and N392, were negative (data not shown), suggesting that the linker is not directly contacted by the TARP but facilitates a specific orientation of the NTD (which is altered in Δ link). A similar pattern was observed with GluA3 where the NTD core that precedes the linker interacted with γ-2, whereas the linker itself was mostly devoid of signal (Figure S4A). Below we give a more detailed description of the γ-2 contact points on GluA2.

Regions of the NTD that Interact with γ-2

TARP contact regions mapped to various points on the NTD (Figures 4C, 4D, and S4). These included the front helices F and H, which have previously been implicated in NTD dynamics; they exhibit structural heterogeneity (Sukumaran et al., 2011) and undergo fluctuations when measured at a single-molecule level (Jensen et al., 2011) and in molecular dynamics simulations (Dutta et al., 2012). Of note, these helices also form an interface between NTD dimers (Jin et al., 2009; Sobolevsky et al., 2009), which may be disrupted by TARP association in the AMPAR tetramer (orange region in Figure S4B). Interaction sites also mapped to the side (close to helix H), the back of the NTD (along helix D), and across the NTD "floor" (Figures 4D, S4B, and S4C).

Regions of Interaction on the GluA2 LBD and the Transmembrane Sector

The LBD has been suggested as a key TARP modulatory target (Kato et al., 2010; Tomita et al., 2006, 2007). Our identification of TARP contact points on strategic regulatory sites on the LBD offers an explanation for these observations. These included the upper and lower "lip" of the LBD clamshell (regions A_1, A_2 in Figures 4C and 4D), the LBD-TMD linker region (region B), and the alternatively spliced flip/flop cassette (region C; Figures 4D and S4D) (Sommer et al., 1990).

TARP interaction with regions A and B suggests how TARP binding could modulate AMPAR gating kinetics and agonist efficacy. Region A stretches across both lobes of the LBD clamshell, extending from beta strand 2 in the upper lobe down to

OPEN ACCESS
CellPress

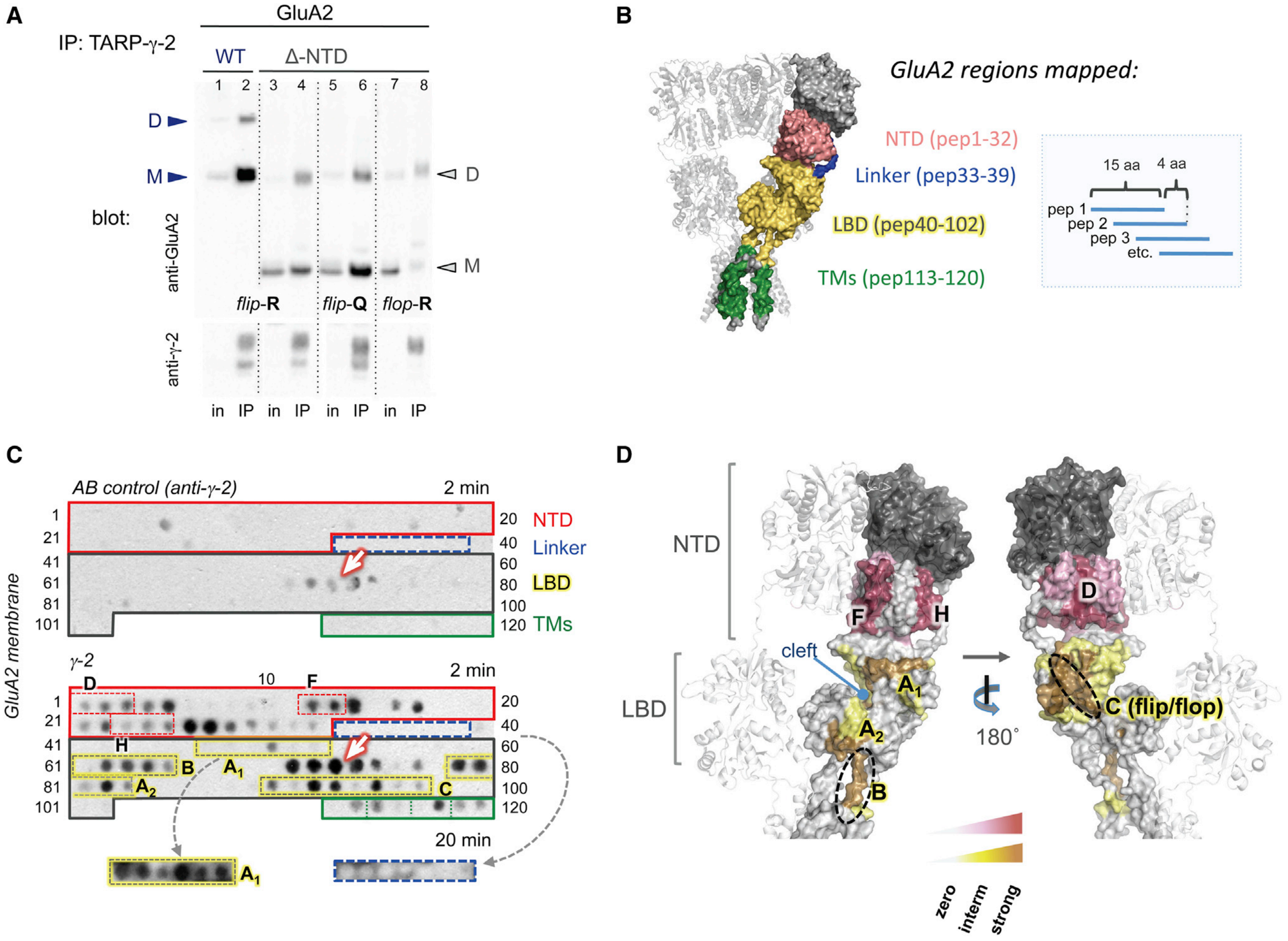

Figure 4. Mapping the TARP γ-2 Contact Region on GluA2

(A) coIP of GluA2 variants with TARP γ-2. The blot was probed with polyclonal GluA2 antibody (top panel) and anti γ-2 (bottom panel). Both WT and ΔNTD protein migrated as monomer (M) and dimer (D), denoted by arrowheads. Note that while inputs were comparable, amounts of IPed GluA2 varied between conditions.

(B) Schematic of the peptide array layout (right). Each peptide is spotted onto a nitrocellulose membrane (C). Peptide coverage of the rat GluA2 sequence is outlined in the left panel in color code as indicated. The four GluA2 regions—the NTD lower lobe, the NTD-LBD linkers, the LBD, and the TM segments of the channel—are highlighted. GluA2 peptide numbers covering each domain are indicated in brackets.

See Table S1 for peptide sequences.

(C) Regions of GluA2 binding to TARP γ-2. (Upper panel) Nonspecific signal, resulting from anti-γ-2 antibody binding to GluA2 in the absence of the γ-2 probe (“AB control”). AMPAR domains are highlighted in boxes and match the color scheme in (B). Peptide numbers are indicated on the side. The membrane was exposed to an X-ray film for 2 min; the arrow denotes nonspecific signals. (Lower panel) The same membrane was probed with full-length TARP γ-2 and detected with anti-γ-2 AB followed by a HRP-labeled secondary AB (2 min exposure). Individual AMPAR secondary structure elements, corresponding to NTD and LBD helices, are highlighted in stippled boxes on the blot (compared with D). The bottom panel shows a longer exposure for the LBD-A1 region (yellow) and the NTD-LBD linker (blue).

See Figure S4A for a longer exposure of the blot.

(D) TARP binding sites deduced from the peptide array in (C) are mapped onto the extracellular region of GluA2 (PDB: 3KG2). NTD interaction sites are denoted in deep red (strong interaction) and light pink (weaker interaction; see graded bar below), with alpha helices contacted by γ-2 denoted by (D), (F), and (H). LBD interaction sites are highlighted in brown (strong interaction) and yellow (weaker interaction). The three core contact regions, A–C, are denoted. Region A spans the glutamate binding cleft (interaction sites A_1 and A_2); region B encompasses the LBD-TM linker 1, and region C corresponds to the flip-flop cassette (denoted with a stippled ellipsoid).

See also Figures S3, S4, and S6 and Table S1.

helix H in the lower lobe (Figures 4D and S4D) and is thus ideally positioned to affect LBD clamshell motions associated with gating. Region B encompasses LBD-TMD linkers, which translate LBD motions into channel opening. Strong signals were apparent in the LBD-TM1 linker but not in LBD-TM4. The TM3 linker, which connects the LBD to the channel gate, is positioned in the “interior” of GluA2 (Sobolevsky et al., 2009) and may be less accessible. The transmembrane sector, which exhibits

OPEN ACCESS
Cell Press

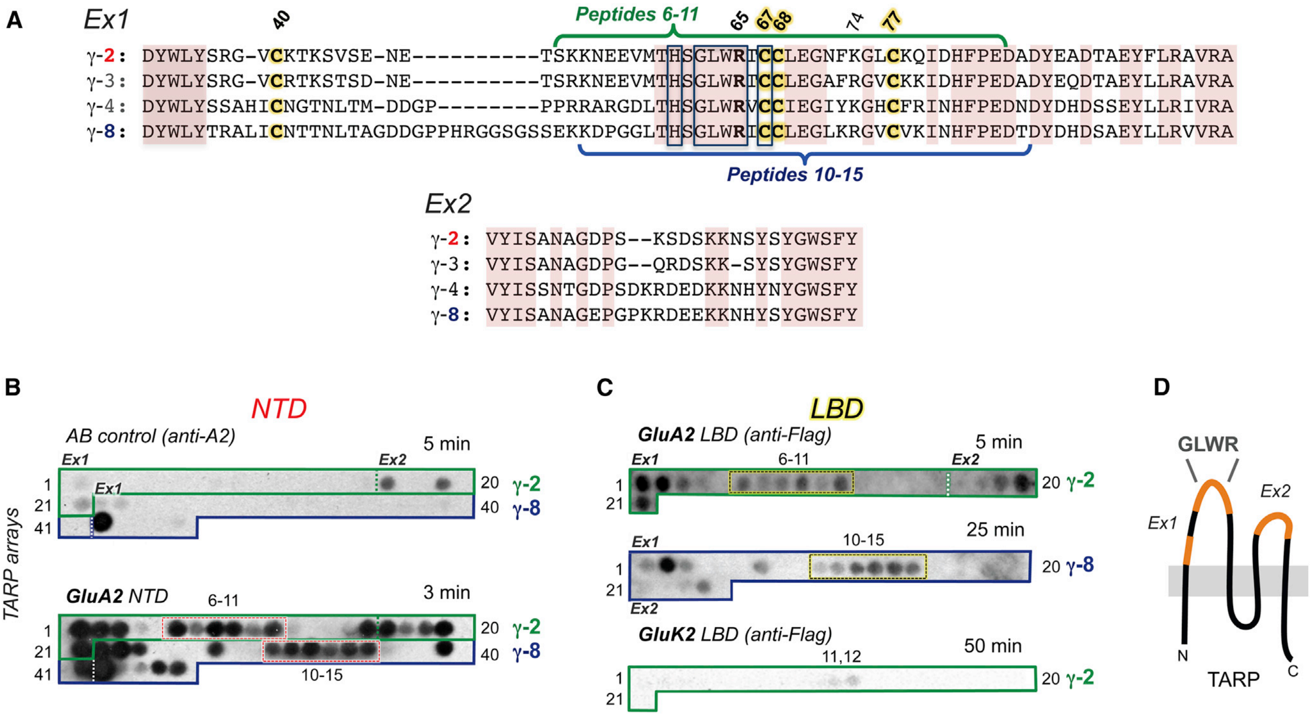

Figure 5. Mapping TARP γ-2 and TARP γ-8 Residues Involved in AMPAR Interaction

(A) Alignment of rat type 1a (γ-2, γ-3) and type 1b (γ-4, γ-8) TARP extracellular loops, Ex1 and Ex2. Conserved residues are shaded brown, and residues highly conserved throughout the Cacng family (γ-1 to γ-8) are boxed in gray. The four cysteines are highlighted in yellow. Curly brackets above the γ-2 (green) and below γ-8 alignment (blue) indicate regions in the center of Ex1 interacting with the AMPAR extracellular domains (NTD and LBD).

(B) TARP array encompassing the Ex1 and Ex2 segments of γ-2 (green box) and γ-8 (blue box) probed with the NTD. (Upper panel) Nonspecific signal, resulting from direct anti-GluA2 antibody binding to the membrane (AB control). The Ex1 and Ex2 regions for both TARPs are denoted (dashed line). (Lower panel) the same membrane was exposed to the rat GluA2 NTD followed by probing with anti-GluA2 AB. The membrane exposure time is as indicated.

See Table S2 for peptide sequences.

(C) γ-2 and γ-8 arrays probed with GluA2 LBD and GluK2 LBD. The negative controls with anti-FLAG AB did not show any signal and thus are not shown. Membranes were then incubated with FLAG-tagged GluA2 LBD or GluK2 LBD and probed with anti-FLAG AB. (Upper panel) GluA2 LBD interaction with γ-2 (same peptides as in Figure 5B, green box). (Central panel) GluA2 LBD interaction with γ-8 (same peptides as in B, blue box). (Lower panel) the γ-2 membrane previously probed with GluA2 LBD (top panel) was regenerated and probed with the FLAG-tagged GluK2 LBD, which produced no clear binding. Regeneration of this membrane resulted in a clear binding pattern when reprobed with the GluA2 LBD that matched the one shown in (C, top).

(D) Schematic representation of TARP structure with the GluA2 NTD and LBD interacting parts of the Ex1 and Ex2 loops highlighted in orange and the highly conserved GLWR motif indicated.

prominent swelling in TARP-associated AMPARs (Nakagawa et al., 2005), also showed signs of interaction. However, this region also exhibited nonspecific antibody binding, so we cannot make any specific assignment at present (Figure 4C). Similarly, strong background signals were observed along LBD helices F and G.

Region C encompasses the flip/flop cassette (helices J and K), which also contributed to complex stability in our coIP experiments (Figure 4A). These contacts likely account for the flip/flop differences in TARP modulation (Kott et al., 2007; Turetsky et al., 2005) and the altered specificity of AMPAR modulators in the presence of TARPs (Tomita et al., 2006). As is apparent in Figure 4D, this region follows a continuous path toward the interaction patch on the back of the NTD (NTD helix D; Figure 4D, right), which might tether the LBD to the NTD via the TARP. Taken together, these results provide a glimpse into the γ-2 contact points and reveal the functionally critical regions of the AMPAR interacting with γ-2.

Delineating GluA2 Binding Sites on TARPs γ-2 and γ-8

Next, we used an array of TARP peptides to identify TARP residues that contact the AMPAR (Table S2). We examined loops Ex1 and Ex2 (Figure 5A) in both type 1a (γ-2) and type 1b (γ-8) TARPs and mapped sites contacted by the NTD and LBD. Extensive contacts were indeed apparent on both γ-2 and γ-8 when we probed the array with the NTD (Figure 5B, lower panel). Only background signals were obtained when we omitted the NTD and tested the membrane with the antibody alone (upper panel). In addition to identifying NTD binding sites on Ex1, we also detected signals on the smaller Ex2 loop, which is only ~30 residues in length and thus not expected to protrude far above the plane of the plasma membrane. Moreover, in Ex1, the membrane-proximal N and C termini exhibited regions of NTD interaction. In vivo, these interactions would require substantial reconfigurations of the receptor, with the NTD reaching down toward the membrane (Figure S6B); NTD reconfigurations have been observed in

low-resolution structures of native AMPARs (Nakagawa et al., 2005). A prominent interaction region was also present in the center of Ex1, surrounding the highly conserved GLWRxC$_{67}$ motif present throughout the vertebrate Cacng family (γ-1 to γ-8) (Figures 5A, 5B, and 5D).

The same peptides were also probed with a Flag-tagged GluA2 LBD. Surprisingly, the LBD interaction sites on the TARPs γ-2 and γ-8 largely overlapped with those for the NTD. There were two noticeable differences: (1) the relative weight of signals across the Ex1 tip region, surrounding the double cysteine motif, varied between the LBD and NTD and (2) within Ex1, LBD binding to the N-terminal end of the loop was greater, whereas interaction with the tip region of the loop was reduced (Figure 5C). This indicates that the LBD interacts strongly with the membrane proximal region of Ex1, whereas the NTD binds more intimately to the Ex1 segment that surrounds the double cysteine motif (CC67, CC68).

The similarity between the NTD and LBD binding pattern prompted us to probe the specificity of this interaction further. As the related kainate receptors do not interact with TARPs (Chen et al., 2003), we probed the γ-2 array with a Flag-tagged GluK2 LBD (the GluA2 and GluK2 LBDs share only ~50% sequence identity) and did not observe clear binding (Figure 5C, bottom panel), suggesting that the observed AMPAR/γ-2 interaction profile is genuine. Together, these results corroborate an interaction of the NTD with type 1a and type 1b TARPs and reveal the sites on the TARPs involved in modulating AMPARs.

Interaction Regions in the γ-2 Ex1 Loop Are Critical for TARP Function

To test the functional relevance of the identified binding region (Figure 5D), we mutated the γ-2 Ex1 segment contacting the AMPAR and examined the effects on GluA2i currents. As shown in Figure 6A (top), triple and quadruple mutations were introduced into the tip region of Ex1. We tested the ability of TARP mutants to modulate channel kinetics, kainate efficacy (Figure 6), inward rectification, and channel conductance (Figure S5) (Jackson et al., 2011; Soto et al., 2007). We found that although all three γ-2 mutants retained some TARP-like functions, various channel parameters were affected differently and to varying degrees. For example, with the KGL$_{74–76}$ mutation, desensitization was faster than with γ-2 WT (Figure 6A), whereas kainate efficacy was unchanged relative to γ-2 WT (Figure 6D). On the other hand, GluA2i coexpressed with KQID$_{78–81}$ exhibited significantly lower kainate efficacy than did receptors expressed with γ-2 WT, while desensitization kinetics were comparable. The reduced kainate efficacy seen with KQID$_{78–81}$ and WRT$_{64–66}$ when compared with γ-2 WT suggests that these mutants disrupt TARP-AMPAR interactions in a way that might affect the degree of the LBD cleft closure, as this is known to determine the efficacy of partial agonists (Jin et al., 2003).

All γ-2 Ex1 mutants increased the weighted-mean channel conductance of GluA2i to the same extent as γ-2 WT (Figure S5). By contrast, the WRT$_{64–66}$ mutant produced less relief of polyamine block than did γ-2 WT (Figure S5), where the effect of the mutation was evident only at positive potentials (data not shown). These observations suggest that regions of the TARP distinct from Ex1, such as TM2 may play a role in modulating AMPAR properties, particularly those related to ion permeation, which likely result from interactions close to the channel pore.

Among the Ex1 mutants examined here, the WRT$_{64–66}$ mutation had the most profound impact on a variety of functional properties (Figures 6 and S5). WRT$_{64–66}$ was expressed at lower levels (~50%) than γ-2 WT; however, this mutant was targeted to the cell surface and biotinylation experiments revealed that the proportion of surface-expressed versus internal WRT$_{64–66}$ was comparable to the other γ-2 mutants (data not shown). In summary, our results identify functional hotspots in the γ-2 Ex1 loop and imply the existence of regions on the TARP that selectively influence different aspects of the AMPAR gating spectrum.

DISCUSSION

In this study, we report a role for the NTD (and the NTD-LBD linker) in AMPAR modulation by TARPs. We show that the NTD has the capacity to interact with TARPs and that selective shortening of the NTD linker can potentiate the modulatory function of TARPs in a TARP-selective fashion. Using peptide arrays, we identify NTD and LBD segments contacting γ-2 (Figure S6A), shedding light on the mechanisms underlying TARP modulation. In addition, we characterize contact points of the NTD and LBD on TARPs γ-2 and γ-8 and identify functional hotspots in the γ-2 interaction region. Our results imply that AMPARs are highly dynamic and may substantially reconfigure when interacting with auxiliary subunits (Figure S6B). We hypothesize that the flexible, modular organization of the AMPAR extracellular region permits selective interaction with other synaptic components, which may impact allosteric regulation of AMPARs.

The AMPAR Extracellular Region Is Flexible

The extracellular region of AMPA (and kainate) receptors constitutes ~80% of the mass of the receptor. The LBD layer is wedged between the ion channel and the NTD and is connected to both domains via peptide linkers. This flexible attachment, together with weak contacts within the LBD layer, is intimately linked to receptor gating, which requires substantial reconfigurations. In addition to the well-studied intradimer rearrangements associated with AMPAR desensitization (Armstrong et al., 2006; Sun et al., 2002), recent data from GluK2 kainate receptors reveal complete separation of the four LBDs upon desensitization (Schauder et al., 2013). This loose architecture permits large rearrangements that are required for AMPAR gating on the millisecond time scale (Plested and Mayer, 2009).

Subunit interactions within the distal NTD layer are substantially tighter as NTD dimers exhibit low nanomolar to low micromolar affinities (Herguedas et al., 2013; Rossmann et al., 2011; Zhao et al., 2012). These dimers associate as tetramers through a relatively small interface (Clayton et al., 2009; Jin et al., 2009; Kumar et al., 2009; Sobolevsky et al., 2009), which appears to be contacted by TARPs (Figure S4B). TARP association could therefore impact the organization of the distal layer, perhaps in a state-dependent fashion. Our peptide array data also imply that interdomain interactions between the NTD and LBD are altered by TARPs, which may underlie changes in desensitization rates observed in response to linker truncation.

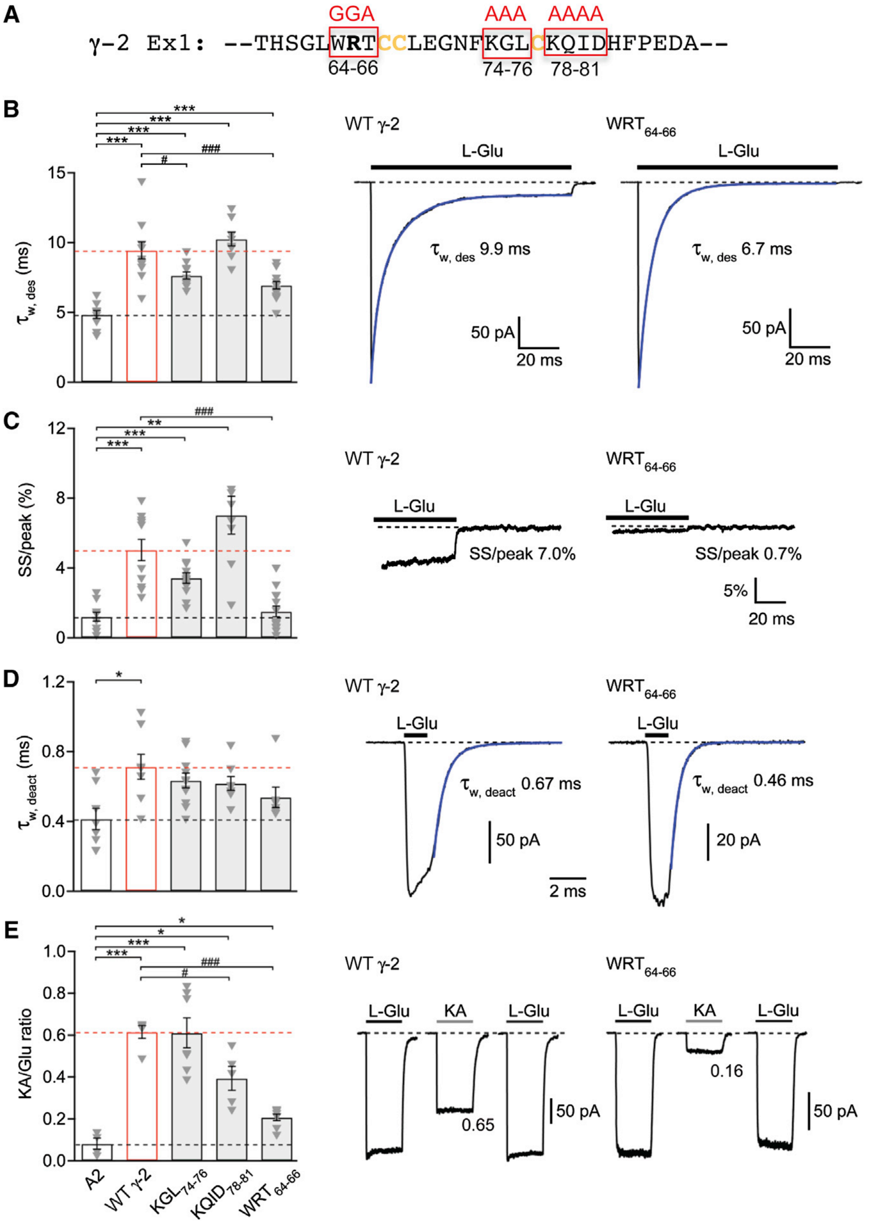

Figure 6. Effects of Ex1 Mutations in TARP γ-2

(A) Sequence of the γ-2 Ex1 region surrounding the highly conserved $GLWRxC_{67}$ motif. Boxed regions in red identify the amino acid triplets and quadruplet mutated in these experiments.

(B) Pooled data (mean ± SEM) showing the effects of Ex1 mutations in γ-2 on desensitization ($\tau_{w,des}$) of GluA2. Following one-way ANOVA ($F_{4,\ 23.27} = 28.89$, $p = 1.01 \times 10^{-8}$), pairwise comparisons showed that γ-2 WT (n = 12) and all three γ-2 mutants (KGL_{74-76}, $KQID_{78-81}$, and WRT_{64-66}; n = 12, 8, and 14, respectively) slowed $\tau_{w,des}$ compared with GluA2 alone (n = 10) (***$p < 0.001$) and that the effects of KGL_{74-76} and WRT_{64-66} were less than those of γ-2 WT ($^{\#}p < 0.05$, $^{\#\#\#}p < 0.001$) (Welch t tests). To the right are representative currents evoked by 10 mM L-glutamate (−60 mV) in patches from cells expressing GluA2/γ-2 WT and GluA2/γ-2 WRT_{64-66}. Also shown are the individual $\tau_{w,des}$ values determined from double exponential fits (blue).

(C) Pooled data showing the effects of mutations in γ-2 on the steady-state/peak ratio (SS/peak). Presentation, analysis, and n numbers as in B ($F_{4,\ 22.91} = 18.26$, $p = 7.27 \times 10^{-7}$; **$p < 0.01$ and ***$p < 0.001$ compared with GluA2 alone and $^{\#\#\#}p < 0.001$ compared with γ-2 WT). To the right are representative records from GluA2/γ-2 WT and GluA2/γ-2 WRT_{64-66} illustrating the current remaining at the end of the 100 ms applications of L-glutamate (10 mM) and the calculated SS/peak ratio.

(D) Pooled data (±SEM) showing the effects of mutations in γ-2 on GluA2 deactivation ($\tau_{w,deact}$; 1 ms, 10 mM L-glutamate, −60 mV). Following one-way ANOVA ($F_{4,\ 17.47} = 3.06$, $p = 0.044$), pairwise comparisons showed that only for γ-2 WT (n = 8) was $\tau_{w,deact}$ slowed compared with GluA2 alone (n = 8) (*$p < 0.05$). None of the mutants (KGL_{74-76}, $KQID_{78-81}$, and WRT_{64-66}; n = 11, 8, and 7, respectively) differed from GluA2 alone or GluA2/γ-2 WT. To the right are representative currents in patches taken from cells expressing GluA2/γ-2 WT and GluA2/γ-2 WRT_{64-66}. Also shown are the individual $\tau_{w,deact}$ values determined from double exponential fits (blue).

(E) Pooled data (± SEM) showing the effects of mutations in γ-2 on the KA/Glu ratio. Following one-way ANOVA ($F_{4,\ 9.85} = 44.8$, $p = 2.72 \times 10^{-6}$), pairwise comparisons showed that γ-2 WT (n = 5) and all three γ-2 mutants (KGL_{74-76}, $KQID_{78-81}$, and WRT_{64-66}; n = 7, 5, and 8, respectively) increased kainate efficacy (KA/Glu ratio) compared with that seen with GluA2 alone (n = 4) (*$p < 0.05$, ***$p < 0.001$) and that $KQID_{78-81}$ and WRT_{64-66} had less effect than γ-2 WT ($^{\#}p < 0.05$, $^{\#\#\#}p < 0.001$) (Welch t tests). To the right are representative currents evoked by L-glutamate and kainate (both 500 μM, in the presence of 100 μM cyclothiazide) in patches from cells expressing GluA2/γ-2 WT and GluA2/γ-2 WRT_{64-66}. Also shown are the KA/Glu ratios for these representative records.
See also Figure S5.

As TARPs are not expected to protrude far beyond the plane of the membrane (Suzuki et al., 2014), a direct contact between TARPs and the NTD would require substantial rearrangements of the receptor. Structural data lend some support to this hypothesis (Nakagawa et al., 2005). Since interactions within a membrane-embedded receptor complex likely differ from those in a peptide array probed with isolated (and therefore unconstrained) receptor domains, not all interactions described here may occur at the same time and may also depend on the functional state of the receptor. Related to this, TARP contacts may differ between the two nonequivalent AMPAR subunits pairs (*AC* vs. *BD*; Sobolevsky et al., 2009).

TARPs Interact with Functionally Critical AMPAR Regions

Within the LBD, TARP contacts include a number of sites of functional importance. Interactions across the LBD cleft are well suited to affect kainate efficacy, the pharmacology of

competitive antagonists, and the open-to-closed equilibrium of the clamshell (Cho et al., 2007; Milstein et al., 2007). Similarly, the LBD-TM1 linkers, which are contacted by γ-2, are involved in transmitting gating motions from the LBD to the ion channel and are thus well suited to shape gating. Curiously, TARPs contact the alternatively spliced flip/flop segment, which, according to our IP data, impacts the stability of the AMPAR/TARP complex. Our coIP results imply multiple regulatory sites on the receptor, the Q/R site in the pore, and the flip/flop cassette in the LBD, which in combination would determine AMPAR affinity for the TARP. This result highlights the strategic role of flip/flop splicing in AMPAR regulation (Coleman et al., 2006; Penn et al., 2012; Sommer et al., 1990). Somewhat unexpectedly, the GluA2i-ΔNTD mutant retained modulation by γ-2 to comparable levels as GluA2i WT (Figures S2C–S2E). Interestingly, earlier work showed that while GluA2i lacking the NTD was potentiated by γ-2, the GluA2o-ΔNTD mutant was not (Bedoukian et al., 2006). This may well be explained by the reduced TARP affinity seen with the flop variety in our coIPs and further indicates that multiple binding sites on the receptor (Figure S6A) contribute to elicit optimal TARP modulation.

Surprisingly, on the TARP itself, the GluA2 LBD and NTD contacted comparable regions in the extracellular loops; however, the relative weights of signal intensities were distinct. This pattern of interaction is unexpected and prevented us from selectively mutating residues within LBD versus NTD interaction regions. Structural data are required to resolve the precise organization of AMPAR TARP complex. Another curious feature of our results is the close similarity between γ-2 and γ-8 in their interactions with AMPAR. These two TARPs are of strikingly different length and share only ~48% sequence similarity in their extracellular regions. As signals across Ex1 centered around the four cysteines, tertiary structural features of the loop that result from disulphide bonding (Suzuki et al., 2014) are likely to underlie this interaction. The functional consequence of the NTD-γ8 interaction remains to be established.

Functional Implications of TARP-Induced AMPAR Rearrangements

A continuous path of TARP interaction, extending beyond the flip/flop region toward the back of the NTD, may permit "bridging" between the NTD and LBD. The resulting compact arrangement between these two domains may "incorporate" the otherwise loosely connected NTD layer into an allosteric unit with the LBD (Figure S6B). The AMPAR rearrangement, triggered by TARPs, reflects a capacity of the AMPAR for dynamic reorganization that might permit interactions with other synaptic components, such as cadherins and pentraxins (Saglietti et al., 2007; Sia et al., 2007), to impact postsynaptic response properties via the NTD.

The sequence diversity of the NTD, combined with the existence of multiple TARPs heterogeneously expressed in diverse neuronal populations, may provide further capacity for differential regulation of AMPAR subtypes. First, the NTD-TARP contact region described in this study may offer a target for the development of novel AMPAR-subtype selective drugs (Gill and Bredt, 2011). Second, AMPARs have been suggested to dissociate from TARPs upon activation by L-glutamate, prior to endocytosis (Tomita et al., 2004). Thus, AMPAR subunit combinations with different affinities for TARPs, mediated via the sequence-diverse NTD, could exhibit distinct endocytosis rates and lateral diffusion, influencing the dwell-time of AMPARs at postsynaptic sites (Bredt and Nicoll, 2003; Opazo et al., 2012). Therefore, the extracellular region of AMPARs might turn out to be a key element for regulating functional and structural plasticity at excitatory synapses.

EXPERIMENTAL PROCEDURES

Additional details are provided in the Supplemental Experimental Procedures.

Protein Production

His-tagged γ-2 was produced in insect cells using a P1 baculovirus stock following a purification protocol provided by T. Nakagawa. High-titer viral stocks were obtained following the Bac-N-Blue protocol (Invitrogen). Protein was solubilized with decyl-maltoside and purified by Cobalt-affinity chromatography. The GluA2 NTD was purified from stably transfected GntI$^-$ HEK293S cells (Rossmann et al., 2011). A FLAG-tag was introduced at the GluA2i LBD C terminus (R/Flip; (Armstrong and Gouaux, 2000; Greger et al., 2006) and the GluK2 LBD (provided by M. Mayer) and cloned into a pET22b(+) plasmid containing an N-terminal His_8 tag and a thrombin cleavage site. Proteins were produced in Origami B (DE3) cells and purified on a HisTrap HP column followed by thrombin cleavage and gel filtration.

Peptide Arrays

The interaction between GluA2 and TARPs γ-2 and γ-8 was mapped with peptide arrays synthesized by SPOT synthesis (PepSpots from JPT Peptide Tech.). AMPAR and TARP arrays contained 15-mer overlapping peptides shifted by four residues (Tables S1 and S2). The GluA2 array was probed with full-length γ-2, whereas the TARP γ-2 and γ-8 arrays were probed with GluA2 NTD, GluA2 LBD, or GluK2 LBD, following the manufacturer's protocol. Prior to exposing the arrays to specific protein probes, the membranes were incubated with antibodies only to determine nonspecific binding. Membranes were blocked with 5% BSA and incubated with primary antibodies: anti-FLAG (monoclonal; Sigma-Aldrich), anti-Stargazin (polyclonal; Millipore), or anti-GluA2 (polyclonal; Alomone). After incubation with HRP-coupled secondary antibodies (Pierce), membranes were developed with enhanced chemiluminescence and images were captured electronically with a ChemiDoc MP Imaging System (Biorad) or on an X-ray film.

Electrophysiology

Voltage-clamp recordings of rat GluA2i (flip, R/G-edited, Q/R-unedited) or GluA3i (flip, R/G-edited and containing the R463G point mutation for increased surface expression; Coleman et al., 2010) were performed as described previously (Rossmann et al., 2011; Soto et al., 2007). Briefly, outside-out patches were pulled from HEK293(T) cells transfected with rat GluA2i or GluA3i, and current responses to rapid application of 10 mM L-glutamate via a θ tube were recorded. Where indicated, TARPs were coexpressed, either transiently cotransfected or using a cell line stably expressing γ-2 or γ-8. The kinetics of the receptor desensitization, deactivation, and recovery from desensitization were analyzed by fitting currents with single- or double-exponential functions. In addition, steady state-to-peak ratio, relative kainate efficacy, rectification, and single channel properties (by nonstationary fluctuation analysis) were assessed.

Statistics

Summary data are presented as the mean ± SEM from *n* patches. Comparisons involving two data sets only were performed using a two-sided Welch two-sample t test. All analyses involving data from three or more groups were performed using one- or two-way analysis of variance (Welch heteroscedastic F test) followed by pairwise comparisons using two-sided Welch two-sample t tests (with Holm's sequential Bonferroni correction for multiple comparisons). Differences were considered significant at $p < 0.05$. Statistical tests were performed using Prism 4.0/6.0 (GraphPad Software) or R (v.3.0.2, The R Foundation for Statistical Computing, http://www.r-project.org/) and RStudio (v.0.98.313, RStudio).

SUPPLEMENTAL INFORMATION

Supplemental Information includes Supplemental Experimental Procedures, six figures, and two tables and can be found with this article online at http://dx.doi.org/10.1016/j.celrep.2014.09.029.

AUTHOR CONTRIBUTIONS

I.H.G., M.F., and S.G.C.-C. designed and interpreted experiments and wrote the manuscript. I.H.G., O.C., B.H., and K.K. performed experiments and contributed to design of experiments and writing. O.C., I.H.G., B.H., K.K., and M.F. analyzed data.

ACKNOWLEDGMENTS

We thank members of the I.H.G., M.F., and S.G.C.-C. laboratories and Andy Penn for critically reading of the manuscript. We acknowledge James Krieger for helpful comments. We thank Terunaga Nakagawa (Vanderbilt University) for providing the GluA2-FLAG plasmid, the TARP γ-2 Baculovirus construct, and the unpublished γ-2 expression protocol from insect cells. We thank Mark Mayer for the GluK2 LBD construct and D. Cutler and M. Marsh (Laboratory for Molecular Cell Biology, UCL) for generous help and access to equipment. This study was supported by the Medical Research Council (MC_U105174197 to I.H.G.; MR/J002976/1 to S.G.C.C. and M.F.; MR/J012998/1 to M.F. and S.G.C.-C.), the Wellcome Trust (086185/Z/08/Z to S.G.C.-C. and M.F.) and the Royal Society (I.H.G.). K.K. was in receipt of a Medical Research Council (Laboratory for Molecular Cell Biology, UCL) studentship.

Received: June 30, 2014
Revised: August 12, 2014
Accepted: September 19, 2014
Published: October 16, 2014

REFERENCES

Armstrong, N., and Gouaux, E. (2000). Mechanisms for activation and antagonism of an AMPA-sensitive glutamate receptor: crystal structures of the GluR2 ligand binding core. Neuron *28*, 165–181.

Armstrong, N., Jasti, J., Beich-Frandsen, M., and Gouaux, E. (2006). Measurement of conformational changes accompanying desensitization in an ionotropic glutamate receptor. Cell *127*, 85–97.

Bedoukian, M.A., Weeks, A.M., and Partin, K.M. (2006). Different domains of the AMPA receptor direct stargazin-mediated trafficking and stargazin-mediated modulation of kinetics. J. Biol. Chem. *281*, 23908–23921.

Bredt, D.S., and Nicoll, R.A. (2003). AMPA receptor trafficking at excitatory synapses. Neuron *40*, 361–379.

Chen, L., El-Husseini, A., Tomita, S., Bredt, D.S., and Nicoll, R.A. (2003). Stargazin differentially controls the trafficking of alpha-amino-3-hydroxyl-5-methyl-4-isoxazolepropionate and kainate receptors. Mol. Pharmacol. *64*, 703–706.

Cho, C.H., St-Gelais, F., Zhang, W., Tomita, S., and Howe, J.R. (2007). Two families of TARP isoforms that have distinct effects on the kinetic properties of AMPA receptors and synaptic currents. Neuron *55*, 890–904.

Clayton, A., Siebold, C., Gilbert, R.J., Sutton, G.C., Harlos, K., McIlhinney, R.A., Jones, E.Y., and Aricescu, A.R. (2009). Crystal structure of the GluR2 amino-terminal domain provides insights into the architecture and assembly of ionotropic glutamate receptors. J. Mol. Biol. *392*, 1125–1132.

Coleman, S.K., Möykkynen, T., Cai, C., von Ossowski, L., Kuismanen, E., Korpi, E.R., and Keinänen, K. (2006). Isoform-specific early trafficking of AMPA receptor flip and flop variants. J. Neurosci. *26*, 11220–11229.

Coleman, S.K., Möykkynen, T., Hinkkuri, S., Vaahtera, L., Korpi, E.R., Pentikäinen, O.T., and Keinänen, K. (2010). Ligand-binding domain determines endoplasmic reticulum exit of AMPA receptors. J. Biol. Chem. *285*, 36032–36039.

Cull-Candy, S., Kelly, L., and Farrant, M. (2006). Regulation of Ca2+-permeable AMPA receptors: synaptic plasticity and beyond. Curr. Opin. Neurobiol. *16*, 288–297.

Dutta, A., Shrivastava, I.H., Sukumaran, M., Greger, I.H., and Bahar, I. (2012). Comparative dynamics of NMDA- and AMPA-glutamate receptor N-terminal domains. Structure *20*, 1838–1849.

Geiger, J.R., Melcher, T., Koh, D.S., Sakmann, B., Seeburg, P.H., Jonas, P., and Monyer, H. (1995). Relative abundance of subunit mRNAs determines gating and Ca2+ permeability of AMPA receptors in principal neurons and interneurons in rat CNS. Neuron *15*, 193–204.

Gielen, M., Siegler Retchless, B., Mony, L., Johnson, J.W., and Paoletti, P. (2009). Mechanism of differential control of NMDA receptor activity by NR2 subunits. Nature *459*, 703–707.

Gill, M.B., and Bredt, D.S. (2011). An emerging role for TARPs in neuropsychiatric disorders. Neuropsychopharmacology *36*, 362–363.

Gill, M.B., Kato, A.S., Wang, H., and Bredt, D.S. (2012). AMPA receptor modulation by cornichon-2 dictated by transmembrane AMPA receptor regulatory protein isoform. Eur. J. Neurosci. *35*, 182–194.

Greger, I.H., Akamine, P., Khatri, L., and Ziff, E.B. (2006). Developmentally regulated, combinatorial RNA processing modulates AMPA receptor biogenesis. Neuron *51*, 85–97.

Hansen, K.B., Furukawa, H., and Traynelis, S.F. (2010). Control of assembly and function of glutamate receptors by the amino-terminal domain. Mol. Pharmacol. *78*, 535–549.

Herguedas, B., Krieger, J., and Greger, I.H. (2013). Receptor heteromeric assembly-how it works and why it matters: the case of ionotropic glutamate receptors. Prog. Mol. Biol. Transl. Sci. *117*, 361–386.

Jackson, A.C., and Nicoll, R.A. (2011). The expanding social network of ionotropic glutamate receptors: TARPs and other transmembrane auxiliary subunits. Neuron *70*, 178–199.

Jackson, A.C., Milstein, A.D., Soto, D., Farrant, M., Cull-Candy, S.G., and Nicoll, R.A. (2011). Probing TARP modulation of AMPA receptor conductance with polyamine toxins. J. Neurosci. *31*, 7511–7520.

Jensen, M.H., Sukumaran, M., Johnson, C.M., Greger, I.H., and Neuweiler, H. (2011). Intrinsic motions in the N-terminal domain of an ionotropic glutamate receptor detected by fluorescence correlation spectroscopy. J. Mol. Biol. *414*, 96–105.

Jin, R., Banke, T.G., Mayer, M.L., Traynelis, S.F., and Gouaux, E. (2003). Structural basis for partial agonist action at ionotropic glutamate receptors. Nat. Neurosci. *6*, 803–810.

Jin, R., Singh, S.K., Gu, S., Furukawa, H., Sobolevsky, A.I., Zhou, J., Jin, Y., and Gouaux, E. (2009). Crystal structure and association behaviour of the GluR2 amino-terminal domain. EMBO J. *28*, 1812–1823.

Jonas, P. (2000). The time course of signaling at central glutamatergic synapses. News Physiol. Sci. *15*, 83–89.

Kato, A.S., Gill, M.B., Yu, H., Nisenbaum, E.S., and Bredt, D.S. (2010). TARPs differentially decorate AMPA receptors to specify neuropharmacology. Trends Neurosci. *33*, 241–248.

Katz, C., Levy-Beladev, L., Rotem-Bamberger, S., Rito, T., Rüdiger, S.G., and Friedler, A. (2011). Studying protein-protein interactions using peptide arrays. Chem. Soc. Rev. *40*, 2131–2145.

Kott, S., Werner, M., Körber, C., and Hollmann, M. (2007). Electrophysiological properties of AMPA receptors are differentially modulated depending on the associated member of the TARP family. J. Neurosci. *27*, 3780–3789.

Kumar, J., and Mayer, M.L. (2013). Functional insights from glutamate receptor ion channel structures. Annu. Rev. Physiol. *75*, 313–337.

Kumar, J., Schuck, P., Jin, R., and Mayer, M.L. (2009). The N-terminal domain of GluR6-subtype glutamate receptor ion channels. Nat. Struct. Mol. Biol. *16*, 631–638.

Ma, B., Tsai, C.J., Haliloğlu, T., and Nussinov, R. (2011). Dynamic allostery: linkers are not merely flexible. Structure *19*, 907–917.

Milstein, A.D., Zhou, W., Karimzadegan, S., Bredt, D.S., and Nicoll, R.A. (2007). TARP subtypes differentially and dose-dependently control synaptic AMPA receptor gating. Neuron *55*, 905–918.

Mony, L., Kew, J.N., Gunthorpe, M.J., and Paoletti, P. (2009). Allosteric modulators of NR2B-containing NMDA receptors: molecular mechanisms and therapeutic potential. Br. J. Pharmacol. *157*, 1301–1317.

Mony, L., Zhu, S., Carvalho, S., and Paoletti, P. (2011). Molecular basis of positive allosteric modulation of GluN2B NMDA receptors by polyamines. EMBO J. *30*, 3134–3146.

Morimoto-Tomita, M., Zhang, W., Straub, C., Cho, C.H., Kim, K.S., Howe, J.R., and Tomita, S. (2009). Autoinactivation of neuronal AMPA receptors via glutamate-regulated TARP interaction. Neuron *61*, 101–112.

Mosbacher, J., Schoepfer, R., Monyer, H., Burnashev, N., Seeburg, P.H., and Ruppersberg, J.P. (1994). A molecular determinant for submillisecond desensitization in glutamate receptors. Science *266*, 1059–1062.

Möykkynen, T., Coleman, S.K., Semenov, A., and Keinänen, K. (2014). The N-terminal domain modulates α-amino-3-hydroxy-5-methyl-4-isoxazolepropionic acid (AMPA) receptor desensitization. J. Biol. Chem. *289*, 13197–13205.

Nakagawa, T., Cheng, Y., Ramm, E., Sheng, M., and Walz, T. (2005). Structure and different conformational states of native AMPA receptor complexes. Nature *433*, 545–549.

Nakagawa, T., Cheng, Y., Sheng, M., and Walz, T. (2006). Three-dimensional structure of an AMPA receptor without associated stargazin/TARP proteins. Biol. Chem. *387*, 179–187.

Opazo, P., Sainlos, M., and Choquet, D. (2012). Regulation of AMPA receptor surface diffusion by PSD-95 slots. Curr. Opin. Neurobiol. *22*, 453–460.

Paoletti, P. (2011). Molecular basis of NMDA receptor functional diversity. Eur. J. Neurosci. *33*, 1351–1365.

Pasternack, A., Coleman, S.K., Jouppila, A., Mottershead, D.G., Lindfors, M., Pasternack, M., and Keinänen, K. (2002). Alpha-amino-3-hydroxy-5-methyl-4-isoxazolepropionic acid (AMPA) receptor channels lacking the N-terminal domain. J. Biol. Chem. *277*, 49662–49667.

Penn, A.C., Balik, A., Wozny, C., Cais, O., and Greger, I.H. (2012). Activity-mediated AMPA receptor remodeling, driven by alternative splicing in the ligand-binding domain. Neuron *76*, 503–510.

Plested, A.J., and Mayer, M.L. (2009). AMPA receptor ligand binding domain mobility revealed by functional cross linking. J. Neurosci. *29*, 11912–11923.

Priel, A., Kolleker, A., Ayalon, G., Gillor, M., Osten, P., and Stern-Bach, Y. (2005). Stargazin reduces desensitization and slows deactivation of the AMPA-type glutamate receptors. J. Neurosci. *25*, 2682–2686.

Rossmann, M., Sukumaran, M., Penn, A.C., Veprintsev, D.B., Babu, M.M., and Greger, I.H. (2011). Subunit-selective N-terminal domain associations organize the formation of AMPA receptor heteromers. EMBO J. *30*, 959–971.

Saglietti, L., Dequidt, C., Kamieniarz, K., Rousset, M.C., Valnegri, P., Thoumine, O., Beretta, F., Fagni, L., Choquet, D., Sala, C., et al. (2007). Extracellular interactions between GluR2 and N-cadherin in spine regulation. Neuron *54*, 461–477.

Schauder, D.M., Kuybeda, O., Zhang, J., Klymko, K., Bartesaghi, A., Borgnia, M.J., Mayer, M.L., and Subramaniam, S. (2013). Glutamate receptor desensitization is mediated by changes in quaternary structure of the ligand binding domain. Proc. Natl. Acad. Sci. USA *110*, 5921–5926.

Schwenk, J., Harmel, N., Zolles, G., Bildl, W., Kulik, A., Heimrich, B., Chisaka, O., Jonas, P., Schulte, U., Fakler, B., and Klöcker, N. (2009). Functional proteomics identify cornichon proteins as auxiliary subunits of AMPA receptors. Science *323*, 1313–1319.

Schwenk, J., Harmel, N., Brechet, A., Zolles, G., Berkefeld, H., Müller, C.S., Bildl, W., Baehrens, D., Hüber, B., Kulik, A., et al. (2012). High-resolution proteomics unravel architecture and molecular diversity of native AMPA receptor complexes. Neuron *74*, 621–633.

Shanks, N.F., Savas, J.N., Maruo, T., Cais, O., Hirao, A., Oe, S., Ghosh, A., Noda, Y., Greger, I.H., Yates, J.R., 3rd, and Nakagawa, T. (2012). Differences in AMPA and kainate receptor interactomes facilitate identification of AMPA receptor auxiliary subunit GSG1L. Cell Rep *1*, 590–598.

Shanks, N.F., Cais, O., Maruo, T., Savas, J.N., Zaika, E.I., Azumaya, C.M., Yates, J.R., 3rd, Greger, I., and Nakagawa, T. (2014). Molecular Dissection of the Interaction between the AMPA Receptor and Cornichon Homolog-3. J. Neurosci. *34*, 12104–12120.

Sia, G.M., Béïque, J.C., Rumbaugh, G., Cho, R., Worley, P.F., and Huganir, R.L. (2007). Interaction of the N-terminal domain of the AMPA receptor GluR4 subunit with the neuronal pentraxin NP1 mediates GluR4 synaptic recruitment. Neuron *55*, 87–102.

Sobolevsky, A.I., Rosconi, M.P., and Gouaux, E. (2009). X-ray structure, symmetry and mechanism of an AMPA-subtype glutamate receptor. Nature *462*, 745–756.

Sommer, B., Keinänen, K., Verdoorn, T.A., Wisden, W., Burnashev, N., Herb, A., Köhler, M., Takagi, T., Sakmann, B., and Seeburg, P.H. (1990). Flip and flop: a cell-specific functional switch in glutamate-operated channels of the CNS. Science *249*, 1580–1585.

Soto, D., Coombs, I.D., Kelly, L., Farrant, M., and Cull-Candy, S.G. (2007). Stargazin attenuates intracellular polyamine block of calcium-permeable AMPA receptors. Nat. Neurosci. *10*, 1260–1267.

Sukumaran, M., Rossmann, M., Shrivastava, I., Dutta, A., Bahar, I., and Greger, I.H. (2011). Dynamics and allosteric potential of the AMPA receptor N-terminal domain. EMBO J. *30*, 972–982.

Sun, Y., Olson, R., Horning, M., Armstrong, N., Mayer, M., and Gouaux, E. (2002). Mechanism of glutamate receptor desensitization. Nature *417*, 245–253.

Suzuki, H., Nishizawa, T., Tani, K., Yamazaki, Y., Tamura, A., Ishitani, R., Dohmae, N., Tsukita, S., Nureki, O., and Fujiyoshi, Y. (2014). Crystal structure of a claudin provides insight into the architecture of tight junctions. Science *344*, 304–307.

Tomita, S., Fukata, M., Nicoll, R.A., and Bredt, D.S. (2004). Dynamic interaction of stargazin-like TARPs with cycling AMPA receptors at synapses. Science *303*, 1508–1511.

Tomita, S., Adesnik, H., Sekiguchi, M., Zhang, W., Wada, K., Howe, J.R., Nicoll, R.A., and Bredt, D.S. (2005). Stargazin modulates AMPA receptor gating and trafficking by distinct domains. Nature *435*, 1052–1058.

Tomita, S., Sekiguchi, M., Wada, K., Nicoll, R.A., and Bredt, D.S. (2006). Stargazin controls the pharmacology of AMPA receptor potentiators. Proc. Natl. Acad. Sci. USA *103*, 10064–10067.

Tomita, S., Shenoy, A., Fukata, Y., Nicoll, R.A., and Bredt, D.S. (2007). Stargazin interacts functionally with the AMPA receptor glutamate-binding module. Neuropharmacology *52*, 87–91.

Traynelis, S.F., Wollmuth, L.P., McBain, C.J., Menniti, F.S., Vance, K.M., Ogden, K.K., Hansen, K.B., Yuan, H., Myers, S.J., and Dingledine, R. (2010). Glutamate receptor ion channels: structure, regulation, and function. Pharmacol. Rev. *62*, 405–496.

Trussell, L. (1998). Control of time course of glutamatergic synaptic currents. Prog. Brain Res. *116*, 59–69.

Turetsky, D., Garringer, E., and Patneau, D.K. (2005). Stargazin modulates native AMPA receptor functional properties by two distinct mechanisms. J. Neurosci. *25*, 7438–7448.

von Engelhardt, J., Mack, V., Sprengel, R., Kavenstock, N., Li, K.W., Stern-Bach, Y., Smit, A.B., Seeburg, P.H., and Monyer, H. (2010). CKAMP44: a brain-specific protein attenuating short-term synaptic plasticity in the dentate gyrus. Science *327*, 1518–1522.

Walker, C.S., Brockie, P.J., Madsen, D.M., Francis, M.M., Zheng, Y., Koduri, S., Mellem, J.E., Strutz-Seebohm, N., and Maricq, A.V. (2006). Reconstitution of invertebrate glutamate receptor function depends on stargazin-like proteins. Proc. Natl. Acad. Sci. USA *103*, 10781–10786.

Wang, R., Walker, C.S., Brockie, P.J., Francis, M.M., Mellem, J.E., Madsen, D.M., and Maricq, A.V. (2008). Evolutionary conserved role for TARPs in the

gating of glutamate receptors and tuning of synaptic function. Neuron *59*, 997–1008.

Yuan, H., Hansen, K.B., Vance, K.M., Ogden, K.K., and Traynelis, S.F. (2009). Control of NMDA receptor function by the NR2 subunit amino-terminal domain. J. Neurosci. *29*, 12045–12058.

Zhao, H., Berger, A.J., Brown, P.H., Kumar, J., Balbo, A., May, C.A., Casillas, E., Jr., Laue, T.M., Patterson, G.H., Mayer, M.L., and Schuck, P. (2012). Analysis of high-affinity assembly for AMPA receptor amino-terminal domains. J. Gen. Physiol. *139*, 371–388.

Note Added in Proof

While this paper was under review, the two following structural studies provided additional evidence for the structural dynamics of the AMPAR NTD layer: Dürr, K.L., Chen, L., Stein, R.A., De Zorzi, R., Folea, I.M., Walz, T., Mchaourab, H.S., and Gouaux, E. (2014). Structure and dynamics of AMPA receptor GluA2 in resting, pre-open, and desensitized states. Cell *158*, 778–792.

Meyerson, J.R., Kumar, J., Chittori, S., Rao, P., Pierson, J., Bartesaghi, A., Mayer, M.L., and Subramaniam, S. (2014). Structural mechanism of glutamate receptor activation and desensitization. Nature. http://dx.doi.org/10.1038/nature13603.

Cell Reports, Volume *9*

Supplemental Information

Mapping the Interaction Sites between AMPA Receptors and TARPs Reveals a Role for the Receptor N-Terminal Domain in Channel Gating

Ondrej Cais, Beatriz Herguedas, Karolina Krol, Stuart G. Cull-Candy, Mark Farrant, and Ingo H. Greger

Supplemental Information

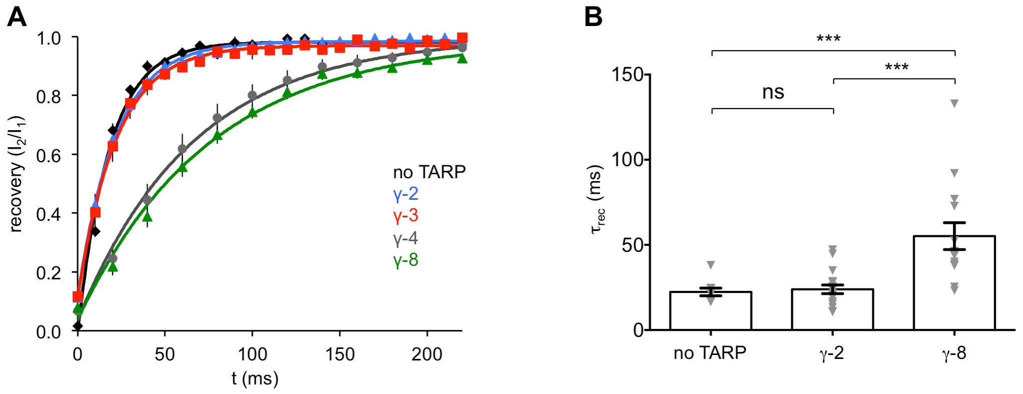

Figure S1. Differential modulation of GluA2 and GluA3 recovery from desensitization by Type-1a and -1b TARPs, related to Figure 2.

A. Graphical summary of recovery from desensitization of GluA2 wt expressed with different TARPs. Relative currents at individual time points are shown ± SEM (error bars masked by the symbols in most cases). The solid lines are monoexponential fits of the averages, giving similar time constants of 18.1 ms, 21.8 ms and 22.2 ms for GluA2 alone (n = 11), GluA2 with γ-2 (n = 17) and GluA2 with γ-3 (n = 6), respectively. Much slower recovery was seen for GluA2 with γ-4 (66.5 ms; n = 8) and GluA2 with γ-8 (75.6 ms; n = 7).

B. Pooled data (shown ± SEM) for the time constant of recovery from desensitization for GluA3i (R463G) expressed alone or with TARPs γ-2 or γ-8 (n = 9, 17 and 14, respectively) (*** $P < 0.001$; two-sided Welch two-sample t-tests with Holm's sequential Bonferroni correction for multiple comparisons).

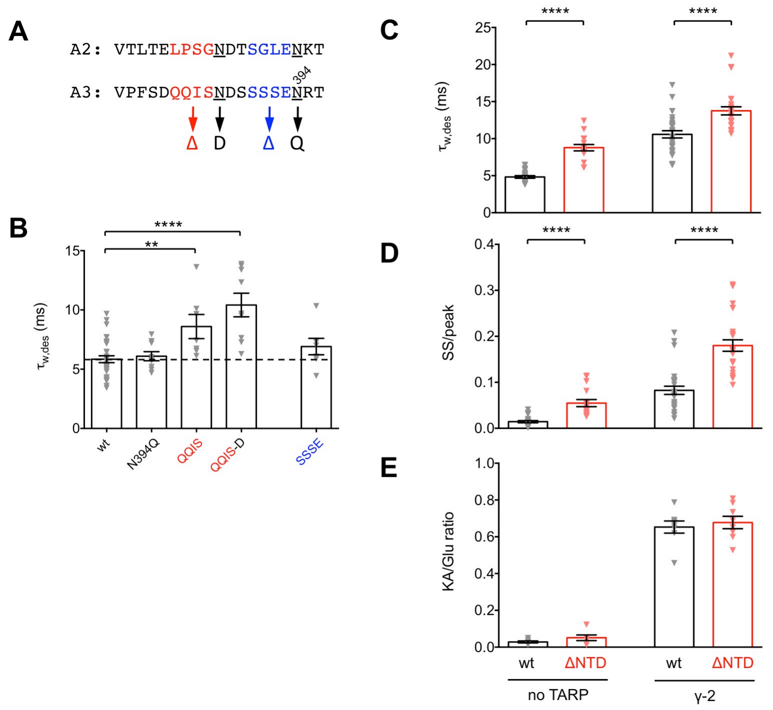

Figure S2. NTD-LBD linker mutations affect desensitization of GluA3, the effect of γ-2 TARP on GluA2 gating is not eliminated in the absence of NTD, related to Figure 3.

A. Sequence alignment of the NTD-LBD linkers of GluA2 and GluA3, with the deleted/mutated regions highlighted (GluA2 mutants described in Figure 3).

B. Pooled data (± SEM) showing the effect of NTD-LBD linker mutations on desensitization kinetics of GluA3 in the presence of TARP γ-2. QQIS denotes deletion of these four amino acids, QQIS-D combines this with the N387D point mutation, while SSSE is an alternative four-amino acid deletion. Values were compared using one-way ANOVA. Pairwise comparisons showed that $\tau_{w,des}$ was significantly slower for QQIS (*n* = 7) and QQIS-D (*n* = 9) compared to wt (*n* =31) (** $P < 0.01$, *** $P < 0.001$) (two-sided Welch two-sample *t* tests with Holm's sequential

Bonferroni correction for multiple comparisons). There was no significant effect of the glycosylation mutation N394Q (*n* = 9) or the alternative deletion mutant, SSSE (*n* = 7).

C. Pooled data (± SEM) showing the effect of NTD deletion on desensitization kinetics of GluA2i. $\tau_{w,des}$ was significantly increased both in the absence (n = 23 and 16 for GluA2 wt and ΔNTD, respectively) and presence (n = 29 and 25) of TARP γ-2 (**** $P < 0.0001$; two-sided Welch two-sample *t* test). Two-way ANOVA showed no significant interaction between NTD and γ-2 presence ($F_{1,\ 89} = 0.69$, $P = 0.4084$).

D. Pooled data for the steady state-to-peak ratio, presented and analysed as in C. SS/Peak was significantly higher for the ΔNTD both in the absence (n = 21 and 16 for GluA2 wt and ΔNTD, respectively) and presence (n = 28 and 25) of γ-2 (**** $P < 0.0001$; Welch *t* test). Here, two-way ANOVA showed significant interaction between NTD and γ-2 presence ($F_{1,\ 86} = 10.41$, $P = 0.0018$).

E. Pooled data for the kainate efficacy assay, comparing amplitude of responses to kainate and L-glutamate (KA/Glu ratio; both 500 μM, in the presence of 100 μM cyclothiazide), presented and analysed as in C. No difference between GluA2 wt and ΔNTD either in absence ($P = 0.2064$; n = 6 for both) or presence ($P = 0.6105$; n = 8 for both) of γ-2. Two-way ANOVA showed no significant interaction between NTD and γ-2 presence ($F_{1,\ 24} = 9.733 \times 10^{-4}$, $P = 0.9754$).

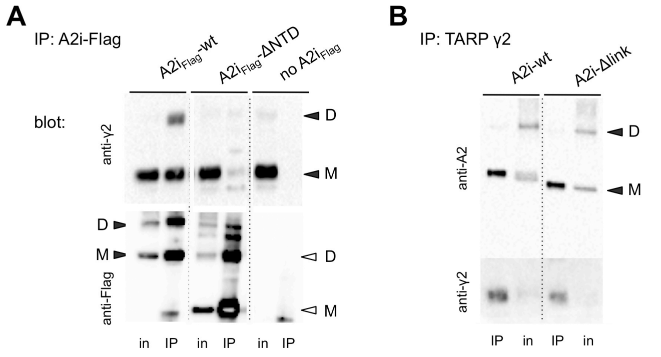

Figure S3. Immunoprecipitation (IP) of TARP γ-2 with GluA2i mutants, related to Figure 4.

A. Representative IP of γ-2 with GluA2i-wt (left) and GluA2i-ΔNTD (right). The blot was probed with polyclonal anti γ-2 (top panel) and anti-Flag antibody to detect GluA2i. Substantially less γ-2 precipitated with the ΔNTD mutant. Both wt and ΔNTD GluA2i migrated as monomer (M) and dimer (D), denoted by arrowheads. Note that inputs were comparable, while amounts of IPed TARP γ-2 varied between conditions.

B. Representative IP of GluA2i-wt (left) and GluA2i-Δlink (right) with γ-2. There was no visible difference between GluA2i-wt and the Δlink mutant with regard to TARP γ-2 association. The lower panel show that comparable amounts of γ-2 were present in the two reactions.

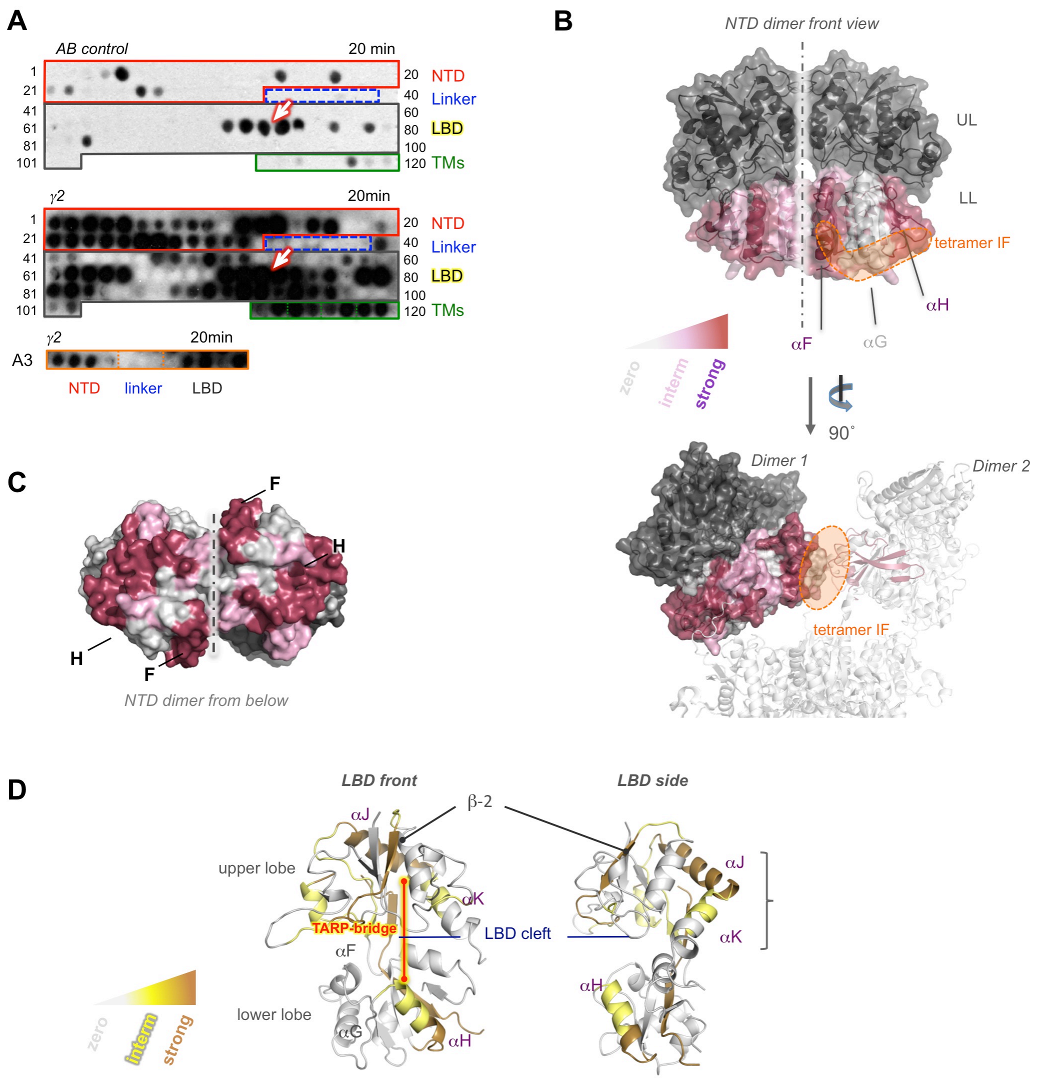

Figure S4. Mapping the TARP γ-2 contact region on GluA2, related to Figure 4.

A. Regions of GluA2 and GluA3 AMPARs binding to TARP γ-2. Array membrane containing GluA2 and -A3 peptides was probed with anti-γ-2 antibody only (AB control, top panel), or incubated with purified γ-2 protein before AB-probing (lower panel) and exposed for 20 minutes. Note the absence of γ-2 signal across the GluA2 linker region (blue box). The NTD-binding region is boxed in red, the LBD region in grey and the TM segments in green. Peptide numbers are indicated on the sides. Lower panel shows the binding of TARP γ-2 to the GluA3 region

flanking the NTD-LBD linker. Binding is observed for the GluA3 NTD and LBD but not for the linker peptides. Non-specific signals are indicated by the tilted white arrow.

B. TARP γ-2 interaction sites on the GluA2 NTD. Contact sites are shown in deep-red (strong interaction) or light pink (weaker interaction). The orange footprint outlines the tetrameric interface that is formed between two NTD dimers in crystal structures. The bottom panel shows the GluA2 NTD dimer superposed on the full-length structure (PDB: 3KG2), with the tetrameric interface (between the NTD dimers) coloured in orange. The stippled line denotes the NTD dimer interface. Upper lobe (UL), lower lobe (LL).

C. TARP γ-2 interactions with the NTD "floor" (a potential NTD-LBD interface). The relative strength of binding is indicated by the same colour gradient as in panel B. The positions of helices H and F are indicated. The stippled line denotes the dimer interface.

D. TARP γ-2 interactions with the LBD. Structural elements involved in the TARP-interaction are shown in brown (strong interaction) and yellow (weaker interaction) in front view (left) and side view (right). Contacts spanning the ligand-binding cleft region are indicated by the red line ('TARP bridge') and involve β-2 in the LBD upper lobe and helix H in the lower lobe. LBD helices J and K are the target of alternative splicing (flip-flop).

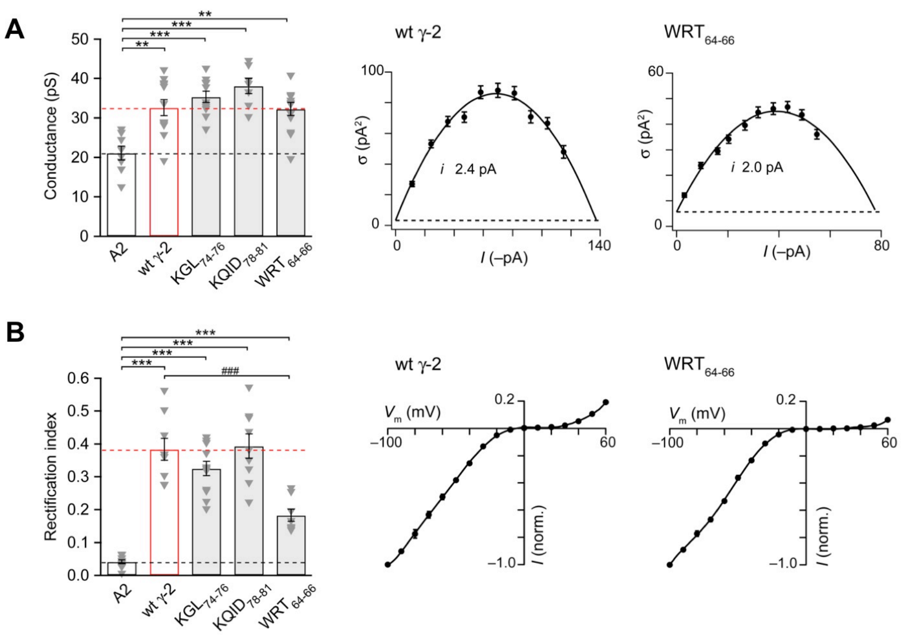

Figure S5. Effects of Ex1 mutations in TARP γ-2 on GluA2 channel conductance and rectification, related to Figure 6.

A. Pooled data (mean ± SEM) showing the effects of Ex1 mutations in γ-2 on weighted mean single-channel conductance from NSFA (10 mM L-glutamate, 100 ms, –60 mV). Values were compared using one-way ANOVA (Welch heteroscedastic *F* test: $F_{4,\,21.02} = 12.26$, $P = 2.67 \times 10^{-5}$). Pairwise comparisons showed that γ-2 wt (n = 12) and all three γ-2 mutants ($KGL_{74\text{-}76}$, $KQID_{78\text{-}81}$ and $WRT_{64\text{-}66}$; n = 11, 7 and 13, respectively) increased channel conductance compared to that of GluA2 alone (n = 8) (** $P < 0.01$, *** $P < 0.001$) (two-sided Welch two-sample *t*-tests with Holm's sequential Bonferroni correction for multiple comparisons). For each of the mutants, the conductance was not statistically different from that seen with γ-2 wt. Shown to the right are representative individual current-variance plots for GluA2/ γ-2 wt and

GluA2/ γ-2 $WRT_{64\text{-}66}$. The single channel current (*i*) is derived from the fitted curve (solid line); the dashed line indicates the background variance.

B. Pooled data showing the effects of mutations in γ-2 on the rectification index (RI) of GluA2. Presentation and analysis as in A ($F_{4, 18.16} = 79.31$, $P = 3.02 \times 10^{-11}$; *** $P < 0.001$ compared to GluA2 alone and $^{\#\#\#} P < 0.001$ compared to γ-2 wt). Shown to the right are representative normalized *I-V* plots from patches expressing GluA2/ γ-2 wt and GluA2/ γ-2 $WRT_{64\text{-}66}$. Fitted curves are 8th order polynomials. Note that $WRT_{64\text{-}66}$ produced less change in RI than did γ-2 wt. This change in RI appeared to reflect changes in spermine permeation, as the effects of $WRT_{64\text{-}66}$ were evident in the outward limb of the *I-V* but not apparent at negative voltages, where the conductance-voltage plots were essentially indistinguishable (data not shown).

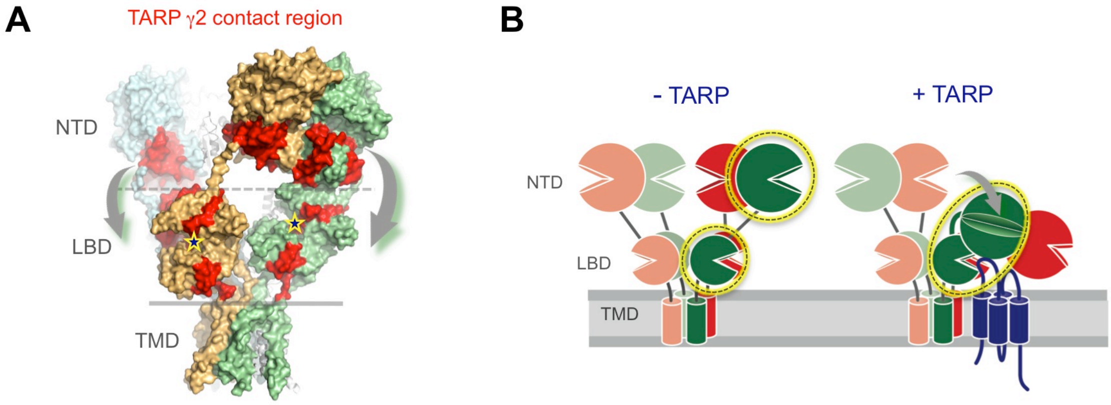

Figure S6. TARP contacts with the NTD may require AMPAR reorganization, related to Figure 4.

A. TARP γ-2 contact region mapped onto the GluA2 tetramer (PDB: 3KG2). Binding sites on the NTD, LBD and LBD-TMD linkers are indicated in red. The position where L-glutamate binds is indicated with stars. The potential movement NTDs need to undergo to contact the TARPs is denoted with grey arrows.

B. Model outlining the reorganization in the AMPAR extracellular region that may accompany TARP interaction. Left panel: Without TARP, the NTD and LBD are loosely connected. Any potential allosteric signal emanating from the NTD is not transmitted to the LBD (and the receptor), i.e. the NTD is functionally isolated (yellow circles). Right panel: The TARP 'bridges' NTD and LBD. This requires substantial receptor reconfiguration mediated by the NTD-LBD linkers. As a result the NTD and LBD are functionally connected (yellow ellipsoid).

GluA2 NTD

1	DLKGALLSLIEYYQW
2	ALLSLIEYYQWDKFA
3	LIEYYQWDKFAYLYD
4	YQWDKFAYLYDSDRG
5	KFAYLYDSDRGLSTL
6	LYDSDRGLSTLQAVL
7	RGLSTLQAVLDSAAE
8	AVLDSAAEKKWQVTA
9	AEKKWQVTAINVGNI
10	WQVTAINVGNINNDK
11	AINVGNINNDKKDET
12	GNINNDKKDETYRSL
13	KDETYRSLFQDLELK
14	YRSLFQDLELKKERR
15	DLELKKERRVILDCE
16	KKERRVILDCERDKV
17	RVILDCERDKVNDIV
18	DCERDKVNDIVDQVI
19	VNDIVDQVITIGKHV
20	DQVITIGKHVKGYHY
21	IGKHVKGYHYIIANL
22	VKGYHYIIANLGFTD
23	HYIIANLGFTDGDLL
24	ANLGFTDGDLLKIQF
25	FTDGDLLKIQFGGAN
26	LLKIQFGGANVSGFQ
27	LKIQFGGANVSGFQI
28	TINIMELKTNGPRKI
29	INIMELKTNGPRKIG
30	IMELKTNGPRKIGYW
31	KTNGPRKIGYWSEVD
32	RKIGYWSEVDKMVVT

GluA2 Linker

33	YWSEVDKMVVTLTEL
34	VDKMVVTLTELPSGN
35	VVTLTELPSGNDTSG
36	TELPSGNDTSGLENK
37	NDTSGLENKTVVVTT
38	GLENKTVVVTTILES
39	KTVVVTTILESPYVM

GluA2 LBD

40	VTTILESPYVMMKKN
41	LESPYVMMKKNHEML
42	MMKKNHEMLEGNERY
43	NHEMLEGNERYEGYC
44	LEGNERYEGYCVDLA
45	ERYEGYCVDLAAEIA
46	GYCVDLAAEIAKHCG
47	DLAAEIAKHCGFKYK
48	EIAKHCGFKYKLTIV
49	CGFKYKLTIVGDGKY
50	YKLTIVGDGKYGARD
51	IVGDGKYGARDADTK
52	GKYGARDADTKIWNG
53	ARDADTKIWNGMVGE
54	DTKIWNGMVGELVYG
55	WNGMVGELVYGKADI
56	VGELVYGKADIAIAP
57	VYGKADIAIAPLTIT
58	ADIAIAPLTITLVRE
59	IAPLTITLVREEVID
60	TITLVREEVIDFSKP
61	REEVIDFSKPFMSLG
62	IDFSKPFMSLGISIM
63	KPFMSLGISIMIKKP
64	LGISIMIKKPQKSKP
65	GISIMIKKPQKSKPG
66	VERMVSPIESAEDLS
67	MVSPIESAEDLSKQT
68	IESAEDLSKQTEIAY
69	EDLSKQTEIAYGTLD
70	KQTEIAYGTLDSGST
71	YGTLDSGSTKEFFRR
72	SGSTKEFFRRSKIAV
73	KEFFRRSKIAVFDKM
74	RRSKIAVFDKMWTYM
75	IAVFDKMWTYMRSAE
76	DKMWTYMRSAEPSVF
77	YMRSAEPSVFVRTTA
78	AEPSVFVRTTAEGVA
79	VFVRTTAEGVARVRK
80	TTAEGVARVRKSKGK
81	GVARVRKSKGKYAYL
82	VRKSKGKYAYLLEST
83	KYAYLLESTMNEYIE
84	LLESTMNEYIEQRKP
85	NEYIEQRKPCDTMKV
86	EQRKPCDTMKVGGNL
87	CDTMKVGGNLDSKGY
88	KVGGNLDSKGYGIAT
89	NLDSKGYGIATPKGS
90	KGYGIATPKGSSLGN
91	IATPKGSSLGNAVNL
92	KGSSLGNAVNLAVLK
93	LGNAVNLAVLKLNEQ
94	VNLAVLKLNEQGLLD
95	VLKLNEQGLLDKLKN
96	NEQGLLDKLKNKWWY
97	LLDKLKNKWWYDKGE
98	LKNKWWYDKGECGSG
99	WYDKGECGSGGGDSK
100	GECGSGGGDSKEKTS
101	GGGDSKEKTSALSLS
102	GGDSKEKTSALSLSN

GluA3 Linker

103	MKVSGSRKAGYWNEY
104	GSRKAGYWNEYERFV
105	AGYWNEYERFVPFSD
106	NEYERFVPFSDQQIS
107	VPFSDQQISNDSSSS
108	DQQISNDSSSSENRT
109	NDSSSSENRTIVVTT
110	SSENRTIVVTTILES
111	RTIVVTTILESPYVM
112	VTTILESPYVMYKKN

GluA2 TMs

113	YEIWMCIVFAYIGVS
114	VFAYIGVSVVLFLVS
115	FGIFNSLWFSLGAFM
116	SLWFSLGAFMQQGCD
117	VWWFFTLIIISSYTA
118	IIISSYTANLAAFLT
119	VAGVFYILVGGLGLA
120	VGGLGLAMLVALIEF

Table S1. Sequences of peptides immobilized in the GluA2 array, related to Figure 4.

Peptides include residues 107-238, 348-510, 520-541, 568-587 and 601-811 of mature GluA2o. Peptide numbering and colour code match Figure 4 and S4. Array also includes residues 360-413 of the GluA3 NTD-LBD linker (shown in Figure S4A).

γ-2		γ-8	
	**Ex1**		**Ex1**
1	DYWLYSRGVCKTKSV	22 (1)	STDYWLYTRALICNT
2	YSRGVCKTKSVSENE	23 (2)	WLYTRALICNTTNLT
3	VCKTKSVSENETSKK	24 (3)	RALICNTTNLTAGDD
4	KSVSENETSKKNEEV	25 (4)	CNTTNLTAGDDGPPH
5	ENETSKKNEEVMTHS	26 (5)	NLTAGDDGPPHRGGS
6	SKKNEEVMTHSGLWR	27 (6)	GDDGPPHRGGSGSSE
7	EEVMTHSGLWRTCCL	28 (7)	PPHRGGSGSSEKKDP
8	THSGLWRTCCLEGNF	29 (8)	GGSGSSEKKDPGGLT
9	LWRTCCLEGNFKGLC	30 (9)	SSEKKDPGGLTHSGL
10	CCLEGNFKGLCKQID	31 (10)	KDPGGLTHSGLWRIC
11	GNFKGLCKQIDHFPE	32 (11)	GLTHSGLWRICCLEG
12	GLCKQIDHFPEDADY	33 (12)	SGLWRICCLEGLKRG
13	QIDHFPEDADYEADT	34 (13)	RICCLEGLKRGVCVK
14	FPEDADYEADTAEYF	35 (14)	LEGLKRGVCVKINHF
15	ADYEADTAEYFLRAV	36 (15)	KRGVCVKINHFPEDT
16	YEADTAEYFLRAVRA	37 (16)	CVKINHFPEDTDYDH
		38 (17)	NHFPEDTDYDHDSAE
		39 (18)	EDTDYDHDSAEYLLR
		40 (19)	YDHDSAEYLLRVVRA
		41 (20)	DHDSAEYLLRVVRAS
	**Ex2**		**Ex2**
17	VYISANAGDPSKSDS	42 (21)	VYISANAGEPGPKRD
18	ANAGDPSKSDSKKNS	43 (22)	ANAGEPGPKRDEEKK
19	DPSKSDSKKNSYSYG	44 (23)	EPGPKRDEEKKNHYS
20	SDSKKNSYSYGWSFY	45 (24)	KRDEEKKNHYSYGWS
21	DSKKNSYSYGWSFYF	46 (25)	DEEKKNHYSYGWSFY

Table S2. Sequences of peptides immobilized in the TARP arrays, related to Figure 5. Ex1 and Ex2 denote the two extracellular loops of TARPs. Peptide numbering matches Figure 5, which contains both TARPs in the same membrane. Numbers in parentheses correspond to peptides in 5C (central panel), which only contains γ-8. Colour code as in Figure 5.

Supplemental Experimental Procedures

Protein preparation

His-tagged γ-2 was produced in insect cells using a P1 baculovirus stock and a purification protocol provided by T. Nakagawa. The high-titer viral stocks were obtained following the Bac-N-Blue™ protocol from Invitrogen. Briefly, 1 x 10^6 SF9 cells were plated into a 25 cm^2 flask containing 5 ml of complete TNM-FH medium (Sigma Aldrich) and infected with 20 μl of the P1 viral stock. The flask was incubated at 27 °C until all the cells were lysed; 4 ml of this supernatant (the P2 viral stock) were used to infect 500 ml of SF9 cells at a density of 1 x 10^6 SF9 cells/ml. The culture was incubated for 1 week and the high-titer P3 viral stock was recovered by centrifugation and kept at 4 °C until infection. For protein production, 500-2000 ml of SF9 cells grown in SF900™ II serum-free medium (Gibco®) were infected with P3 high-titer viral stock and incubated for 48-72 hours. Cells were collected by centrifugation and re-suspended in 20 mM HEPES, pH 7.35, 320 mM sucrose, 5 mM EDTA, 5 mM EGTA, protease inhibitors (Roche) using a Dounce homogenizer. Cells were sonicated and membranes were washed in consecutive steps of re-suspension and ultracentrifugation using 1 M KI, 4 M urea and 20 mM imidazole in 20 mM HEPES, pH 7.35. Membrane protein solubilization was performed in 0.6 % decyl-maltoside (DM). After ultracentrifugation, the supernatant containing the His-tagged γ-2 was incubated with chelating sepharose beads (GE Healthcare) charged with Co^{2+} and the protein was eluted with 250 mM imidazole, 0.3 % DM, 20 mM HEPES, pH 7.35, 150 mM NaCl. Protein was concentrated, flash-frozen and conserved at -20 °C.

γ-2 concentration was estimated spectrophotometrically using the theoretical extinction coefficient in water, 39.2 mM^{-1} cm^{-1}.

The GluA2 NTD was produced and purified from stably transfected $GntI^{-}$ HEK293S cells as described previously (Rossmann et al., 2011). The purification consisted of cross-flow concentration and dialysis against 50 mM Tris pH 8, 150 mM NaCl; affinity purification with a HisTrap HP column (GE Healthcare) and gel filtration using a HiLoad™ Superdex™ 200 column (GE Healthcare). The pure glycosylated GluA2 NTD was concentrated in 20 mM HEPES, 150 mM NaCl, pH 7.4 and kept at 4 °C.

The GluA2i LBD (S1S2J R/flip construct) was sub-cloned with different tags in order to allow its detection in the peptide arrays. GluA2i LBD was first sub-cloned into the pGEX4T-2 vector (GE Healthcare), which contains an N-terminal GST-tag followed by a thrombin cleavage site. The plasmid was transformed into *Escherichia coli* Origami B (DE3) and grown at 37 °C to A_{600}=0.9-1. Cultures were cooled to 18 °C and expression was induced by the addition of 0.4 mM IPTG. Cultures were grown at 18 °C for 20 hours. Cells were sonicated and the lysate was incubated with Glutathione Sepharose 4b beads (GE Healthcare). The protein was eluted with 10 mM reduced-Glutathione and further purified using a Superdex 200 10/300 column (GE Healthcare). Protein was flash-frozen and kept at -20 °C until used.

A Flag-tag (DYKDDDDK) was also introduced by PCR at the C-terminus of GluA2i LBD and the gene was subcloned into a modified pET22b(+) plasmid containing an N-terminal His_8 affinity tag and a thrombin cleavage site. The plasmid was

transformed into *E. coli* Origami B (DE3) cells and cultures were grown at 37 °C until A_{600} reached 0.6–0.9. Cells were cooled to 18 °C and over-expression was induced with 0.4 mM IPTG for 20 hours. After cell lysis, protein was purified by affinity chromatography using a HisTrap HP column (GE Healthcare) followed by thrombin cleavage and gel filtration chromatography (HiLoad™ Superdex™ 200 column, GE Healthcare).

The same procedure was used to add a Flag-tag to the GluK2 LBD S1S2 construct (provided by Mark L. Mayer). The resulting protein includes a 19 peptide (MH_8 SSGLVPRGSAM) containing a thrombin cleavage site, the residues S398–K513 and P636–E775 of GluK2 connected by a GT linker and the Flag-tag at the C-terminus (DYKDDDDK). The protein production and purification protocol was the same as described for the GluA2i LBD Flag. Both Flag-tagged proteins were concentrated in 20 mM HEPES, 150 mM NaCl, pH 7.4, 1 mM L-glutamate and conserved at -20 °C.

Immunoprecipitations

HEK293 cells were transfected with vectors expression GluA2-IRES-EGFP and TARP γ-2 (IRES-RFP) at a ratio of 4:1, alternatively GluA2 was transfected into HEK cells stably expressing γ-2. After 2 days of expression, cells were washed with cold PBS, scraped into PBS containing protease inhibitors and centrifuged for 10 mins at 10K rpm for in an Eppendorf 5424R. Pellets were extracted in 1% CHAPS buffer containing in (mM): HEPES (20; pH 7.4), NaCl (150), EDTA (2), 1% CHAPS and protease inhibitors for 45 mins at 4˚C. Extracts were centrifuged for 30 min at 4˚C in an Eppendorf 5424R centrifuge at full speed. Supernatants were IPed with 5 µg of anti- γ-2 AB (Millipore) for 2 hrs at 4˚C and precipitated with 10 µl of equilibrated

(in CHAPS buffer) protein A beads (Santa Cruz). Lysates were run on 4-12% Bolt gels (Invitrogen) and gels analyzed by Western blotting.

Peptide arrays

To analyze the interaction between AMPAR and TARPs we obtained peptide arrays synthesized by SPOT synthesis on Whatman 50 cellulose membranes (PepSpots™; JPT Peptide Technologies GmbH). AMPAR and TARP arrays contained 15-mer overlapping peptides shifted by 4 residues. The AMPAR array contained 128 peptides covering the lower lobe of GluA2 NTD, the NTD-LBD linkers with and without glycosylated residues (GluA2 and -A3 subunits), the GluA2 LBD and the four trans-membrane helices (**Figure 4B, Table S1**). The TARP array contained 46 peptides covering the two extracellular loops (Ex1 and Ex2) of γ-2 and γ-8 (**Figure 5, Table S2**).

Membranes were probed according to manufacturer instructions. As these membranes cannot be regenerated reliably, control experiments with the antibodies (ABs) were performed prior to incubation with the test proteins. Controls were essential and extensive to select the most adequate blocking agents, antibodies and protein tags, as some ABs showed false positives or high background in the cellulose membranes. Anti-His and anti-GST ABs showed signals in the control experiments whereas Flag M2 AB (Sigma), anti-AMPAR 2 (extracellular) AB (Alomone) or anti-Stargazin AB (Millipore) controls showed few false positives (**Figures 4C and 5B**).

For the protein binding assay, membranes were rinsed with methanol for 5 minutes, washed 3 times with TBS and incubated with blocking solution (5 % BSA in TBS)

for 2 hours at RT or overnight at 4 °C. After blocking, arrays were incubated with the test proteins overnight at 4 °C. The AMPAR array was incubated with ~2.8 μM γ-2 in blocking solution with 0.15 % DM. TARP arrays were incubated with ~1.6 μM GluA2 NTD, ~1.6 μM GluA2i LBD-Flag or ~1.6 μM GluK2 LBD-Flag in blocking solution. Membranes were washed three times in TBS and incubated for 1-2 hours at RT with the following ABs: anti-AMPAR 2 (extracellular) AB (Alomone) diluted 1:250 in blocking solution; anti-Stargazin AB (Millipore) diluted 1:1000 in blocking solution; and monoclonal anti-Flag M2 antibody (Sigma) diluted 1:1000 in blocking solution. After 3 washes with TBS membranes were incubated for 1 hour at RT with HRP-conjugated anti-rabbit AB (Pierce) (1:1500 in blocking buffer) or 2 hours at RT with HRP-conjugated anti-mouse AB (Pierce) (1:1000 in blocking buffer). Membranes were washed 3 times, developed using chemiluminescence (Amersham ECL solution) and images were captured electronically with a ChemiDoc™ MP Imaging System (Biorad) or on X-ray film. Films were scanned and edited with Photoshop CS4 using auto-tone editing followed by a change to gray-scale mode.

Heterologous expression

HEK293 or HEK293T cells (ATCC), cultured at 37°C and 5% CO_2 in DMEM (Gibco) supplemented with 10% fetal bovine serum and penicillin/streptomycin, were transiently transfected with plasmid DNA using Effectene (Qiagen) or Lipofectamine 2000 (Invitrogen). After 24-32 hours (Effectene) or 8-14 hours (Lipofectamine 2000), the cells were split onto poly-L-lysine-coated glass coverslips and electrophysiological recordings were performed 12-48 hours later. When co-transfected with TARPs, cells were grown in the presence of 30-50 μM 2,3-dioxo-6-

nitro-1,2,3,4-tetrahydrobenzo[f]quinoxaline-7-sulfonamide (NBQX; Tocris-ABCam) to avoid AMPAR-mediated toxicity.

Electrophysiology

Cells were visualized with an inverted microscope (Diaphot 200; Nikon) or fixed stage upright microscope (Axioskop FS1; Zeiss) and perfused with an 'external' solution containing (in mM): NaCl (145), KCl (3), $CaCl_2$ (2), $MgCl_2$ (1), glucose (10) and HEPES (10), adjusted to pH 7.4 with NaOH. Electrodes were fabricated from borosilicate glass (1.5mm o.d., 0.86mm i.d., Science Products GmbH or Harvard Apparatus) pulled with a PC-10 vertical puller (Narishige). When filled with an 'internal' solution, containing (mM): CsF (120), CsCl (10), EGTA (10), ATP-sodium salt (2), and HEPES (10), adjusted to pH 7.3 with CsOH, they had a final resistance of 2-5 MΩ. Macroscopic currents were recorded at room temperature (RT; 22-25 ˚C) from outside-out patches excised from GFP-positive cells and voltage-clamped at –60 mV. Currents were recorded with Axopatch 1D, 200A or 2D amplifiers, low-pass filtered at 10 kHz and digitized at 50 kHz using a Digidata 1440A interface with pClamp 9 or 10 software (Molecular Devices).

For the experiments described in **Figures 6 and S5** there were minor modifications of some experimental details; these reflected the established protocols of the two participating labs and, where compared directly, did not affect the outcome. For the experiments on TARP Ex1 mutants (**Figures 6 and S5**) the external solution contained (in mM) NaCl (145), KCl (2.5), $CaCl_2$ (1), $MgCl_2$ (1), glucose (10) and HEPES (10), adjusted to pH 7.3 with NaOH. The internal solution contained (in mM) CsCl (145), EGTA (1), ATP-magnesium salt (4), NaCl (2.5) and HEPES (10), adjusted to pH 7.3 with CsOH. Electrodes were coated with Sylgard (Dow Corning

184) and had a final resistance of 6-14 MΩ. Aside from experiments in which KA/Glu ratios were measured, spermine tetrahydrochloride (100 μM; Tocris Bioscience or Sigma Aldrich) was added to the internal solution.

Agonist application to excised patches

Rapid agonist application was achieved by switching between a continuously flowing control solution and an agonist-containing solution, applied via a theta-barrel application tool made from borosilicate glass (2mm o.d.; Hilgenberg GmbH) pulled to a tip diameter of ~200 μm and mounted on a piezoelectric translator (Burleigh PZS 200 or LSS-3000/PZ-150M; EXFO Life Sciences & Industrial Division). At the end of each experiment, the adequacy of the solution exchange was assessed by destroying the patch and measuring liquid-junction current at the open pipette (typical 10% - 90% solution exchange time of ~200 μs).

Kinetics of AMPAR-mediated responses

Desensitization of AMPARs was examined in response to 100 ms applications of 10 mM L-glutamate. The averaged currents were fitted using a double-exponential function to calculate the weighted time constant of desensitization ($\tau_{w,des}$) according to:

$$\tau_{w,des} = \tau_f\left(\frac{A_f}{A_f + A_s}\right) + \tau_s\left(\frac{A_s}{A_f + A_s}\right)$$

where A_f and τ_f are the amplitude and time constant of the fast component of desensitization and A_s and τ_s are the amplitude and time constant of the slow

component of desensitization. The weighted time constant of deactivation ($\tau_{w,deact}$) was determined in a similar manner, by fitting the current decay following 1 ms applications of 10 mM L-glutamate. In some cases the desensitization or deactivation time course was best described by a single exponential. The steady state-to-peak ratio (SS/peak) was determined as the current at the end of the 100 ms pulse divided by the peak current.

Recovery from desensitization was assessed by a paired-pulse protocol where a 100 ms desensitizing pulse was followed by a 10 ms pulse in increasing intervals. The relative response to the second pulse (usually the average of three consecutive runs) was then plotted against time elapsed from the first pulse and the time course fitted with a single-exponential function to obtain the time constant of recovery (τ_{rec}).

Relative kainate efficacy

The effects of TARPs on the efficacy of the partial agonist kainate (KA) were determined by measuring changes in KA/Glu ratios. Each patch was exposed to L-glutamate (500 μM; 15 applications of 100 ms duration, –60 mV), then KA (500 μM), and then L-glutamate again. Both control and agonist solutions contained 100 μM cyclothiazide to block AMPAR desensitization. The amplitude of the steady state current response to KA was compared to average amplitude of the steady state current responses to L-glutamate obtained before and after KA application.

Non-stationary fluctuation analysis (NSFA)

To deduce channel properties from macroscopic responses, L-glutamate (10 mM) was applied to outside-out patches (100 ms duration, 1 Hz, V_{hold} –60mV) and the ensemble

variance of all successive pairs of current responses were calculated (Conti et al., 1980). The single channel current (*i*) and the total number of channels in the patch (*N*) were determined by plotting this ensemble variance against mean current (I) and fitting with a parabolic function (Sigworth, 1980):

$$\sigma^2 = \sigma_B^2 + \left(i\bar{I} - \left(\frac{\bar{I}^2}{N}\right)\right)$$

where σ^2_B is the background variance. The weighted-mean single-channel conductance was determined from the single-channel current and the holding potential. No correction for liquid-junction potential was used.

Current-voltage (*I-V*) plots and the quantification of rectification

I-V plots were generated from the peak current response to 1 ms applications of 10 mM glutamate. The voltage was stepped from –100 mV to +60 mV in 10 mV increments. Mean current amplitudes at each voltage were normalized to the peak current at –100 mV and plotted against membrane potential. The relationships were fitted with 8th or 9th order polynomials. The rectification index (RI) was determined from the *I-V* relationship of each patch as the ratio of slope conductance at positive (+40 mV to +60 mV) and negative voltages (–40 mV to –60 mV). Thus, for a completely linear *I-V* relationship RI would equal 1.

Analysis and statistics

Recordings were analyzed using IGOR Pro (Wavemetrics Inc.) with NeuroMatic (J. Rothman, UCL; http://www.neuromatic.thinkrandom.com). Summary data are presented in the text as the mean ± SEM from *n* patches and in the figures as bar plots of the group mean, with error bars denoting SEM. Comparisons involving two data

sets only were performed using a two-sided Welch two-sample t test. All analyses involving data from three or more groups were performed using one- or two-way analysis of variance (Welch heteroscedastic F test) followed by pairwise comparisons using two-sided Welch two-sample t tests (with Holm's sequential Bonferroni correction for multiple comparisons). Differences were considered significant at $P < 0.05$. Statistical tests were performed using Prism 4.0/6.0 (GraphPad Software Inc.) or R (version 3.0.2, The R Foundation for Statistical Computing, http://www.r-project.org/) and RStudio (version 0.98.313, RStudio, Inc.).

Supplemental References

Conti, F., Neumcke, B., Nonner, W., and Stampfli, R. (1980). Conductance fluctuations from the inactivation process of sodium channels in myelinated nerve fibres. J Physiol *308*, 217-239.

Sigworth, F.J. (1980). The variance of sodium current fluctuations at the node of Ranvier. J Physiol *307*, 97-129.
